# Benzoxazinoid‐mediated microbiome feedbacks enhance Arabidopsis growth and defence

**DOI:** 10.1111/nph.71098

**Published:** 2026-03-17

**Authors:** Katja Stengele, Lea Stauber, Lisa Thoenen, Henry Janse van Rensburg, Viola D'Adda, Klaus Schlaeppi

**Affiliations:** ^1^ Department of Environmental Sciences University of Basel 4056 Basel Switzerland; ^2^ Institute of Plant Sciences University of Bern 3013 Bern Switzerland

**Keywords:** *Arabidopsis thaliana*, *Botrytis cinerea*, microbiome feedback, plant–soil feedback, root microbiota, secondary metabolites

## Abstract

- Plants modulate their surrounding microbiome via root exudates and such conditioned soil microbiomes feed back on the performance of the next generation of plants. How plants perceive altered soil microbiomes and modulate their performance in response to such microbiome feedbacks, however, remains largely unknown.
- As tool to condition contrasting microbiomes in soil, we made use of two maize lines, which differ in their ability to exude benzoxazinoids (BXs). Based on these differentially conditioned soil microbiomes we have established a model system with *Arabidopsis thaliana* (Arabidopsis) to investigate the mechanisms of microbiome feedbacks.
- Arabidopsis plants responding to the BX‐conditioned soil microbiome grew better and were developmentally more advanced. Further, these plants harboured differential root bacterial communities, showed enhanced defence signatures in transcriptomes of their shoots, and they were more resistant to the fungal pathogen *Botrytis cinerea*.
- Intriguingly, Arabidopsis responded with both improved growth and enhanced defence to the BX‐conditioned soil microbiome, and we found that this simultaneous increase of growth and defence was mediated by priming of the defences. Further advancing our basic understanding of how plants respond to soil microbiomes and mediate their feedbacks is particularly important for the goal to improve crops so they can benefit from their soil microbiome.

## Introduction

Plants condition the surrounding soil throughout their lifetime to optimise their immediate environment by changing the biotic and abiotic properties of the soil they grow in (Van der Putten *et al*., 2013). These soil changes also persist and influence the performance of a next generation of plants, which is known as a plant–soil feedback (PSF; Bezemer *et al*., 2006; Van der Putten *et al*., 2013; Mariotte *et al*., 2018). In the conditioning phase of PSFs, the growing plants alter not only the physiochemical soil properties such as soil structure, organic matter content or nutrient levels, but they also alter the specific metabolite profile (Delory *et al*., 2024), and the biotic soil properties including the soil microbiome (Jing *et al*., 2022; Semchenko *et al*., 2022). These soil alterations then ‘feed’ back on the growth and/or defence of the next plant generation. If these feedbacks can be specifically assigned to altered soil microbiomes, which the plant perceives and responds to, we refer to them as ‘microbiome feedbacks’ (Janse van Rensburg *et al*., 2024).

The microorganisms living in soil also present the majority of the microorganisms that colonise plant roots (Spooren *et al*., 2024). These microorganisms collectively function as a microbiome, which includes bacteria, fungi, viruses and protists (Bai *et al*., 2022). The root microbiome affects plant health and performance (Bulgarelli *et al*., 2013; Trivedi *et al*., 2020), for example, by regulating plant nutrient homeostasis (Salas‐González *et al*., 2021), mitigating abiotic stress (Schmitz *et al*., 2022) or protecting against pathogens (Durán *et al*., 2018). To foster such beneficial functions, plants can selectively modulate the composition and activity of their root and surrounding soil microbiome through the secretion of root exudates (Sasse *et al*., 2018; Jacoby *et al*., 2021). These exudates include primary and secondary metabolites, which act as nutrient sources or specific communication cues to microbes (Sasse *et al*., 2018; Korenblum *et al*., 2022). Several specific classes of exuded metabolites have been demonstrated to structure the plant root and rhizosphere microbiota, including coumarins (Stringlis *et al*., 2018b), flavones (Yu *et al*., 2021), and benzoxazinoids (BXs; Hu *et al*., 2018; Cotton *et al*., 2019; Kudjordjie *et al*., 2019).

BXs are indole‐derived alkaloids that function as secondary metabolites, and they are mainly produced by Poaceae, including maize, wheat, and rye (Niemeyer, 2009; Niculaes *et al*., 2018). BXs are multifunctional and act, for instance, in the aboveground defence against generalist herbivores (Wouters *et al*., 2016; Robert & Mateo, 2022). BXs are also exuded in substantial amounts into soil, particularly by maize (Hu *et al*., 2018), where the main exuded metabolites are 2,4‐dihydroxy‐7‐methoxy‐1,4‐benzoxazin‐3‐one (DIMBOA) and its glucoside. DIMBOA is spontaneously converted into 6‐methoxybenzoxazolin‐2(3H)‐one (MBOA) in soil, which is then further metabolised to 2‐amino‐7‐methoxyphenoxazin‐3‐one (AMPO) by microbes (Niemeyer, 2009). In soil, BXs have important belowground functions with their allelopathic effects on neighbouring or successional plants (Teasdale *et al*., 2012; Schandry & Becker, 2020). Typical phytotoxic compounds are the BX breakdown products 2‐amino‐3H‐phenoxazin‐3‐one (APO) and AMPO, which have been shown to inhibit the growth of Arabidopsis *in vitro* (Venturelli *et al*., 2015).

BXs also have important belowground functions on the microbiota in PSFs. The root and rhizosphere microbiotas are selectively shaped by the exuded BXs (Hu *et al*., 2018; Cotton *et al*., 2019; Kudjordjie *et al*., 2019), which leaves a legacy in soil for a next plant generation. This BX‐dependent soil conditioning was found to affect growth and defence of a next generation of maize (Hu *et al*., 2018) or wheat (Cadot *et al*., 2021). Sterilisation and microbiota complementation experiments demonstrated that these feedbacks were mediated by the microbes, and supplementation of MBOA to non‐BX exuding maize plants established that soil conditioning was BX‐dependent (Hu *et al*., 2018). These BX‐mediated feedbacks are studied by comparing soil conditioned with wild‐type maize plants that exude BXs, called ‘BX_plus_’ soil, to the control ‘BX_minus_’ soil that is conditioned by the BX‐defective mutant line *bx1* (Maag *et al*., 2016). While the phenology (Hu *et al*., 2018; Cadot *et al*., 2021) and the agronomic relevance (Gfeller *et al*., 2023a, 2023b) of BX‐mediated feedbacks have been well described, the mechanistic understanding of how plants perceive the differential soil microbiomes and how they alter their own performance remains largely unknown. In more general terms, how plants perceive complex soil microbiomes and how they mediate microbiome feedbacks is currently understudied (Janse van Rensburg *et al*., 2024).

In search of a model system to investigate microbiome feedbacks and to facilitate mechanistic studies, we explored BX‐mediated feedbacks on the model plant *Arabidopsis thaliana* Col‐0 (henceforth Arabidopsis), which we grew on BX_plus_ and BX_minus_ soils. To assess these feedbacks on Arabidopsis, we have quantified both vegetative and reproductive growth traits. Using soil sterilization and microbiota analysis, we assessed the role of the soil and Arabidopsis root microbiota in shaping these feedbacks. Further, we have investigated plant responses to the two soil variants using transcriptome analysis, and we have corroborated a defence signature in the shoots by directly testing Arabidopsis' resistance to the necrotrophic pathogen *Botrytis cinerea* (henceforth Botrytis). Lastly, we tested the hypothesis that the BX_plus_ soil microbiota primes Arabidopsis’ defences as a mechanism explaining the better resistance alongside with the improved growth.

## Materials and Methods

### Plant material

For the soil conditionings, we used the maize (*Zea mays* L.) variety B73 and the BX‐deficient line *bx1* in B73 background (Maag *et al*., 2016). For all feedback and *in vitro* experiments, we used the Arabidopsis (L.) Heynh. (Arabidopsis) accession Columbia‐0 (Col‐0).

### Soil conditioning

We collected soil from a field managed by Agroscope in Changins (Nyon, Switzerland) between 2019 and 2022 to condition with maize. Soils were sieved (1 cm mesh size), homogenised, and stored at 4°C before conditioning. For each conditioning event, which yields one ‘soil batch’, we grew wild‐type B73 and *bx1*(B73) maize for 12 wk in individual pots. For details on the setup of each conditioning experiment and soil nutrient concentration of conditioned soil batches see Supporting Information Tables S1 and S2. Plants were watered three times a week and supplemented once a week with nutrient solution (Table S1). After 12 wk, the roots were removed from the pots and the remaining rhizosphere and soil were collected, mixed and pooled by maize genotype, resulting in BX_plus_ soil (i.e. conditioned with B73 wild‐type maize) and BX_minus_ control soil (i.e. conditioned with *bx1*(B73) maize). Soils were stored at 4°C until further use, and previous work demonstrated that storage for up to year at 4°C does not affect the feedback with maize (Cadot *et al*., 2021). In that study, we also characterised the BX_minus_ and BX_plus_ soil microbiomes for subsequent feedback experiments. In brief, while there were marked differences between rhizospheres (soil washed from roots) of wild‐type and *bx1* maize plants at the end of conditioning phase (12 wk in the field or in pots; also shown in Hu *et al*., 2018; Gfeller *et al*., 2023a, 2023b), the soil microbiomes that resulted from mixing these discriminatory rhizospheres into the surrounding soil from the pots (or soil cores in field conditionings) differed only minimally. Importantly, even if there were no major compositional differences between BX_minus_ and BX_plus_ soil collected at whole pot/soil core level, these differences remained sufficient to trigger robust effects in subsequent feedback experiments. In Table S3, we document the conditioned soil batches that were used for each of the Arabidopsis feedback experiments. Additionally, for each feedback experiment where shoot growth was quantified, we have correlated the feedback strength with the soil storage duration. Feedback strength was calculated as the median shoot area of BX_plus_ grown Arabidopsis plants divided by the median shoot area of BX_minus_ grown plants and subtracted by 1, so that positive values represent a positive feedback, that is larger shoot areas on BX_plus_ soil. A lack of correlation indicated that soil storage did not affect the feedback strength (Fig. S1a).

### Feedback experiments in soil

As a general procedure to perform feedback experiments with Arabidopsis, the conditioned soils were sieved (5 mm mesh size), amended with 20% (v/v) autoclaved sand and filled to the same weight into small pots. Arabidopsis seeds were stratified for 2–3 d either directly on the pots or suspended in 0.2% sterile agar before sowing. Several seeds were distributed per pot, and after 1–2 wks, the healthiest looking seedling was kept and the others were removed from the pot. Plants were grown in growth cabinets (CLF Plant Climatics, Wertingen, Germany) at 60% relative humidity, 10 h day at 21°C and light intensities between 100 and 200 μmol m^−2^ s^−1^, and 14 h night at 18°C. Short‐day conditions were chosen because slower plant development would allow longer exposure of roots to the soil microbiome. Pots were watered with tap water three times per week, adjusted to the same weight, and the pot position was changed once per week. Plants were fertilised by watering with 1/3 half‐strength Hoagland solution (Cardoso *et al*., 2014) diluted in tap water. Plants were grown for 6–7 wk before harvesting. Here, we described the general procedure applying to all experiments, while we summarised the different set‐ups of the experiments (Experiments I to XII) in detail in Table S3, and we explain additional details of particular experiments in the Methods S1. For all experiments, rosette area was determined by either analysing scans of cut rosettes with Imagej v.1.5 (Schneider *et al*., 2012) or by analysing photographs of rosettes from living plants with the software Aradeepopsis (Hüther *et al*., 2020).

### *In vitro* experiments

We have performed three *in vitro* experiments (*in vitro* Experiments 1 to 3), two on agar plates and one using a semi‐hydroponic growth system (McLaughlin *et al*., 2023), to test the effects of MBOA on Arabidopsis growth and resistance to Botrytis. The three experiments were conducted using the same Percival growth chambers and the same growth settings as for the feedback experiments in soil. The specific experimental details and measurements are documented in the Methods S2.

### Infections with Botrytis

For infections, the Botrytis strain B05.10 was grown in the dark at 18°C on potato dextrose agar (Merck Millipore, Burlington, VT, USA) for 2–3 wk. Plants were grown as described under *Feedback experiments in soil*. At 5–6 wk, three mature source leaves per plant were infected with Botrytis spores that were harvested from plates with sterile water +0.0001% Tween‐20 (Biorad, Basel, Switzerland), filtered through glass wool and adjusted to 1 × 10^5^ spores ml^−1^ in half‐strength potato dextrose broth (Formedium, Norfolk, UK). For infection, a 5 μl drop was placed onto the middle of the leaf towards the edge. The plant trays were covered with a water‐sprayed transparent lid that was taped to the tray to ensure high humidity. For the infection, plants were transferred to a growth chamber (Sanyo, Moriguchi, Japan) at the same growth conditions as previously, but equipped with fluorescent bulbs of *c*. 100 μmol m^−2^ s^−1^ light intensity. Experiment XI was incubated in a Sanyo chamber equipped with LEDs under low light conditions (*c*. 50 μmol m^−2^ s^−1^) for the infection. Photographs from the infected leaves were taken from the top at a fixed distance 3 d after the infection. The area was quantified using the Fiji distribution of ImageJ (Schindelin *et al*., 2012) by selecting the area of the lesions with the Polygon tool and calculating the area of the lesions.

### Defence priming of *PR1*

The shoots of 6‐wk‐old plants (Experiment VII) were dipped in a 1 mM salicylic acid (SA) solution containing 0.015% Silwet L‐77 or mock treated by dipping in tap water containing 0.015% Silwet L‐77. After 30 min, 4, 6 and 24 h, shoots were collected, snap frozen in liquid nitrogen and stored at −80°C. The whole shoots were ground under liquid nitrogen, and RNA was extracted with the RNeasy plant kit (Qiagen). cDNA was then synthesised with the GoScript cDNA Synthesis kit (Promega), and quantitative real‐time polymerase chain reaction (qRT‐PCR) reactions with the KAPA SYBR® FAST qPCR Master Mix (Kapa Biosystems, Wilmington, DE, USA) were set up for *PR1* and the reference gene *PP2A* for each sample in duplicate. Per 15 μl reaction, 0.3 μl of the *PP2A* primers (Rodriguez *et al*., 2010) and the *PR1* primers (Pieterse *et al*., 1998) were used at 10 μM each. qRT‐PCR was performed on a LightCycler® 480 (Roche, Basel, Switzerland) following the recommended cycling parameters from KAPA with an annealing temperature of 60°C for 25 s followed by fluorescence acquisition for 2 s at 72°C. The data were then processed with the LinRegPCR software (Ruijter *et al*., 2009) and further analysed in R v.4.3.0 (R Core Team, 2017).

### Determination of BX concentrations in soils

Before the first Arabidopsis experiments, soil samples of soil batches 1, 2 and 4 were stored at −80°C, to analyse the BX concentrations following the sample processing and analysis described in Gfeller *et al*. (2023b). Briefly, BXs were extracted with acidified methanol (70% methanol, 0.1% formic acid) and analysed in an Acquity UHPLC system coupled to a QTOF mass spectrometer without further concentration of samples. Pure BX compounds as described in Gfeller *et al*. (2023b) were used to determine absolute quantities via calibration curves.

### Microbiota profiling

For microbiota profiling, we analysed the bacterial and fungal communities of roots grown in native and sterilised soils along with native soil samples (Experiment III). Sampling, DNA extraction, PCR, library preparation, sequencing and the bioinformatic analysis are detailed in the Methods S3. The microbiota profiles were analysed in R v.4.3.0 (R Core Team, 2017). We first removed reads assigned to Eukarya, Archaea, Cyanobacteria or Mitochondria from the bacterial amplicon sequence variant (ASV) count table. Due to low read numbers, we removed 11 samples from the ITS library (< 900 reads). We normalised both libraries with total sum scaling and did not rarefy the libraries, as the number of reads did not differ between sample groups (Weiss *et al*., 2015). We ran data ordinations based on Bray–Curtis dissimilarity matrices (Bray & Curtis, 1957) using the package phyloseq v.1.44.0 (McMurdie & Holmes, 2013) and visualised them with unconstrained principal coordinate analysis. We performed permutational multivariate analysis of variance (PERMANOVA) with 99′999 permutations with the adonis function from the vegan package to partition and quantify effects of our experimental factors. To determine differentially abundant ASVs, we used four different identification methods, implemented in the packages ALDEx2 v.1.34.0 (Fernandes *et al*., 2013, 2014), Maaslin2 v.1.14.1 (Mallick *et al*., 2021), metagenomeSeq v.1.43.0 (Paulson *et al*., 2013) and ANCOMBC v.2.2.2 (Lin & Peddada, 2020). We defined an ASV to be differently abundant if it was detected (adjusted *P*‐value < 0.05 using the Benjamini–Hochberg method) by at least three of the four methods. Data and codes are made public (see Data availability).

### Transcriptome analysis

Arabidopsis root and shoot sample materials were collected for the RNA‐sequencing (RNAseq) analysis of 7‐wk‐old plants from Experiment V.II. Whole shoots were directly frozen in liquid nitrogen, and the whole roots were collected and removed from all loosely attached soil before freezing. The RNA was extracted with the NucleoSpin RNA kit (Macherey‐Nagel, Düren, Germany) and quantified via Fluorometry. Next, RNA quality was checked with TapeStation and prepared as a library with 200 ng total RNA input using polyA enrichment with the TruSeq Stranded mRNA kit (Illumina, SanDiego, CA, USA). The library was sequenced with a 2× 51‐bp paired‐end protocol with Illumina NovaSeq6000 (Illumina) at the Genomics Facility Basel (https://bsse.ethz.ch/genomicsbasel).

After quality control, reads were aligned to the Arabidopsis TAIR10 reference genome and read count was performed using STAR 2.7.9a (Dobin *et al*., 2013). All raw data processing was performed at sciCORE (http://scicore.unibas.ch/) scientific computing core facility at University of Basel. Downstream analyses were performed with R 4.3.0 using DESeq2 v.1.42.1 (Love *et al*., 2014) for differential gene expression analysis. We considered genes to be differentially expressed if the adjusted *P*‐value (*P*_adj_) < 0.05 and | log_2_ fold change | > 1. For the analysis of co‐expressed genes, we included genes with *P*_adj_ < 0.05 using the package coseq v.1.24.0 (Rau & Maugis‐Rabusseau, 2018). Models were rerun 20 times to determine the optimal number of clusters. Genes with conditional cluster membership probabilities ≥ 0.95 and *P*_adj_ < 0.01 were then used for clusterwise gene ontology (GO) enrichment analysis using hypergeometric testing, as implemented in the package clusterProfiler v.4.10.0 (Wu *et al*., 2021). Data and code are made public (see Data availability).

### General statistical analyses

All statistical analyses were executed in R v.4.3.0 (R Core Team, 2017) within Rstudio v.2023 (Posit team, 2023). Rosette area or biomass data were statistically compared between two groups using two‐sided *t*‐tests, and correlations were assessed with Pearson correlation tests. Analysis of variance test was used to compare more than one group. Data and codes are made public (see Data availability).

## Results

### Arabidopsis growth is enhanced on BX‐conditioned soil

**Fig. 1:**
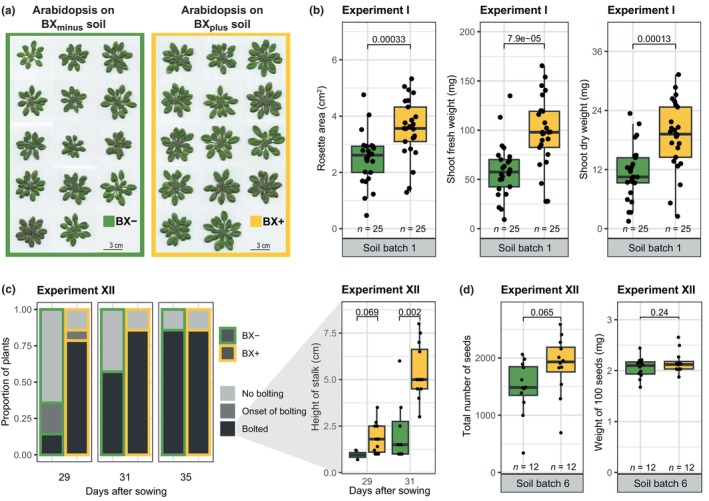
*Arabidopsis thaliana* (Arabidopsis) establishes larger rosettes and is developmentally more advanced when grown on BX_plus_ compared to BX_minus_ soil. (a) Harvested and scanned shoots of Arabidopsis Col‐0 plants grown on control BX_minus_ or BX_plus_ soil. Photographs are from Experiment IV (see Supporting Information Fig. S1d for rosette area data). (b) Shoot growth measurements of Arabidopsis grown for 7 wk on BX_plus_ and BX_minus_ soils. From left to right, the rosette area, shoot fresh weight and shoot dry weight are shown. (c) Feedbacks on flowering were recorded by scoring the fractions of plants that were at the onset of bolting and bolted plants (left) and the heights of the developed flower stalks of bolted plants (right). Plant bolting was recorded at three time points (indicated at the bottom by the number of days after sowing). The height of the flower stalks of bolted plants was recorded at two time points, and the *P*‐values of two‐sided Student's *t*‐tests are shown. (d) Measurements of seeds harvested from Arabidopsis plants that were grown on BX_minus_ and BX_plus_ soils. The total number of seeds of single plants were counted (left) and the weight of 100 seeds (right; calculated based on the total weight of all seeds of a plant, Fig. S1h) are shown. All boxplots in this figure report the median (horizontal line), and the 25^th^ and 75^th^ quartiles (bottom and top edges of the box, respectively). Lower and upper whiskers extend to 1.5× the interquartile range from the bottom and top of the box, respectively. Individual data points are also plotted, representing biological replicates. The boxplot graphs further indicate the *P*‐values of two‐sided Student's *t*‐tests on top, and the replicate numbers (*n*) and the soil batch number of the soil conditioning event at the bottom. In (b)–(d), the name of the experiment from which the data is shown is indicated on top of each panel. The purpose and set‐up of all Arabidopsis experiments on soil are also detailed in Table S3.

In search of a model system to investigate microbiome feedbacks, we tested the Arabidopsis accession Col‐0 and asked if BX‐conditioned soils would lead to differences in growth. We exposed Arabidopsis plants to contrasting soil microbiomes by growing them individually in pots either containing control ‘BX_minus_ soil’, where no BXs were detected, or BX‐conditioned ‘BX_plus_ soil’, where MBOA and AMPO had accumulated (Fig. S1b). We observed that plants developed larger rosettes when grown on BX_plus_ compared to the control BX_minus_ soil (Fig. 1a). This observation was confirmed with the shoot fresh and dry weight and was also reflected using image‐based quantification of the rosette area (Fig. 1b), which correlated strongly with shoot biomass (Fig. S1c), and was therefore employed for the rest of the study. The positive feedback on growth was reproducible with varying setups conducted with independently conditioned soil batches (Fig. S1d, Table S3) and was not affected by the soil storage duration (Fig. S1a). This positive feedback was increasing over time (Fig. S1e), and we also noticed a feedback on development with increasing number of leaves at a late growth stage (Fig. S1f). Furthermore, we also recorded larger rosettes (Fig. S1g) and an earlier transition to flowering when plants were grown under long‐day conditions on BX_plus_ soil (Fig. 1c). This coincided with higher seed yield on BX_plus_ soil (Fig. 1d; also quantified by weight, Fig. S1h), whereas the average seed weight did not differ between the two soils (Fig. 1d). We also investigated if root growth was affected in two experiments and found a trend to higher root biomass on BX_plus_ soil (Fig. S1i), which correlated significantly and positively with rosette area (Fig. S1j). Taken together, Arabidopsis Col‐0 responded with increased growth and faster development on BX_plus_ compared to BX_minus_ soil.

### Arabidopsis growth differences depend on the soil microbiome

**Fig. 2:**
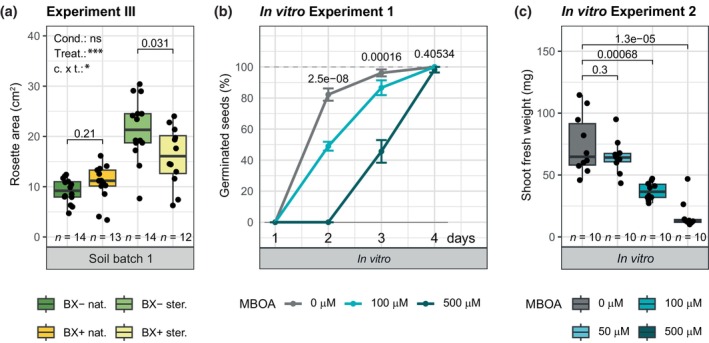
*Arabidopsis thaliana* (Arabidopsis) does not establish larger rosettes on sterilised BX_plus_ compared to sterilised BX_minus_ soil and is sensitive to 6‐methoxybenzoxazolin‐2(3H)‐one (MBOA) *in vitro*. (a) Rosette area of Arabidopsis plants grown on native (‘nat.’) and sterilised (‘ster.’) BX_minus_ and BX_plus_ soils. The soil batch number of the soil conditioning event is indicated at the bottom of the graph. The significance levels of the ANOVA model testing the effect of conditioning (‘cond.’) and sterilisation treatment (‘treat.’) on rosette area are depicted in the left top corner of the graph. (b) Percent of germinated seeds on half‐strength MS plates supplemented with DMSO (‘0 μM’) or increasing amounts of MBOA (dissolved in DMSO), recorded at day 1 to 4 after sowing. The *P*‐values on top of each time point represent the ANOVA statistics testing for differences between the groups. (c) Shoot fresh weight of plants grown on half‐strength MS plates supplemented with DMSO (‘0 μM’) or increasing amounts of MBOA dissolved in DMSO. One data point represents the total shoot fresh weight from five plants grown on one plate. All boxplots shown in this figure report the median (horizontal line), and the 25^th^ and 75^th^ quartiles (bottom and top edges of the box, respectively). Lower and upper whiskers extend to 1.5 × the interquartile range from the bottom and top of the box, respectively. Individual data points are also plotted, representing biological replicates. The boxplot graphs further indicate the *P*‐values of two‐sided Student's *t*‐tests on top and the replicate numbers (*n*) at the bottom. The purpose and set‐up of all Arabidopsis experiments on soil are also detailed in Table S3.

With maize, we previously found that differential soil microbiotas were driving the feedbacks based on a sterilisation experiment (Hu *et al*., 2018). To test this also with Arabidopsis, we sterilised BX_minus_ and BX_plus_ soils using X‐radiation. Compared to the native soils, Arabidopsis grew overall larger on the sterilised soils (Fig. 2a), as sterilisation can enhance organic matter content via lysis of microbial cells (Berns *et al*., 2008). The better growth on native BX_plus_ soil was no longer seen on sterilised soil, confirming that soil biotic factors drive these feedbacks. In contrast to native soils, Arabidopsis plants grew smaller on sterilised BX_plus_ compared to sterilised BX_minus_ soil. This suggested that Arabidopsis growth was reduced by a factor contained in sterilised BX_plus_ soil. The primary candidates for this factor are the BXs, as both MBOA and AMPO levels were not affected by the sterilisation (Fig. S1b). AMPO has been previously demonstrated for its allelopathic properties against Arabidopsis (Venturelli *et al*., 2015), thus we have also tested Arabidopsis’ sensitivity to MBOA. We did this first in sterile settings, as it would be initially the case in sterilised soil.

In agar plates containing MBOA, seed germination was delayed (Fig. 2b) and shoot biomass of 3‐wk‐old plants was reduced starting at 100 μM MBOA (Fig. 2c). Roots were less sensitive, as length (Fig. S2a) and biomass (Fig. S2b) were only affected at 500 μM MBOA. We then tested Arabidopsis sensitivity to MBOA in nonsterile settings. Applying MBOA to unconditioned native soil, we only found a slight reduction in shoot growth at 500 μM MBOA (Fig. S2c).

Thus, although Arabidopsis was sensitive to MBOA *in vitro*, only minor allelopathic effects and at higher MBOA concentrations were observed for Arabidopsis grown in natural soil. To conclude on the sterilisation experiment (Fig. 2a), the biotic soil properties shaped by BX‐conditioning were needed for the positive growth of Arabidopsis, highlighting that BX‐mediated feedbacks on Arabidopsis represent *microbiome feedbacks*.

### The Arabidopsis root microbiota is altered on BX‐conditioned soil

Our previous work had also shown that differential microbiotas assembled on roots of maize plants when grown on BX_minus_ compared to BX_plus_ soils (Hu *et al*., 2018). To test for this with Arabidopsis, we profiled bacterial and fungal communities on roots and soil collected from the sterilisation experiment (Experiment III). We have also profiled the root microbiotas of plants grown in the initially sterilised soils, as the roots will become re‐colonised by microbes during the course of the experiment in the non‐sterile growth chamber.

**Fig. 3:**
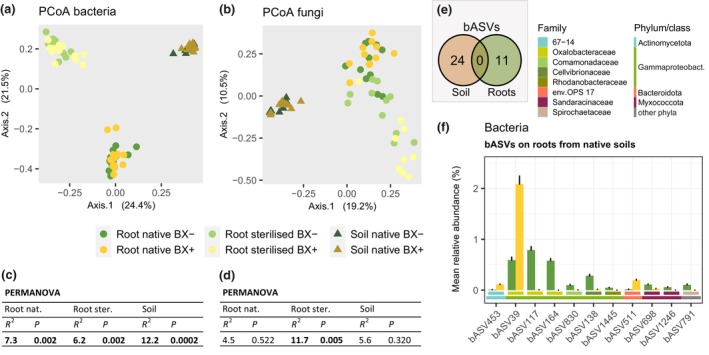
The microbiota of soil and *Arabidopsis thaliana* (Arabidopsis) roots differ between BX_plus_ and BX_minus_ soil. Principle coordinate analysis (PCoA) of (a) bacterial and (b) fungal community profiles from BX_minus_ and BX_plus_ soils and Arabidopsis roots grown in native or initially sterilised BX_minus_ and BX_plus_ soils. Replicate numbers are *n* = 10–14 for bacterial and *n* = 9–11 for fungal samples. Permutational multivariate analysis of variance tables on Bray‐Curtis dissimilarities of (c) bacterial and (d) fungal community profiles report the effect of the conditioning variable on community composition for each of the three sample groups, with *R* ^2^ and *P*‐values displayed for the conditioning variable. Statistically significant differences are shown in bold. (e) Overlap of bacterial amplicon sequence variants (bASVs) that differed in abundance due to conditioning between native soil and roots from native soils. (f) Abundance and taxonomy of differentially abundant bASVs on Arabidopsis roots from native BX_minus_ and BX_plus_ soils. Error bars represent the SE of the mean. The coloured horizontal bars below the bargraphs indicate the bacterial family and phylum/class information (for the Pseudomonadota phylum, the class information is given, i.e. Gammaproteobacteria). The reported bASVs were identified as differentially abundant by at least three of four statistical tools used, namely ALDEx2, Maaslin2, metagenomeSeq and ANCOMBC (see the Materials and Methods section for more details).

First, we examined the communities for compositional differences between sample groups. For bacteria, ordination analysis revealed a clear separation of the communities between soil and between the two groups of roots with strongly differing communities from plants grown in native or initially sterilised soils (Fig. 3a). For the fungi, soil and root communities were also separated, but the separation between plants from native or sterilised soil was less marked than for the bacteria (Fig. 3b). Next, we quantified the effect sizes of sample groups and of the soil conditioning based on the *R* ^2^ values of PERMANOVA. Because of the overly large variation among the different sample types (bacteria 23.4%; fungi 17.8%) relative to the conditioning effects (bacteria 1.5%; fungi 1.9%; Dataset S1), we tested the significance of the effect sizes due to conditioning within each sample group separately. We found significantly different bacterial communities between BX_minus_ and BX_plus_ in all sample groups. BX‐conditioning explained 12.2% of the bacterial community variation in soil, 7.3% on roots from native soils and 6.2% on roots from sterilised soils (Fig. 3c). The fungal communities only differed on roots from sterilised soil, where the BX‐conditioning explained 11.7% of the fungal community variation (Fig. 3d). Together with the soil BX analysis (Fig. S1b), the microbiota profiling points to the conclusion that BXs in soil cause a differentiation of the soil and root microbiotas. This further suggests that these differential microbiotas – particularly the bacteria – may drive the differential growth of Arabidopsis.

Next, we performed an in‐depth microbiota analysis, which is documented in the Dataset S1. In essence, with robust statistic support (adjusted *P*‐value < 0.05) from at least three of the four used statistical tools, we detected in soil 24 bacterial sequences (named *b*acterial *A*mplicon *S*equence *V*ariant*s*, bASVs) whose abundances were dependent on BX‐conditioning (Fig. 3e, Table S4). BX_plus_ soil was mainly enriched with seven abundant (> 0.1% relative abundance) Sphingomonadaceae, two Gammaproteobacteria, a Chloroflexota and four Acidobacteriota bASVs, whereas bASVs assigned to Actinomycetota, Gammaproteobacteria and other taxonomic groups were depleted (Fig. S3). In roots, other 11 bASVs differed between the microbiotas of plants grown in native BX_minus_ vs BX_plus_ soil (Fig. 3e, Table S5). Most apparent was the strong enrichment of the abundant bASV39 (genus Massilia, family Oxalobacteraceae) on roots of plants grown in BX_plus_ soil, whereas other ASVs of this family or of the same class were depleted (Fig. 3f). To summarise, consistent with maize, Arabidopsis had different bacterial communities on the roots when grown on the two conditioned soils.

### Arabidopsis alters expression of development‐ and defence‐related genes on the BX‐conditioned soil

To obtain insights into how Arabidopsis achieved better growth on BX_plus_ soil, we performed a transcriptome analysis of shoot and root samples from plants grown for 7 wk on BX_minus_ and BX_plus_ soils (Fig. S1d, Experiment V.II). The detailed RNAseq analysis is documented in the Dataset S2. Comparing plants grown on control BX_minus_ vs BX_plus_ soil, we found 1'959 and 1'839 differentially expressed genes in shoots (Table S6) and roots (Table S7), respectively (Criteria: adjusted *P*‐value, *P*_adj_ < 0.05; log_2_ fold change, | log_2_FC | > 1). The subsequent co‐expression and GO enrichment analysis (criteria: membership probabilities ≥ 0.95 and *P*_adj_ < 0.01) revealed that soil conditioning affected many and diverse biological processes both in shoots (Fig. S4a, Table S8) and roots (Fig. S4b, Table S9). Because many enriched GO terms were supported by rather few genes, we focused on the top two in each co‐expression cluster.

**Fig. 4:**
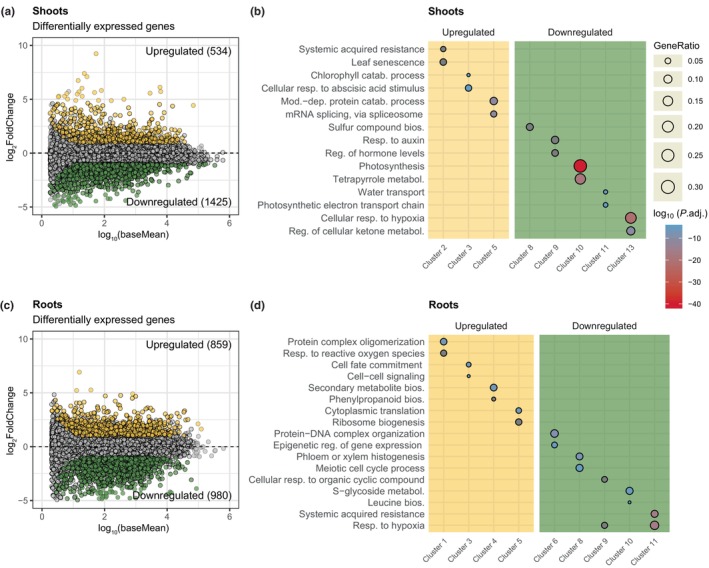
The transcriptome of *Arabidopsis thaliana* shoots and roots differs between BX_plus_ and BX_minus_ soil. (a) MA plot of genes expressed in shoots, plotted by their expression level on the *x*‐axis and by the log_2_ fold change (log_2_FC) between BX_plus_ and BX_minus_ soils on the y‐axis. Dots in yellow represent genes that were upregulated in shoots grown on BX_plus_ soil compared to BX_minus_ soil, whereas green dots represent downregulated genes (criteria: adjusted *P*‐value (*P*_adj_) < 0.05, |log_2_FC| > 1), and grey dots represent genes that were not differentially regulated. The numbers of up‐ and downregulated genes are reported on the top‐right and bottom‐right in the MA plot, respectively. (b) Representation of the top two gene ontology (GO) terms in each co‐expression cluster for up‐ and downregulated genes. The top GO terms per cluster were defined by the highest gene ratios, that is, the fraction of differentially expressed genes of a GO term. Colour and size represent the adjusted *P*‐values for enrichment of the GO term and its gene ratio, respectively. Some GO terms were shortened due to space (see Supporting Information Tables S8 and S9 for full GO terms). (c) and (d) report the same information as (a) and (b), but for the roots.

In shoots, 534 genes were upregulated and 1'425 downregulated on BX_plus_ compared to the control BX_minus_ soil (Fig. 4a, Table S6). The main upregulated processes involved systemic acquired resistance (SAR), leaf senescence and chlorophyll and protein catabolism as well as response to abscisic acid (Fig. 4b). Although Tables S8 and S9 list all genes and their links to GO term numbers, we report the annotation and statistics of exemplary genes for the mentioned GO terms in Table S10. For instance, we noticed that the defence marker genes *PR5* and *PR2* were upregulated, as was *PR1*, the classical marker gene for SA defences and SAR (Ryals *et al*., 1996; van Loon *et al*., 2006; Table S6).

Downregulated processes included primarily photosynthesis, metabolisation of tetrapyrroles, responses to auxin and genes related to decreased oxygen and hypoxia (Fig. 4b, Table S8). Taken together, the shoot transcriptomes revealed that plants growing on BX_plus_ soil were developmentally more advanced, that is, towards senescence and with reduced photosynthesis, while having activated SA defences.

In roots, 859 and 980 genes were up‐ and downregulated , respectively (Fig. 4c, Table S7). The main upregulated processes included protein complex oligomerization, response to reactive oxygen species, cell fate commitment and signalling, secondary metabolite and phenylpropanoid biosynthesis (Fig. 4d, Table S9). For the latter, several well‐known genes involved in the biosynthesis of suberin (Serra & Geldner, 2022), such as *CYP86A1*, *GPAT5*, *FAR4*, and *ASFT*, were significantly upregulated in BX_plus_ grown plants (Table S10). The main downregulated processes were related to responses to low oxygen, S‐glycoside metabolism, various developmental processes, and related to SAR, where the differences in SAR processes were mainly driven by two BX_minus_ samples with high expression of SAR‐related genes (Figs 4d, S4b, Table S9). In summary, the root transcriptomes revealed that plants growing on BX_plus_ soil altered root developmental processes and had downregulated defences. Overall, we concluded that BX‐conditioning changed defence processes in both shoots and roots and also altered growth and development related processes.

### Arabidopsis is more resistant to Botrytis and is primed for SA‐induced defences on the BX‐conditioned soil

**Fig. 5:**
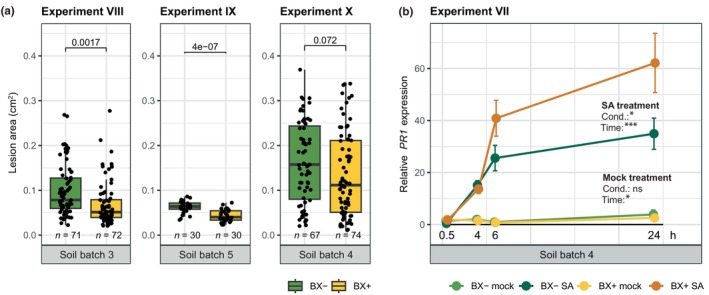
*Arabidopsis thaliana* (Arabidopsis) is more resistant to *Botrytis cinerea* (Botrytis) and expresses more *PR1* after salicylic acid (SA) treatment on BX_plus_ compared to BX_minus_ soil. (a) Quantification of Botrytis infections based on the area of necrotic lesions on Arabidopsis leaves. Three leaves per plant were infected at 5 wk for Experiment IX and at 6 wk for experiments VIII and X. Boxplots report the median (horizontal line), and the 25^th^ and 75^th^ quartiles (bottom and top edges of the box, respectively). Lower and upper whiskers extend to 1.5 × the interquartile range from the bottom and top of the box, respectively. Individual data points are also plotted, representing lesion area data of individual leaves. The boxplot graphs further indicate the *P*‐values of two‐sided student's *t*‐tests on top, and the replicate numbers (*n*; number of infected leaves) and the soil batch number of the soil conditioning event at the bottom. (b) Expression of *PR1* relative to the reference gene *PP2A* in mock‐treated and SA‐treated shoots after 0.5, 4, 6 and 24 h, in plants grown on BX_plus_ and BX_minus_ soil. Error bars represent the SE of the mean, where *n* = 4 for each group. The significance levels of ANOVA testing the effects of the conditioning (cond.) and time after treatment on *PR1* expression are depicted on the right side of the graph, for both treatments separately. The purpose and set‐up of all Arabidopsis experiments on soil are also detailed in Supporting Information Table S3.

The transcriptome analysis revealed a signature of enhanced defences in shoots of plants grown on BX_plus_ soil (Fig. 4b). To test actual disease resistance of Arabidopsis, we infected leaves with the necrotrophic fungus Botrytis and quantified the area of the developing lesions. We recorded slightly smaller lesions on plants grown on BX_plus_ compared to BX_minus_ soil in three independent experiments performed on three different soil batches (Fig. 5a). Hence, Arabidopsis plants grown on BX_plus_ soil were more resistant to the pathogenic fungus.

It is formally possible that the enhanced resistance to Botrytis was not triggered by the microbiota but chemically, that is by the BX levels in soil (Fig. S1b). Thus, we tested whether MBOA additions would affect Arabidopsis' resistance to the fungus, both in sterile and soil conditions. In a sterile glass jar system (McLaughlin *et al*., 2023), exposure of the plants to 50 μM MBOA in the growth medium decreased not only shoot biomass but also increased lesion area, indicating decreased resistance to Botrytis (Fig. S5a). In soil, however, exposures of up to 500 μM MBOA did not change resistance to Botrytis, whether added at sowing or only 3 d before infection (Fig. S5b). Therefore, we concluded that MBOA could not trigger Arabidopsis' resistance to Botrytis, suggesting that residual BXs in soil are unlikely to induce the plant defences directly.

In the shoots, the BX_plus_ soil and/or root microbiota triggered an enhanced expression of genes involved in SAR (Fig. 4b). The phenomenon SAR is mediated by the phytohormone SA and establishes after survival of a primary pathogen infection through primed defences (Zeier, 2021). Priming refers to stronger and/or faster defence gene induction upon a second challenge (Martinez‐Medina *et al*., 2016). To test whether Arabidopsis' defences were primed when plants were grown on BX_plus_ compared with BX_minus_ soil, we treated the shoots of plants grown on both soils with SA (as secondary challenge) and recorded *PR1* expression levels over time. While *PR1* was not induced in mock‐treated plants from both soils, following the SA treatment we found increased *PR1* expression in plants grown on BX_plus_ compared with BX_minus_ soil (Fig. 5b). Combining these findings allowed us to conclude that the BX_plus_ soil and/or root microbiota primed the SA‐dependent defences, which is consistent with Arabidopsis' enhanced resistance to Botrytis when grown on BX_plus_ compared to BX_minus_ soil.

## Discussion

**Fig. 6:**
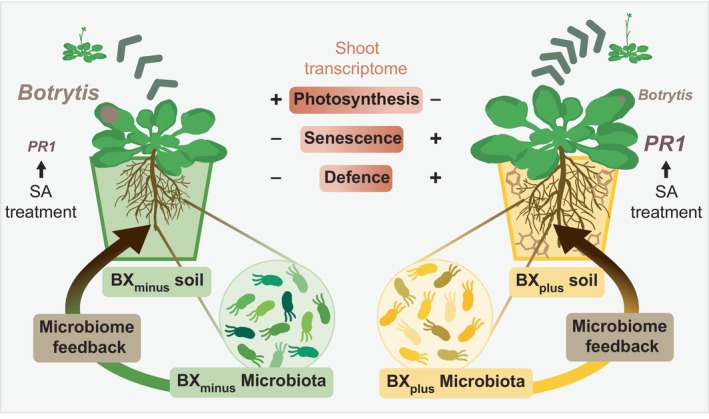
Summarised effects of benzoxazinoid (BX)‐conditioned soil microbiomes on *Arabidopsis thaliana* (Arabidopsis). Graphical summary of Arabidopsis responses when grown on BX_plus_ soil compared to the control BX_minus_ soil. BX_plus_ and BX_minus_ soils are physico‐chemically the same but differ in previous conditioning by either wild‐type BX exuding *Zea mays* plants or non‐BX‐exuding mutant plants, respectively. The exudation of BXs to soil (indicated with chemical structures in the BX_plus_ pot) affects the soil and root microbiota compositions. Arabidopsis grown on BX_plus_ soil increased root and shoot growth and accelerated development (indicated with the green arrows and icons of bolted plants). This enhanced development was also supported by the shoot transcriptome analysis (orange boxes with colour gradient), which revealed that plants on BX_plus_ soil had reduced photosynthesis and increased senescence processes. Further, the shoot transcriptome analysis also revealed that Arabidopsis on BX_plus_ soil had enhanced defences. This was supported by the enhanced resistance of Arabidopsis to *Botrytis cinerea* (lesion sizes are indicated), and enhanced expression of the defence gene *PR1* after treatment with salicylic acid (SA), when Arabidopsis was grown on BX_plus_ soil compared to when grown on BX_minus_ soil. The enhanced growth on BX_plus_ soil was lost after soil sterilisation, revealing that the differential microbiotas shape the feedbacks, which we therefore refer to as a microbiome feedback (depicted by the arrows leading from the microbiota back to the plant).

Plant–soil feedbacks (PSFs) describe the performance of a new plant generation to the soil legacy caused by the previous plant generation. We previously reported PSFs where maize growth leads to BX‐dependent alterations of the soil microbiota, which then drives the feedbacks of next generation maize or wheat plants (Hu *et al*., 2018; Cadot *et al*., 2021). Mechanistically, how plants respond to altered soil microbiotas and in turn modulate their own performance is not well understood so far. In this study, we established a model system with Arabidopsis to investigate its feedback responses to differential microbiotas in soil, which were established by differential BX exudation of maize. With Fig. 6 we summarise our work and discuss below the implications of our findings in the broader context of how plants mediate microbiome feedbacks.

In summary, we found that Arabidopsis responded with increased shoot and root growth, faster development, and also SA‐dependent priming and enhanced resistance to shoot infections with the pathogen Botrytis when grown on BX_plus_ soil (Fig. 6). These feedback phenotypes were consistent with the transcriptome analysis revealing also that plants were developmentally more advanced (earlier senescence, reduced photosynthesis) while having enhanced defences in shoots when grown on BX_plus_ soil. We found differential root microbiotas when Arabidopsis was grown on the two soils and that the positive growth feedback was lost after soil sterilisation. Both observations are consistent with earlier work on maize (Hu *et al*., 2018), corroborating the conclusion that the feedbacks are driven by soil microbes. The discovery of the plant immune receptor Mediator of Microbiome Feedback 1 (MMF1) in Arabidopsis feedbacks to BX_plus_ soil (Janse Van Rensburg *et al*., 2025) implies recognition of microbial signals from soil, providing additional evidence that the feedbacks are caused by the soil microbiota. Hence, BX‐mediated feedbacks on Arabidopsis are *microbiome feedbacks* (Fig. 6). The occurrence of such feedbacks on Arabidopsis with its many available research resources makes this a good model system to study the molecular mechanisms of microbiome feedbacks.

### Arabidopsis is developmentally advanced when grown on the BX‐conditioned soil microbiome

Next to the enhanced vegetative growth on BX_plus_ soil, Arabidopsis also had more leaves at a late growth stage (Fig. S1f), showed earlier bolting (Fig. 1c), and also produced more seeds (Fig. 1d). Our transcriptome analysis of 7‐wk‐old plants intriguingly did not reveal typical growth‐related processes to be differentially regulated, but we did find indications that plant development could be affected: photosynthesis‐related genes were downregulated and senescence processes upregulated in plants grown on BX_plus_ soil (Fig. 4b). Also in roots, there was evidence for developmental advance: A few genes related to phenylpropanoid biosynthesis – particularly suberin – were upregulated (Fig. 4d, Table S10), a process typical for more mature roots because of secondary growth and production of periderm. Collectively, we concluded that plants grown on BX_plus_ soil were developmentally more advanced. An alternative explanation for this developmental advance could be differences in germination, as previous work with wheat showed better germination on BX_plus_ compared to BX_minus_ soil (Gfeller *et al*., 2023b). Future experiments are needed to investigate germination effects and to disentangle them from developmental processes involved in BX‐mediated microbiome feedbacks.

### Microbial and chemical drivers for feedbacks on Arabidopsis

From the soil microbiota, a potential driver for the Arabidopsis growth feedbacks could be Sphingomonadaceae bacteria that were enriched in BX_plus_ soil (Fig. S3). Members of this bacterial family can metabolise MBOA into AMPO (Thoenen *et al*., 2024). This potentially affects the microbial composition in soil, as both MBOA and AMPO can have antimicrobial properties but against different bacterial groups (Thoenen *et al*., 2023). Thus, Sphingomonadaceae could indirectly affect plant growth by affecting the abundance of growth promoting bacteria via conversion of MBOA to AMPO.

Furthermore, we identified a strongly enriched and abundant Massilia bacterium (bASV39) on roots of Arabidopsis plants grown in BX_plus_ soil (Fig. 3f), which might be coupled to the enhanced growth and earlier transition to flowering. Massilia bacteria are recurrent members of plant root microbiomes including Arabidopsis, and they are often associated with beneficial traits (Beilsmith *et al*., 2021; Yu *et al*., 2021; Li *et al*., 2025). Massilia were also recently reported to drive growth promotion of maize, and that this beneficial trait depended on flowering time (Wang *et al*., 2024). Hence, Oxalobacteraceae and particularly Massilia clearly warrant future functional investigations using synthetic microbial communities or single strains of this genus for their involvement in BX‐mediated microbiome feedbacks on Arabidopsis.

The finding that this positive growth feedback was reversed on sterilised soils (Fig. 2a) requires a detailed reflection. While the soil microbiota was eliminated by sterilisation of the soil, the BX contents stayed intact (Fig. S1b), representing the main chemical difference between BX_minus_ and BX_plus_ soil (Hu *et al*., 2018; Gfeller *et al*., 2023b). Intuitively, given the allelopathic nature of BXs, this negative feedback on sterilised soil might be due to the residual BXs in soil. We show here that Arabidopsis root and shoot growth can be inhibited by MBOA at concentrations in the μM to mM range both in sterile systems and on natural soil (Figs 2b,c, S2). Yet, concentrations measured in soil were lower and in the nM range (Fig. S1b). This discrepancy in concentrations likely stems from quantifying BXs averaged across a whole soil sample, while *in situ* they occur in concentrated hotspots close to root tips where they are predominantly released (Bilyera *et al*., 2021). Therefore, allelopathic effects of BXs like in sterilised BX_plus_ soil seem plausible, even though the compounds in soil are quantified at lower concentrations compared to their tested *in vitro* activity.

Alternatively, the negative growth feedbacks on sterilised soils might also be mediated by the newly established microbiota. In support of this hypothesis, the bacterial communities on Arabidopsis roots from sterilised soils also differed by BX‐conditioning (Fig. 3c), and they might explain the differences in shoot growth. This also implies that the residual BXs in sterilised BX_plus_ soil were strong enough determinants to differentiate the freshly assembled root communities via re‐colonisation of sterilised soil. Thus, we concluded that the chemical component of BX‐mediated microbiome feedbacks on Arabidopsis plays a more indirect role, and that these feedbacks are primarily driven through interactions with the soil microbiota that then affect plant growth.

### Arabidopsis defence is enhanced when grown on the BX‐conditioned soil microbiome

The increased resistance of Arabidopsis to Botrytis when grown on BX_plus_ soil (Fig. 5a) could have different causes. First, migration of disease suppressive bacteria from soil to leaves could directly inhibit the pathogen, as it was recently shown for soil conditioned by downy mildew infected Arabidopsis (Goossens *et al*., 2023). Second, the advanced plant development (Fig. 1c) could also be involved, as ageing is generally associated with enhanced resistance (Hu & Yang, 2019). Dissecting the mechanistic linkage(s) between advanced development and enhanced defence will require further investigations as contrasting molecular responses or effects on resistance were found. In Arabidopsis, for instance, while older plants with lower expression of *PR1* and SA‐related genes were more resistant to *Pseudomonas syringae*, they were not to Botrytis (Rusterucci *et al*., 2005; Xu *et al*., 2018). In contrast to such direct age‐related links to pathogen resistance, the enhanced resistance to Botrytis may have been triggered by the BX_plus_ soil microbiome, which prepared the plants to respond with more enhanced expression of defences like the SA‐related genes. This latter, referred to as defence priming, could be a third possible cause. Priming is a state of alert where plants can mount a faster and stronger defence response than un‐primed plants (Martinez‐Medina *et al*., 2016; Karasov *et al*., 2017; Harris *et al*., 2023). Indeed, the increased *PR1* expression after SA treatment in plants grown on BX_plus_ soil (Fig. 5b) implied that these plants were primed. Furthermore, priming usually exerts no or only little costs compared to constitutive defences, and therefore, it could serve as the explanatory mechanism for the lack of a growth‐defence trade‐off, which was observed with the improved growth and the enhanced resistance of Arabidopsis on BX_plus_ soil.

### Does the BX‐conditioned soil microbiome prime Arabidopsis for enhanced defences?

To establish defence priming in plants, a preceding priming stimulus is needed, such as the presence of a chemical (Mauch‐Mani *et al*., 2017). We have tested whether the MBOA contained in BX_plus_ soil could directly induce resistance to Botrytis by adding MBOA to plants and found that this was not the case (Fig. S5). Thus, we concluded that the BX_plus_ microbiota would be responsible for the priming of *PR1* and for the increased resistance to Botrytis. Furthermore, priming established by root microbes might also explain the reduced expression of defence genes in the roots of BX_plus_ grown plants (Fig. S4b), as plant immunity was also suppressed by the colonisation of the resistance‐inducing bacterium *Pseudomonas simiae* WCS417 (Millet *et al*., 2010; Stringlis *et al*., 2018a). This reduced expression of defence genes in the roots will require further research, not only because two replicates in our transcriptome analysis strongly impacted the average response (Fig. S4b) but more generally, to corroborate whether root immunity is downregulated during priming for systemic resistance.

Mechanistically, this priming state can be mediated by the jasmonic acid (JA) pathway, which is often the case when priming is established by root colonisation of beneficial microbes, or via the SA‐pathway, such as after a defended pathogen attack on the leaves or also after root colonisation by beneficial microbes (Ross, 1961; Pieterse *et al*., 1996, 2014; Zeier, 2021). In our system, we find evidence that the SA pathway was activated in shoots of BX_plus_ grown plants, and therefore, we suggest that SA‐dependent priming was likely involved in the enhanced resistance to Botrytis. Although resistance to necrotrophic pathogens like Botrytis is generally mediated by the JA pathway (Shigenaga & Argueso, 2016), the SA pathway can still be involved, as, for example, root colonisation by a beneficial fungus induced expression of *PR* genes in shoots, which could prime Arabidopsis' resistance to Botrytis (Mathys *et al*., 2012). As there is also emerging evidence for enhanced resistance elicited by whole soil microbiotas (Badri *et al*., 2013; Hubbard *et al*., 2019; Pineda *et al*., 2020), it appears plausible that the BX_plus_ microbiota primed Arabidopsis for SA‐related defences, which resulted in the increased resistance to Botrytis.

### Relevance of Arabidopsis responses to the BX‐conditioned soil microbiome

With our findings on Arabidopsis, we extend the effects of BX‐mediated microbiome feedbacks on plant performance from BX‐producing species to plants that do not produce BXs themselves but are still affected by these feedbacks. Notably, with Arabidopsis we report simultaneously enhanced growth and resistance, while often only one or the other was reported for maize or wheat (Hu *et al*., 2018; Cadot *et al*., 2021). Thus, the potential of these feedbacks to elicit positive but not negative feedbacks in more distant plants such as Brassicaceae (plant family of Arabidopsis) should be further explored in agricultural settings. For example, testing *Brassica napus* (rapeseed) in crop rotation following maize could elucidate the translatability of the microbial feedbacks studied with the Arabidopsis model system established in this study.

Furthermore, defence signatures in response to the BX_plus_ soil also present a possible target for translation. In Arabidopsis, we found SA‐dependent defences to be upregulated in the shoots grown on BX_plus_ soil, whereas in maize, better resistance to herbivores coincided with higher levels of SA and JA, and enhanced expression of JA‐related defence genes in the shoots of unchallenged BX_plus_ grown plants (Hu *et al*., 2018; Cadot *et al*., 2021). Thus, while the type of hormonal signalling differs between maize and Arabidopsis, both plant species share the commonality of enhanced activation of defence genes when mediating microbiome feedbacks. Further, this shared defence gene activation appears as a possible molecular target for translation. Based on comparative analyses of growth‐regulatory mechanisms, Inzé and Nelissen proposed that the particular focus on gene space conservation will enhance the translatability of genetic networks from model to crop species (Inzé & Nelissen, 2022). In the context of the BX‐mediated microbiome feedbacks, thus, future work targeting for example conserved transcription factor families that could mediate defence gene activation both in Arabidopsis and maize seems a logical step forward.

### Conclusion

The potential to use the beneficial functions of the soil or root microbiome as a more sustainable way to improve plant growth and health in agriculture is well recognised (French *et al*., 2021; Santos & Olivares, 2021). However, the mechanisms by which plants perceive and respond to specific soil microbiomes are currently not well understood (Janse van Rensburg *et al*., 2024). Our study provides a first step in this direction, as we show that Arabidopsis can respond with both improved growth and enhanced resistance to a specific soil microbiome. The model system with BX‐conditioned soil microbiomes and their feedbacks on Arabidopsis will allow us to further study the mechanisms by which Arabidopsis can perceive and mediate microbiome feedbacks, and especially to identify the plant genes that are important for this process. Possibly, such knowledge can help to improve breeding programs so that crops can take full advantage of their microbiome.

## Competing interests

None declared.

## Author contributions

Katja Stengele, LT and Klaus Schlaeppi planned and designed the research. Katja Stengele, HJvR, LT and VD performed experiments. Katja Stengele and LS analysed the data. Katja Stengele and Klaus Schlaeppi wrote the manuscript.

## Disclaimer

The New Phytologist Foundation remains neutral with regard to jurisdictional claims in maps and in any institutional affiliations.

## Supporting information


**Dataset S1** Microbiota analysis.

**Dataset S2** Transcriptome analysis.

**Fig. S1** Benzoxazinoid concentrations in soils and *Arabidopsis thaliana* growth and development measurements on different soil batches.
**Fig. S2** *Arabidopsis thaliana* root growth on MBOA supplemented agar plates and rosette area on soil supplemented with MBOA.
**Fig. S3** Differentially abundant bacterial amplicon sequence variants between native BX_plus_ and native BX_minus_ soil.
**Fig. S4** Co‐expression analysis of genes expressed in *Arabidopsis thaliana* shoots and roots grown on BX_plus_ compared to BX_minus_ soil.
**Fig. S5** MBOA does not enhance resistance of *Arabidopsis thaliana* to *Botrytis*.
**Methods S1** Feedback experiments on soil.
**Methods S2** *In vitro* experiments.
**Methods S3** Microbiota profiling.
**Table S1** Setup of the different conditioning experiments.
**Table S2** Nutrient concentration of soil batches.
**Table S3** Setup of the different *Arabidopsis thaliana* experiments on soil.
**Table S4** List of differently abundant bacterial amplicon sequence variants in soil.
**Table S5** List of differently abundant bacterial amplicon sequence variants on roots of *Arabidopsis thaliana*.

**Table S6** Shoot transcriptome.
**Table S7** Root transcriptome.
**Table S8** Shoot co‐expression analysis.
**Table S9** Root co‐expression analysis.
**Table S10** List of exemplary genes from the transcriptome analysis.
Please note: Wiley is not responsible for the content or functionality of any Supporting Information supplied by the authors. Any queries (other than missing material) should be directed to the *New Phytologist* Central Office.

## Data Availability

The source data on plant biomass and soil physicochemical analyses is made publicly available on https://github.com/PMI‐Basel/Stengele_et_al_At_BX‐feedbacks. The raw sequencing data are stored at the European Nucleotide Archive (http://www.ebi.ac.uk/ena). The RNAseq data are stored under project number PRJEB80860, and the microbiota sequencing data are available under the project number PRJEB59165 (The 16S rRNA and ITS gene libraries of this study were sequenced in the same MiSeq run together with a library of an already published project (Gfeller *et al*., 2023a)). All bioinformatic code including information on barcodes, primers and sample assignments is provided on GitHub. On GitHub we have also deposited all code used for statistical analysis and graphing of all figures.
