# Supplementary material for: Benzoxazinoid‐mediated microbiome feedbacks enhance Arabidopsis growth and defence
Source: New Phytol. 2026 Mar 17;250(5):3334–48. doi: 10.1111/nph.71098 (PMC13150306; doi:10.1111/nph.71098)
Supplement: Supplementary file 1 — Dataset S1 Microbiota analysis. [file NPH-250-3334-s001.pdf]

Stengele et al Dataset S1: Microbiota analysis

Katja Stengele

2025-12-23

Contents

1. Background **2**

1.1 Import data files 2

1.1.1 Bacteria 2

1.1.2 Fungi 2

1.2 Colours 3

2. Data normalization **3**

2.1. Bacteria 3

2.2 Fungi 5

3. Microbiota Analysis Results **6**

3.1 Alpha Diversity 6

3.2 Beta diversity 7

3.2.1 PERMANOVA 7

3.2.2 PCoA - unconstrained ordination 8

3.2.3 CAP - constrained ordination 9

3.3 Phyla distribution 11

3.4 Determination of differently abundant ASVs 11

3.4.1 bASVs on roots grown in native soil 11

3.4.2 bASVs on roots grown in sterilized soil 12

3.4.3 bASVs in soil 13

3.4.4 bASVs overlap 14

3.4.5 fASVs on roots grown in native soil 15

3.4.6 fASVs on roots grown in sterilized soil 16

3.4.7 fASVs in soil 16

1. Background

This report contains the microbiota analysis of Experiment III described in the main part of the publication. In brief, we used soil previously conditioned by twelve weeks of maize growth of either wild-type B73 maize (= BX+ soil), or BX-deficient *bx1* mutant in the B73 variety background (= BX- soil). Arabidopsis thaliana Col-0 was grown on the conditioned soil batch 2, where plants were either grown on native conditioned soil (BX+ native or BX- native), or on soil that was sterilized by X-radiation after the conditioning (BX+ sterilized or BX- sterilized). Plants were grown for six weeks before the roots were harvested and washed. We have then sequenced the bacterial and fungal communities of roots from native soils, of roots from initially sterilized soil, and of soil.

1.1 Import data files

The analysis of bacterial and fungal community compositions in R requires the following input files:
- Experimental design: *Design_file_bacteria.csv* and *Design_file_fungi.csv*
contains the meta information of the sequenced samples. Called *b_DESIGN* and *f_DESIGN* in the R script.
- ASV table: *bacteria_ASV.tab* and *fungi_ASV.tab*
contains the number of ASV sequences per sample. Called *b_COUNT* and *f_COUNT* in the R script.
- Taxonomy: *bacteria_taxa.tab* and *fungi_taxa.tab*
contains the taxonomic information for all ASVs. Called *b_TAX* and *f_TAX* in the R script.

The samples for this publication were sequenced together with samples from other projects in the same sequencing run. On github, the design.xslx file denotes which samples belong to this experiment (https://github.com/PMI-Basel/Stengele_et_al_At_BX-feedbacks/Microbiota_Analysis/1_start). Therefore, we first subset the data sets to only contain the samples from this project, and we also remove the ASVs that were not detected in this project.

The experiment contains six sample groups:
- BX+ soil samples
- BX- soil samples
- Arabidopsis roots grown in native BX+ soil
- Arabidopsis roots grown in native BX- soil
- Arabidopsis roots grown in sterilized BX+ soil
- Arabidopsis roots grown in sterilized BX- soil

These sample groups are represent three different sample types:
- soil samples
- root samples grown in native soils
- root sampels grown in sterilized soils

1.1.1 Bacteria

After import, we filter the *b_TAX* table to remove non-bacterial ASVs, such as some Eukarya sequences, Cyanobacteria or Mitochondria.

The bASVs that were filtered out from the *b_TAX* (non-Bacteria, Cyanobacteria, Mitochondria) were also deleted from the *b_COUNT* file. The rownames (=samples) of the *b_COUNT* file were also ordered in the same way as the samples are ordered in the *b_DESIGN*.

Number of bASV in the *b_COUNT* after taxonomic filtering: **17'129**.

1.1.2 Fungi

The rownames (=samples) of the *f_COUNT* table were ordered in the same way as the samples are ordered in the *f_DESIGN*. Number of fASVs in f_COUNT: **1'802**.

1.2 Colours

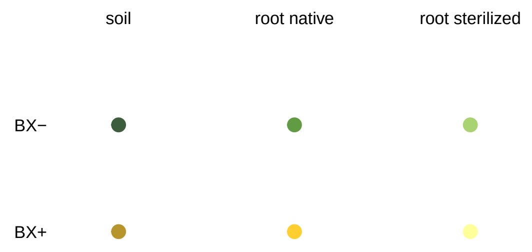

2. Data normalization

2.1. Bacteria

A total of 2’630’507 bacterial sequences were generated, with a median sequence number of 35’109 per sample (21’881 to 53’527).

Figure 1 shows the different sequencing depths across samples, and the black horizontal line shows the lowest read number per sample with 21’881 reads.

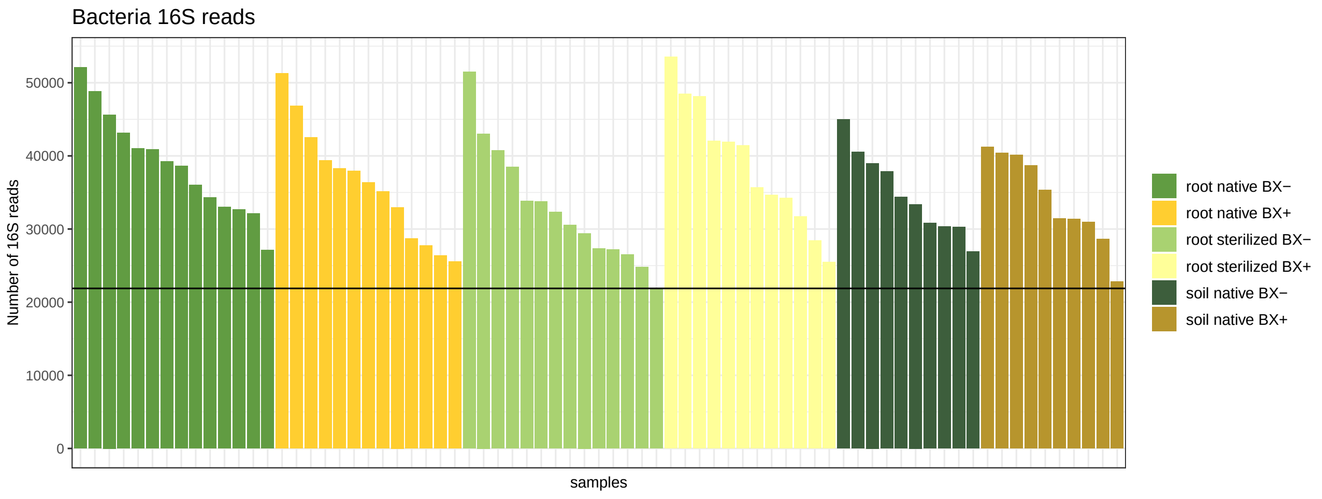

Figure 1: Number of 16S reads per sample

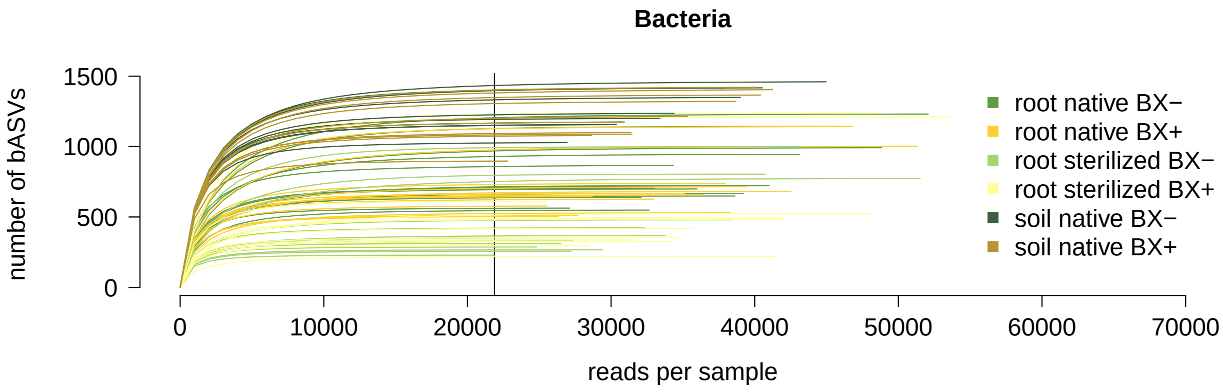

Figure 2: Rarefaction curves for 16S samples

As shown in Figure 2, all samples were sequenced deep enough, as the number of bASVs is no longer increasing with higher number of reads per sample. The black line again depicts the lowest read number per sample.

For sample normalization, we follow the recommendations by Weiss et al., (2015, PeerJ), which states that the data should be rarefied if the number of samples differ between experimental groups. Therefore,we perform a kruskal-wallis test to see if the read numbers are significantly different among groups.

```
##
##  Asymptotic Kruskal-Wallis Test
##
## data:  rowSums(b_COUNT) by
##   b_DESIGN$group (root native BX-, root native BX+, root sterilized BX-, root sterilized BX+, soil nati
## chi-squared = 7.1885, df = 5, p-value = 0.207
```

We do not find significant differences in read numbers among groups. Thus, we do not rarefy our data, but normalize it with total sum scaling (tss) instead.

Table 1 displays the number of 16S samples per group.

Table 1: Number of samples for the bacterial community analysis

	root_native	root_sterilized	soil_native
**BX+**	13	12	10
**BX-**	14	14	10

2.2 Fungi

A total of 207'140 fungal sequences were generated, with a median sequence number of 2'249 per sample (117 to 7'804).

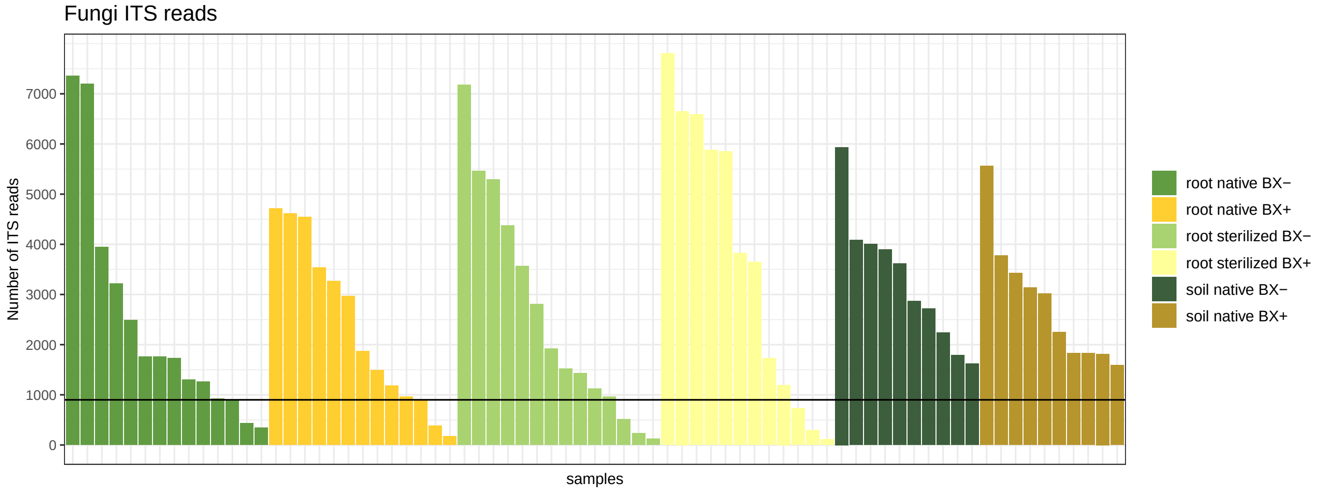

Figure 3: Number of reads per sample.

As shown in Figure 3, some samples have low read numbers below 900 reads per sample.

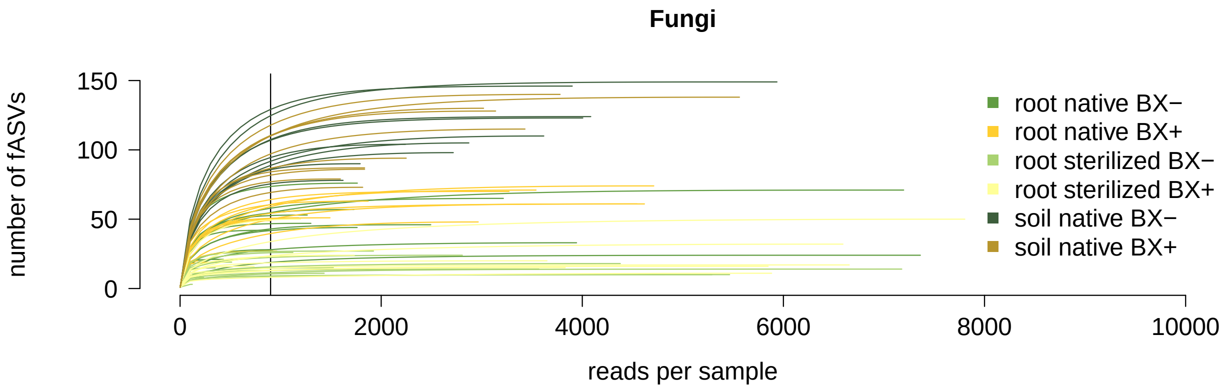

Figure 4: Rarefaction curves for fungal samples

For the fungal sequencing, most samples have an adequate sequencing depth as the number of fASVs no longer increases with higher reads per sample. However, we exclude 11 samples that do not have a minimum sequencing depth of 900 from further analysis. We also remove ASVs that no longer occur in the remaining samples. See Table 2 for the remaining ITS sample numbers per group.

Table 2: Number of samples for the fungal community analysis

	root_native	root_sterilized	soil_native
**BX+**	11	9	10
**BX-**	11	11	10

For the fungal data, we also perform a kruskal-wallis test with the remaining samples to test if read numbers differ between sample groups

```
##
##  Asymptotic Kruskal-Wallis Test
##
## data:  rowSums(f_COUNT_excl) by
##   f_DESIGN_excl$group (root native BX-, root native BX+, root sterilized BX-, root sterilized BX+, soil
## chi-squared = 5.8986, df = 5, p-value = 0.3162
```

We also do not find significant differences in read numbers among groups for the fungal samples. Thus, we do not rarefy our data, but normalize it with total sum scaling (tss).

3. Microbiota Analysis Results

3.1 Alpha Diversity

We examined alpha (= within groups) diversity patterns using the indices of Chao's estimate of richness, Shannon diversity and Pielou's evenness using the 'estimate_richness' function of the r-package 'phyloseq'. We were interested in alpha diversity patterns between sample types (roots and soil), and if BX-conditioning of the soil would affect microbial diversity both in soil and on Arabidopsis roots.

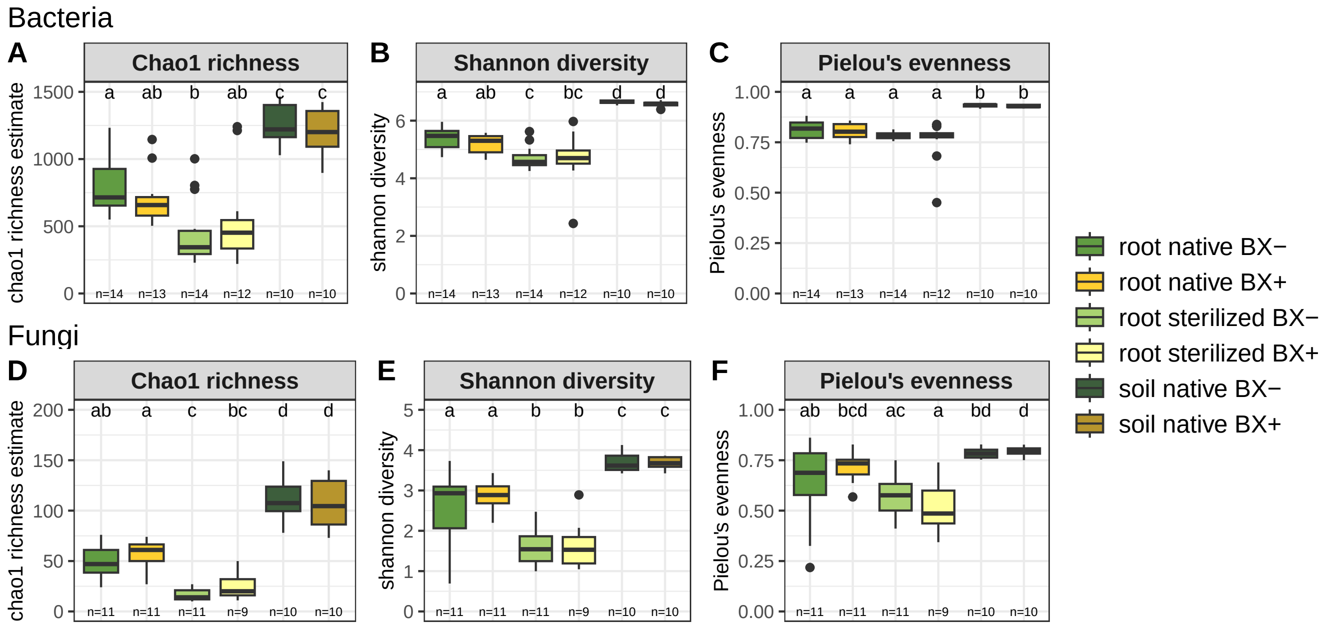

Figure 5: Alpha diversity

Alpha diversity for bacterial and fungal communities is lowest in roots grown in sterilized soils, followed by roots from native soils, and highest in soils for all diversity metrics. There is no difference between the conditioning for any of the sample types. The letters signify statistical differences evaluated by an analysis of variance (ANOVA) followed by the post-hoc Tukey HSD test.

3.2 Beta diversity

We examined the data for beta diversity patterns (= between groups), with a special interest in the effect of soil conditioning.

3.2.1 PERMANOVA

We utilized Permutational Multivariate Analysis of Variance (PERMANOVA) to partition and quantify the effects of the experimental factors. Our experimental factors include *treatment* (native vs. sterilized), *compartment* (soil vs. root) and *conditioning* (BX+ vs BX-). First, we analysed the whole data set and tested for the effects of the *compartment* and the *conditioning* by running the model ~ *compartment * conditioning.* For this we utilized the function 'adonis' of the r-package 'vegan' with a Bray Curtis distance matrix as input and running 99'999 permutations.

Table 3: Bacteria: PERMANOVA all samples

	Df	SumOfSqs	R^2	F	Pr(>F)
compartment	1	5.627	0.234	21.772	0.000
conditioning	1	0.363	0.015	1.406	0.154
compartment:conditioning	1	0.243	0.010	0.939	0.432
Residual	69	17.834	0.741	NA	NA
Total	72	24.067	1.000	NA	NA

We find that the compartment explains 23.4% of the variance in bacterial community composition (P = 1e-05), while the conditioning has no significant effect when all sample groups are analysed together (R^2 = 0.015, P = 0.154). There is also no significant interaction between compartment and conditioning (R^2 = 0.01, P = 0.432; Table 3).

Table 4: Fungi: PERMANOVA all samples

	Df	SumOfSqs	R^2	F	Pr(>F)
compartment	1	4.377	0.177	12.986	0.000
conditioning	1	0.472	0.019	1.400	0.104
compartment:conditioning	1	0.263	0.011	0.782	0.751
Residual	58	19.548	0.793	NA	NA
Total	61	24.660	1.000	NA	NA

Similarly, the compartment explains 17.7% of the variance in fungal community composition (P = 1e-05), while the conditioning has no significant effect (R^2 = 0.019, P = 0.104). For the fungal community composition, the interaction between compartment and conditioning does also not explain a significant portion of the variance (R^2 = 0.011, P = 0.751; Table 4).

Next, we also wanted to partition the effect of the *treatment* (= grown in native or sterilized soil) on microbial communities on roots, in combination with the *conditioning* variable. For this, we used a subset of the data only containing samples from the root compartment, and used the model ~ *treatment * conditioning.*

Table 5: Bacteria: PERMANOVA root samples

	Df	SumOfSqs	R^2	F	Pr(>F)
treatment	1	5.191	0.313	23.930	0.000
conditioning	1	0.371	0.022	1.709	0.074
treatment:conditioning	1	0.386	0.023	1.782	0.065
Residual	49	10.628	0.641	NA	NA
Total	52	16.576	1.000	NA	NA

We find that the treatment explains 31.3% of the variance in community composition (P = 1e-05). The size of this effect is thus comparable to the size of the compartment effect, i.e. the overall difference between root and soil samples, when all sample groups were included in the analysis (see Table 3). Thus, bacterial communities of roots grown in native soil are markedly different from bacterial communities of roots grown in sterilized soil. The conditioning has no significant effect ($R^2 = 0.022$, $P = 0.074$), and there is also no significant interaction between treatment and conditioning ($R^2 = 0.023$, $P = 0.065$; Table 5).

Table 6: Fungi: PERMANOVA root samples

	Df	SumOfSqs	R^2	F	Pr(>F)
treatment	1	1.946	0.118	5.506	0.000
conditioning	1	0.539	0.033	1.524	0.049
treatment:conditioning	1	0.635	0.038	1.798	0.016
Residual	38	13.430	0.811	NA	NA
Total	41	16.551	1.000	NA	NA

The fungal communities between roots grown in native versus sterilized soils also differ with treatment explaining 12 % of the variance (P = 1e-05). However, the treatment effect on bacterial communities was still larger. The conditioning for all root samples has a small but significant effect on the fungal communities, explaining 3.3% of the variance ($P = 0.049$), while the interaction between treatment and conditioning can explain 3.8% of the variance ($P = 0.016$).

3.2.2 PCoA - unconstrained ordination

To visualize the diversity of the different sample groups, we plot all samples together in a Principal Coordinate Analysis (PCoA) plot based on Bray Curtis distances.
We also report these graphs in the main part of the manuscript in Figure 3.

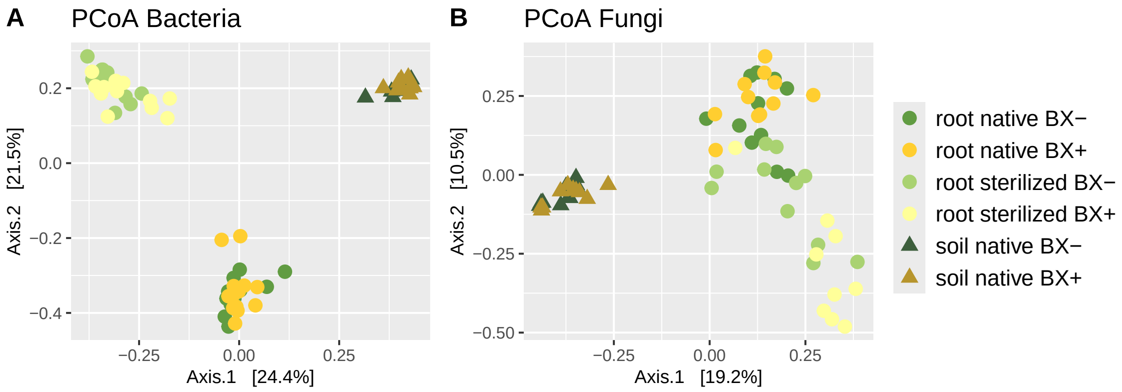

Figure 6: Beta diversity - PCoA - all groups

As already evidenced with the PERMANOVA test, the three sample groups are clearly separated for bacterial communities. For the fungal communities, the effect of the *treatment* was less strong, as fungal communities from roots grown in native soil and from roots grown in sterilized soil are less separated than the bacterial communities.

3.2.3 CAP - constrained ordination

We also used constrained ordination (partial canonical analysis of principal coordinates; CAP) to evaluate how the variation in the dataset can be partitioned to the *conditioning* variable. We analysed this for each sample type separately and validated the significance of the model with ANOVA using 99'999 permutations.

Figure 7 reports the CAPs based on Bray Curtis distances with the title stating the amount and the statistical significance of the explained variation by the *conditioning* variable.

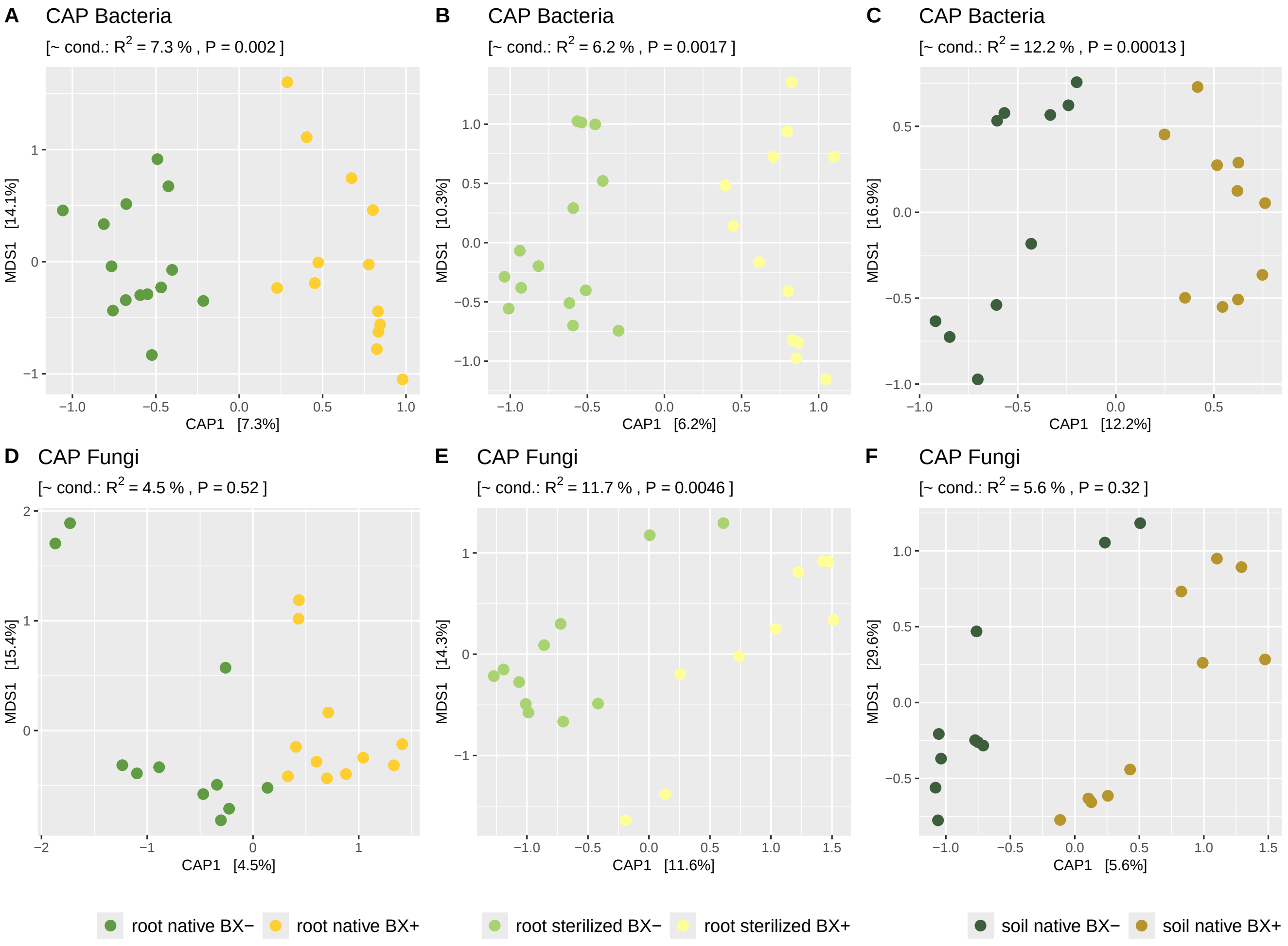

Figure 7: Beta diversity - CAP - individual sample groups

For the bacterial communities, the conditioning significantly explains 7% of the variance on roots from native soil ($P = 0.002$; Table 7). On roots from sterilized soil, conditioning explains 6% of the variance ($P = 0.00173$; Table 8), and in soil 12% of the variance is explained by conditioning ($P = 0.00013$; Table 9).

Table 7: Bacteria: PERMANOVA root samples from native soil

	Df	SumOfSqs	R^2	F	Pr(>F)
conditioning	1	0.311	0.073	1.975	0.002
Residual	25	3.936	0.927	NA	NA
Total	26	4.247	1.000	NA	NA

Table 8: Bacteria: PERMANOVA root samples from sterilized soil

	Df	SumOfSqs	R^2	F	Pr(>F)
conditioning	1	0.446	0.062	1.6	0.002
Residual	24	6.692	0.938	NA	NA
Total	25	7.138	1.000	NA	NA

Table 9: Bacteria: PERMANOVA soil samples

	Df	SumOfSqs	R^2	F	Pr(>F)
conditioning	1	0.227	0.122	2.495	0
Residual	18	1.637	0.878	NA	NA
Total	19	1.864	1.000	NA	NA

For the fungi, the conditioning does not lead to significant differences in community composition on roots from native soil ($R^2 = 0.0452$, $P = 0.524$; Table 10) and in soil ($R^2 = 0.0561$, $P = 0.319$; Table 12), while it explains 12 % of the variance on roots grown in sterilized soils ($P = 0.0046$; Table 11).

Table 10: Fungi: PERMANOVA root samples from native soil

	Df	SumOfSqs	R^2	F	Pr(>F)
Model	1	0.334	0.045	0.947	0.525
Residual	20	7.063	0.955	NA	NA
Total	21	7.397	1.000	NA	NA

Table 11: Fungi: PERMANOVA root samples from sterilized soil

	Df	SumOfSqs	R^2	F	Pr(>F)
Model	1	0.840	0.117	2.374	0.005
Residual	18	6.368	0.883	NA	NA
Total	19	7.207	1.000	NA	NA

Table 12: Fungi: PERMANOVA soil samples

	Df	SumOfSqs	R^2	F	Pr(>F)
Model	1	0.209	0.056	1.069	0.32
Residual	18	3.523	0.944	NA	NA
Total	19	3.732	1.000	NA	NA

3.3 Phyla distribution

To get a better understanding for the community compositions, we visualize the relative abundances of the main bacterial and fungal taxa for each sample. For the bacterial phylum “Proteobacteria”, we show the abundance of the different classes instead.

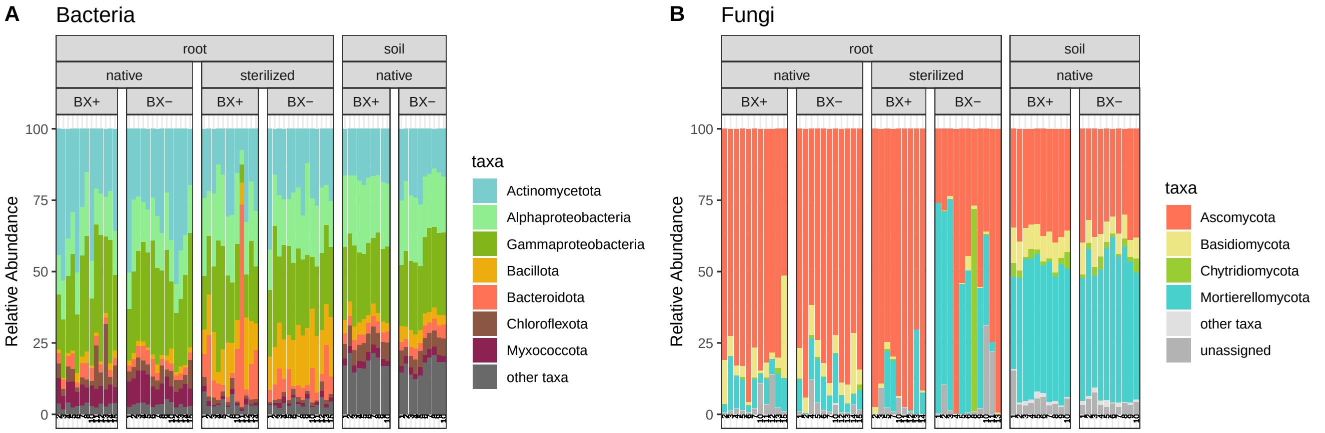

Figure 8: Relative abundance of main bacterial and fungal taxa per sample

Compared to soil, bacterial communities on roots have more Actinobacteria and more Deltaproteobacteria. Bacterial communities on roots grown in native soil have more Deltaproteobacteria and more Chloroflexi compared to communities of roots grown in sterilized soil, where instead the Firmicutes are more abundant.

Fungal communities on roots from native soil are clearly dominated by the Ascomycota, while communities in soil are composed of ca. 50% of Mortierellomycota. The pattern on roots from sterilized roots is more diverse, with certain root samples from sterilized BX- soil having more Mortierellomycota and other samples are more dominated by Ascomycota.

3.4 Determination of differently abundant ASVs

In a next step, we identified ASVs that differ in their relative abundance between BX+ and BX- conditioning in each sample type. Bacterial ASVs are refered to as bASVs and fungal ASVS we refer to as fASVs. For this analysis, we used four different tools to measure differential abundance, namely aldex2, acombc, maaslin2 and metagenomeSeq. We define an ASV to be differently abundant if it is detected by three or more tools.

3.4.1 bASVs on roots grown in native soil

Table 13: BX-conditioning effect

lower in BX+	unchanged	higher in BX+
8	1394	3

11 bASVs are differently abundant, which corresponds to 2.44% of the bacterial community on roots grown in native soil that was changed due to BX-conditioning (= total abundance of all differently abundant bASVs on roots from BX+ soil). The taxonomies of these differently abundant bASVs is shown in Table 20 (at the end of this document), and are also reported in Supplementary Table S4.

3.4.1.1 Rank-abundance plot

To investigate the abundance pattern of the most common bASVs, we plot the 50 top abundant bASVs (in BX+) and label the BX-dependent bASVs with an asterix. Of the 11 differently abundant bASVs, 1 is ranked within the 50 most abundant bASVs on roots grown in BX+ soil.

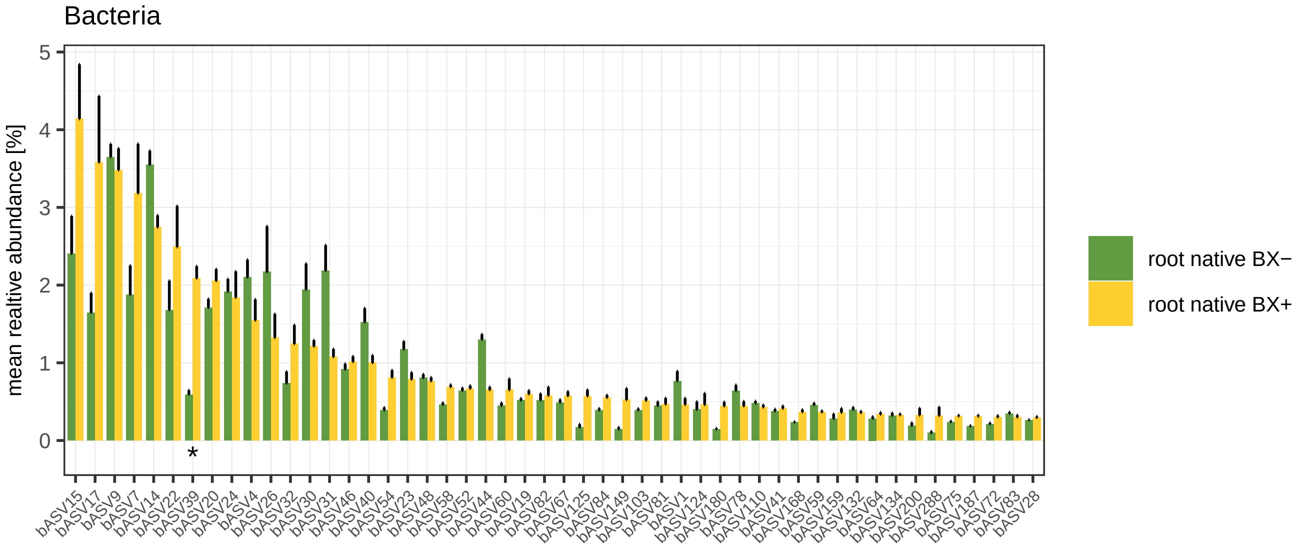

Figure 9: Rank abundance plot of bASVs from roots grown in native soil

3.4.1.2 bASVs and taxonomy

We also explore the taxonomic distribution of bASVs affected by the conditioning on roots of Arabidopsis.

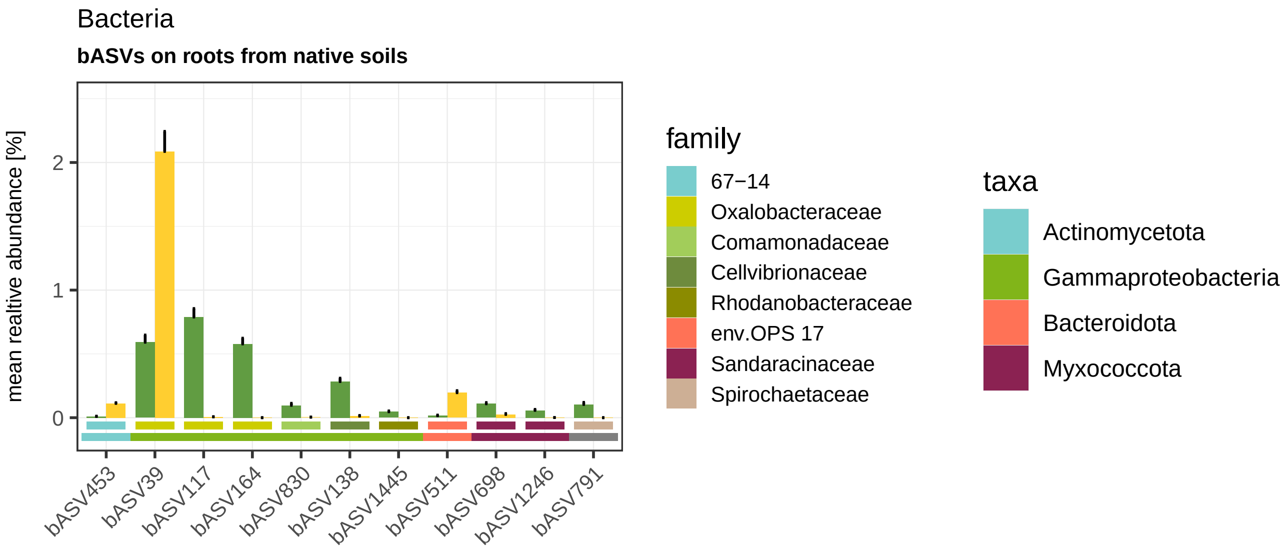

Figure 10: Abundance plot of differential bASVs from roots grown in native soil

3.4.2 bASVs on roots grown in sterilized soil

Table 14: BX-conditioning effect

lower in BX+	unchanged	higher in BXp
0	915	0

No bASVs on roots grown in sterilized soil are differently abundant between BX+ and BX- conditioning.

3.4.2.1 Rank-abundance plot

We plot the 50 top abundant ASVs (in BX+).

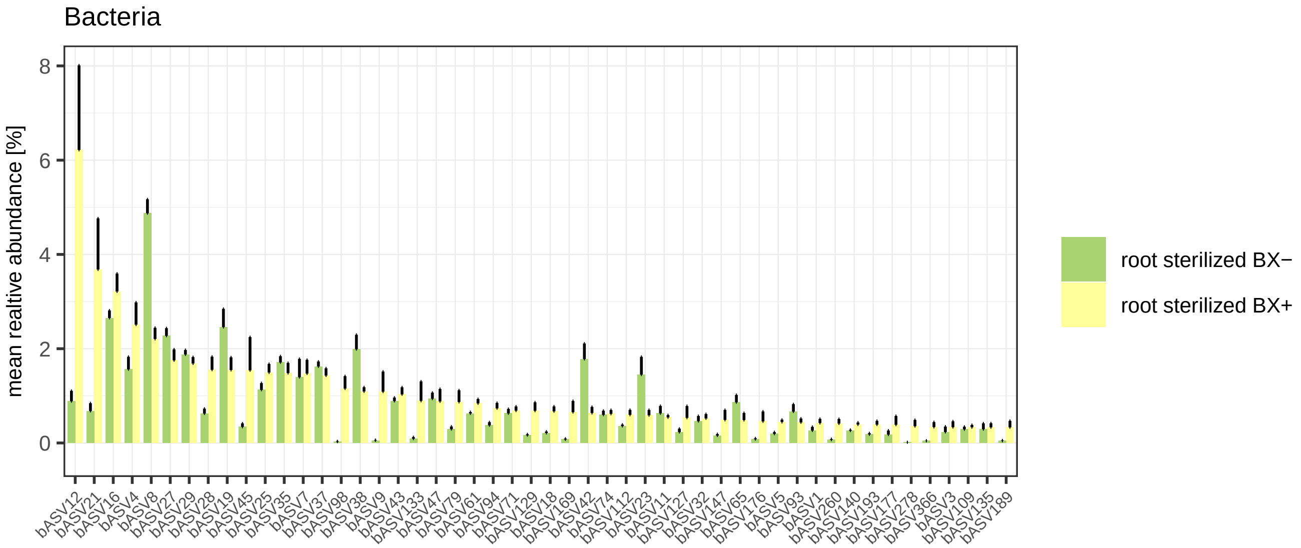

Figure 11: Rank abundance plot of bASVs from roots grown in sterilized soil

3.4.3 bASVs in soil

Table 15: BX-conditioning effect

lower in BX+	unchanged	higher in BX+
8	2078	16

24 bASVs are differently abundant in soil, which corresponds to 3.6% of the bacterial community in native BX+ soil. The taxonomies of these differentially abundant bASVs are shown in Table 21 (at the end of this document) and they are also reported in Supplementary Table S3. We plot the 50 top abundant ASVs (in BX+) and label the BX-dependent bASVs with an asterix. Of the 24 differently abundant bASVs, 4 are ranked within the 50 most abundant bASVs in BX+ soil.

3.4.3.1 Rank-abundance plot

We plot the 50 top abundant ASVs (in BX+).

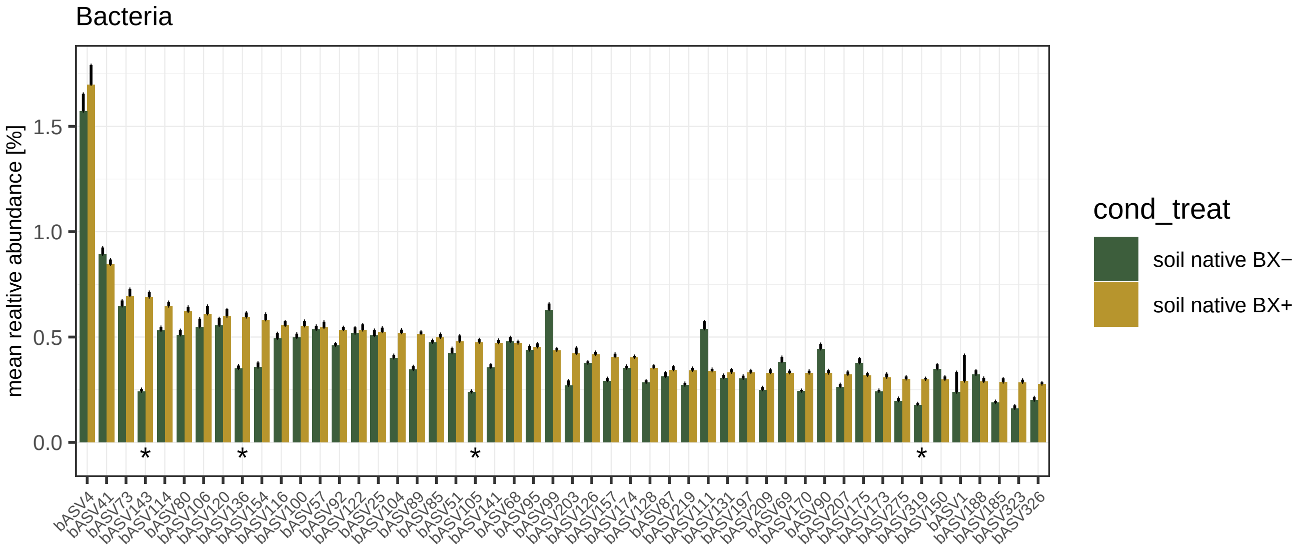

Figure 12: Rank abundance plot of bASVs from soil

3.4.3.2 bASVs and taxomony

We also explore the taxonomic distribution of bASVs affected by the conditioning in soils.

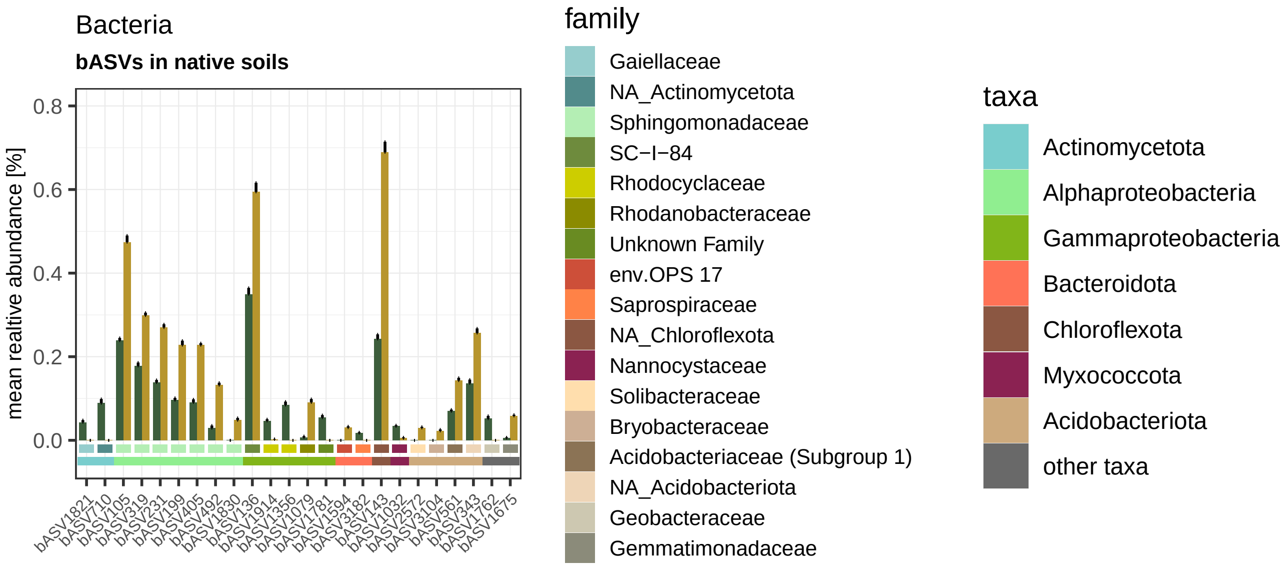

Figure 13: Abundance plot of differential bASVs from native soil

3.4.4 bASVs overlap

We also investigated if the same bASVs would be BX-sensitive in soil and on roots, i.e. if there is any overlap between the bASVs differentially abundant between BXplus and BXminus soil and the differentially abundant bASVs between roots from native BXplus and roots from native BXminus soil.

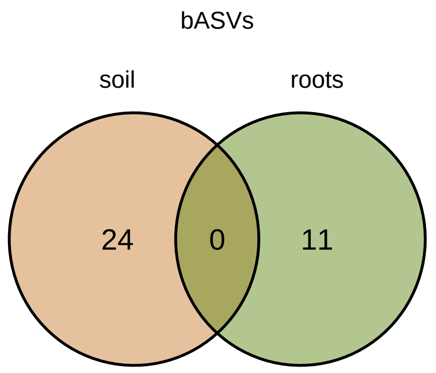

Figure 14: Overlap of differential bASVs from native soil and roots from native soil

There is no overlap between differentially abundant bASVs in soil and bASVs on roots from native soils. Therefore, BX-conditioning affects both bASVs in soil and bASVs on roots, but these are two seperate sets of bASVs.

3.4.5 fASVs on roots grown in native soil

Table 16: BX-conditioning effect

lower in BX+	unchanged	higher in BX+
0	97	0

No fASVs are differently abundant on roots grown in native soils between BX+ and BX-.

3.4.5.1 Rank-abundance plot

We plot the 50 top abundant ASVs (in BX+).

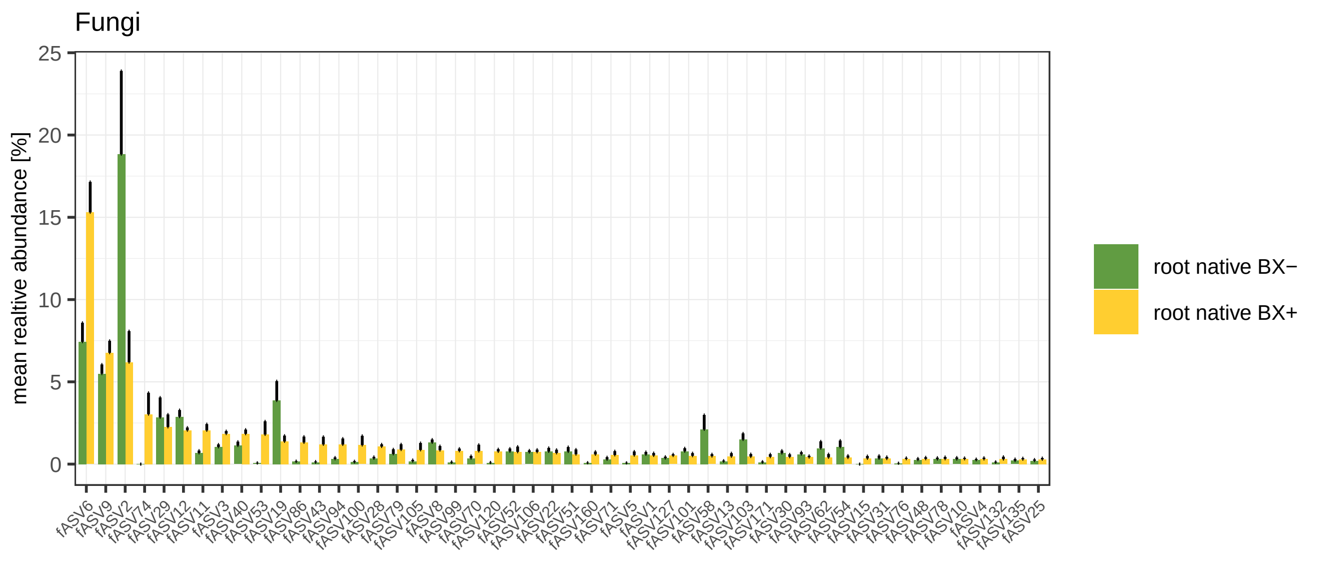

Figure 15: Rank abundance plot of fASVs from roots grown in native soil

3.4.6 fASVs on roots grown in sterilized soil

Table 17: BX-conditioning effect

lower in BX+	unchanged	higher in BX+
0	33	0

No fASVs on roots grown in sterilized soil had a significantly different abundance between BX+ and BX- conditioning.

3.4.6.1 Rank-abundance plot

We plot the 30 top abundant fASVs (in BX+).

3.4.7 fASVs in soil

Table 18: BX-conditioning effect

lower in BX+	unchanged	higher in BX+
2	194	0

2 fASVs are differentially abundant, which corresponds to 1.46% of the fungal community in BX+ soil.

The taxonmies of the differently abundant fASVs are shown in Table 19.

3.4.7.1 Rank-abundance plot

We plot the 50 top abundant fASVs (in BX+) and label the BX-dependent fASVs with an asterix. Both differently abundant fASVs rank among the 50 most abundant fASVs in BX+ soil.

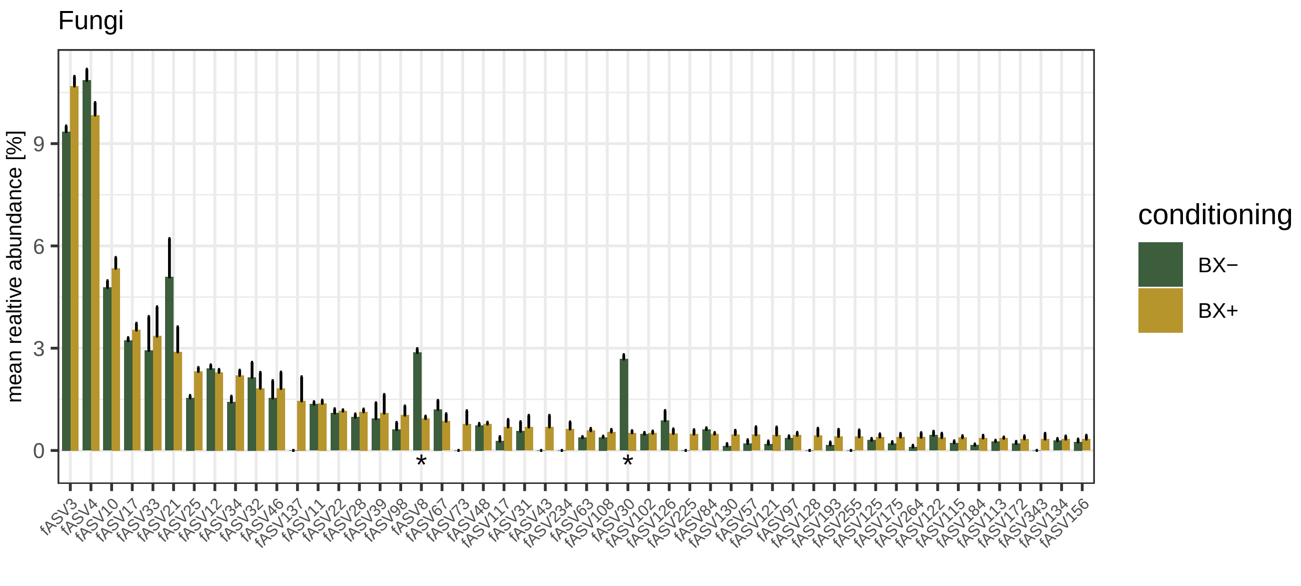

Figure 16: Rank abundance plot of fASVs from soil

Table 19: Top fASVs from soil that are differently abundant between the conditionings

ASV	order	family	genus	abund. BX+	abund. BX-
fASV8	Mortierellales	Mortierellaceae	Mortierella	0.94%	2.88%
fASV30	Mortierellales	Mortierellaceae	Mortierella	0.52%	2.68%

Table 20: Top bASVs from roots grown in native soil that are differently abundant between the conditionings

ASV	phylum/class	family	genus	ab. BX+	ab. BX-
bASV39	Gammaproteobacteria	Oxalobacteraceae	Massilia	2.09%	0.59%
bASV511	Bacteroidota	env.OPS 17	unassigned	0.2%	0.02%
bASV453	Actinomycetota	67-14	unassigned	0.11%	0.01%
bASV698	Myxococcota	Sandaracinaceae	unassigned	0.03%	0.11%
bASV138	Gammaproteobacteria	Cellvibrionaceae	Cellvibrio	0.01%	0.28%
bASV117	Gammaproteobacteria	Oxalobacteraceae	Massilia	0.01%	0.79%
bASV830	Gammaproteobacteria	Comamonadaceae	unassigned	0%	0.1%
bASV1246	Myxococcota	Sandaracinaceae	Sandaracinus	0%	0.06%
bASV164	Gammaproteobacteria	Oxalobacteraceae	Massilia	0%	0.58%
bASV791	Spirochaetota	Spirochaetaceae	Spirochaeta 2	0%	0.1%
bASV1445	Gammaproteobacteria	Rhodanobacteraceae	Ahniella	0%	0.05%

Table 21: Top bASVs from soil that are differently abundant between the conditionings

ASV	phylum/class	family	genus	ab. BX+	ab. BX-
bASV143	Chloroflexota	unassigned	unassigned	0.69%	0.24%
bASV136	Gammaproteobacteria	SC-I-84	unassigned	0.59%	0.35%
bASV105	Alphaproteobacteria	Sphingomonadaceae	Sphingomonas	0.47%	0.24%
bASV319	Alphaproteobacteria	Sphingomonadaceae	Sphingomonas	0.3%	0.18%
bASV231	Alphaproteobacteria	Sphingomonadaceae	Sphingomonas	0.27%	0.14%
bASV343	Acidobacteriota	unassigned	unassigned	0.26%	0.14%
bASV199	Alphaproteobacteria	Sphingomonadaceae	Sphingomonas	0.23%	0.1%
bASV405	Alphaproteobacteria	Sphingomonadaceae	Sphingomonas	0.23%	0.09%
bASV561	Acidobacteriota	Acidobacteriaceae (sg1)	Occallatibacter	0.14%	0.07%
bASV492	Alphaproteobacteria	Sphingomonadaceae	Sphingomonas	0.13%	0.03%
bASV1079	Gammaproteobacteria	Rhodanobacteraceae	Rhodanobacter	0.09%	0.01%
bASV1675	Gemmatimonadota	Gemmatimonadaceae	unassigned	0.06%	0.01%
bASV1830	Alphaproteobacteria	Sphingomonadaceae	Croceibacterium	0.05%	0%
bASV1594	Bacteroidota	env.OPS 17	unassigned	0.03%	0%
bASV2572	Acidobacteriota	Solibacteraceae	Candidatus Solibacter	0.03%	0%
bASV3104	Acidobacteriota	Bryobacteraceae	Bryobacter	0.02%	0%
bASV1032	Myxococcota	Nannocystaceae	Nannocystis	0%	0.03%
bASV1914	Gammaproteobacteria	Rhodocyclaceae	Candidatus Accumulibacter	0%	0.05%
bASV710	Actinomycetota	unassigned	unassigned	0%	0.09%
bASV1356	Gammaproteobacteria	Rhodocyclaceae	Azovibrio	0%	0.08%
bASV1762	Thermodesulfobacteriota	Geobacteraceae	Geomonas	0%	0.05%
bASV1781	Gammaproteobacteria	Unknown Family	Acidibacter	0%	0.05%
bASV1821	Actinomycetota	Gaiellaceae	Gaiella	0%	0.04%
bASV3182	Bacteroidota	Saprospiraceae	unassigned	0%	0.02%
