# Supplementary material for: Benzoxazinoid‐mediated microbiome feedbacks enhance Arabidopsis growth and defence
Source: New Phytol. 2026 Mar 17;250(5):3334–48. doi: 10.1111/nph.71098 (PMC13150306; doi:10.1111/nph.71098)
Supplement: Supplementary file 2 — Dataset S2 Transcriptome analysis. [file NPH-250-3334-s004.pdf]

Stengele et al Dataset S2: Transcriptome analysis

Lea Stauber, Katja Stengele

2025-12-22

Contents

Experimental background **1**

Results **1**

DESeq2 1

PCA plots 2

DESeq2 table 3

MA plot 4

Overlap Roots and Shoots 5

Coseq Analysis 5

Determine number of clusters 5

Shoots 6

Roots 7

GO term analysis 7

Shoots 8

Roots 9

Dotplots of GO terms with high gene ratio 10

Experimental background

Arabidopsis thaliana Col-0 plants were grown on BX+ and BX- soil for seven weeks on soil batch 2 (in Experiment V.II). Then, roots were harvested from soil, the soil clumps on the roots were removed, and both roots and shoots were snap frozen in liquid nitrogen. Next, RNA was extracted with the NucleoSpin RNA Kit (Macherey-Nagel), libraries were prepared and sequenced at paired-end 51 bp read length on the Illumina NovaSeq6000 platform (Illumina, San Diego, USA) at the Genomics Facility Basel (https://bsse.ethz.ch/genomicsbasel). After quality control, reads were aligned to the Arabidopsis thaliana TAIR10 reference genome and read count was performed using STAR 2.7.9a (Dobin et al., 2013).

Results

DESeq2

We run the DESeq2 analysis for root and shoot samples individually to determine differentially expressed genes between the conditioning. To run the DESeq2 analysis, we use the custom function *DESeq2_analysis_conditioning()*, which uses the *DESeqDataSetFromMatrix()* function to merge the count data with the coldata, sets the "BX-" condition as a reference, and uses the *DESeq()* function to compute differential expression. To reduce computing time, the DESeq2 analysis file is computed once, saved as an .RDS file and loaded from the .RDS file into R for further analyses.

PCA plots

We display the samples in a PCA plot to visualize the sample clustering. For this, we use a variance transformation based on the *vst()* function from the DESeq2 package and display the data in a PCA plot.

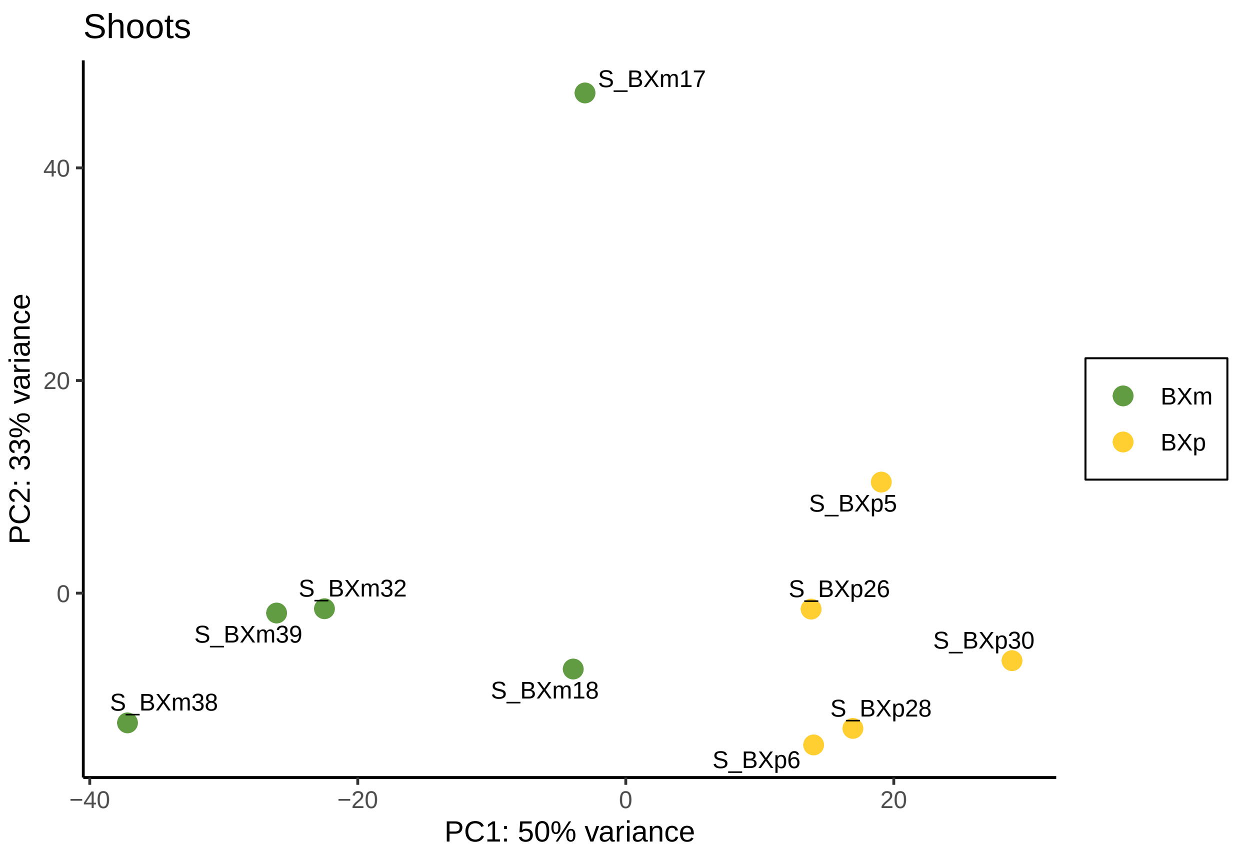

There is a clear within-group clustering of the BX+ shoot samples. The BX- shoot samples do not cluster as closely within the group, but the PC1 axis still separates the BX+ from the BX- samples. The S_BXm17 sample is clustering furthest apart. However, we prefer to keep all samples in the analysis if possible to retain the biological information in the data set.

To get a better overview of the data structure, we check how many outlier genes were identified. Outlier genes are identified by *DESeq2()* using the Cooks distance, where a large distance indicates that one sample strongly influences the mean of the gene. If thousands of genes are identified as outliers, this might suggest that one biological replicate behaves differently and should be removed from the analysis.

```
## [1] "Summary of DESeq2 analysis for shoot samples"
```

```
##
## out of 22863 with nonzero total read count
## adjusted p-value < 0.1
## LFC > 0 (up)       : 3282, 14%
## LFC < 0 (down)     : 4265, 19%
## outliers [1]       : 169, 0.74%
## low counts [2]     : 887, 3.9%
## (mean count < 2)
## [1] see 'cooksCutoff' argument of ?results
## [2] see 'independentFiltering' argument of ?results
```

From 22'863 analysed genes, there are 169 genes defined as outlier genes. As this number is below the suggested threshold where a specific sample should be removed, we will keep S_BXm17 in the analysis.

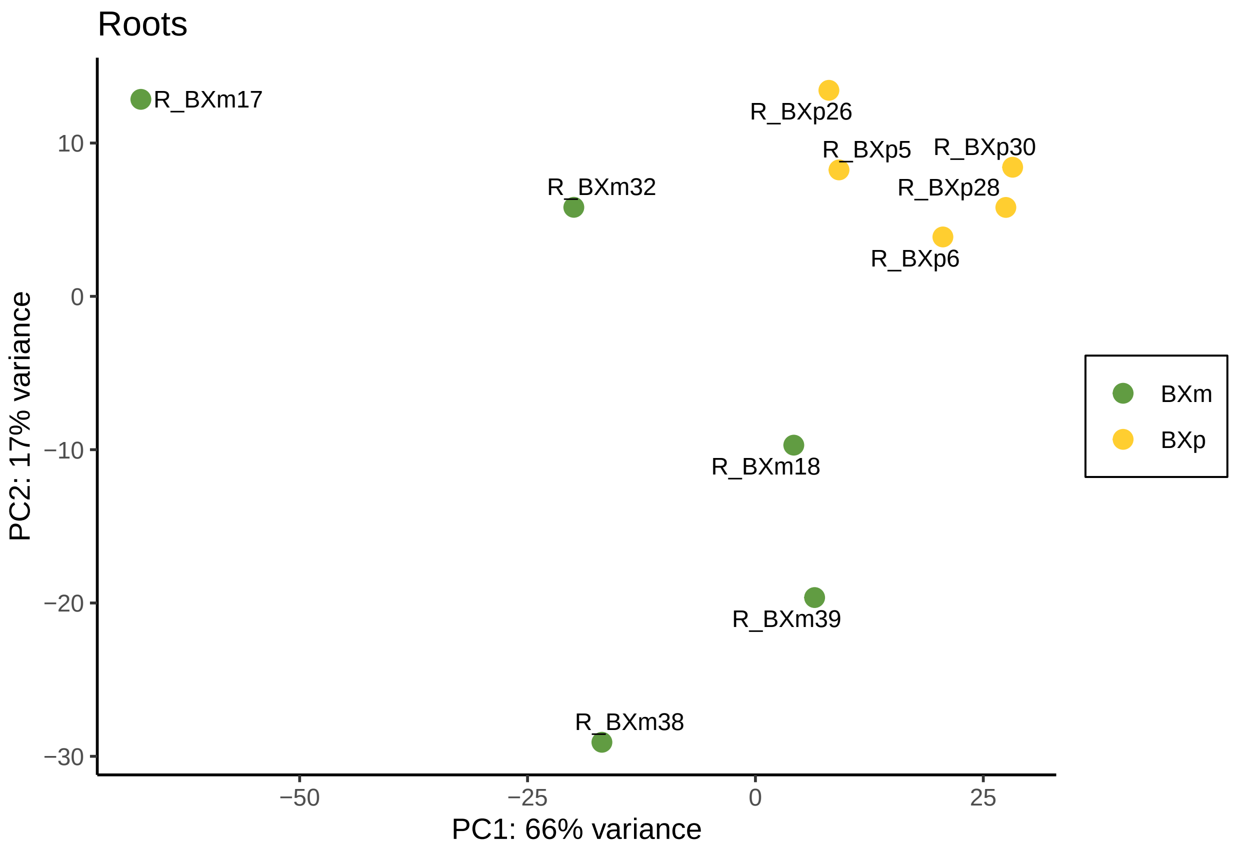

Similarly to the shoot samples, the BX+ root samples also cluster closer together, but the PC1 axis still mainly separates the BX+ from the BX- samples. The R_BXm17 sample is also clustering further apart. Thus, we also check the number of outlier genes in the root data set.

```
## [1] "Summary of DESeq2 analysis for root samples"

##
## out of 24385 with nonzero total read count
## adjusted p-value < 0.1
## LFC > 0 (up)       : 2388, 9.8%
## LFC < 0 (down)     : 2974, 12%
## outliers [1]       : 213, 0.87%
## low counts [2]     : 945, 3.9%
## (mean count < 2)
## [1] see 'cooksCutoff' argument of ?results
## [2] see 'independentFiltering' argument of ?results
```

From 24'385 genes, there are 213 genes defined as outlier genes. Thus, we will also keep R_BXm17 in the analysis.

DESeq2 table

For further analyses, we will only consider genes as differentially expressed if their log2FoldChange (log2FC) is > 1 or < -1, and their adjusted P value (P *adj*) is < 0.05. Furthermore, we remove genes where P *adj* was set to *NA*. We therefore filter the DESeq2 shoot and root data sets accordingly and safe them for the Supplementary tables 5 and 6.

In the shoots, there are 5694 genes differentially expressed (log2FC <-1 OR > 1, P *adj* < 0.05).

In the roots, there are 1839 genes differentially expressed (log2FC <-1 OR > 1, P *adj* < 0.05).

MA plot

We visualize the DESeq2 data with an MA plot, which plots the log2FoldChange (M) data on the y axis and the mean average (A) expressed as the log10(baseMean) on the x axis. Genes that have a log2FoldChange >1 or <-1 are shown in color. These graphs are reported in the main part of the manuscript in Figure 4ac.

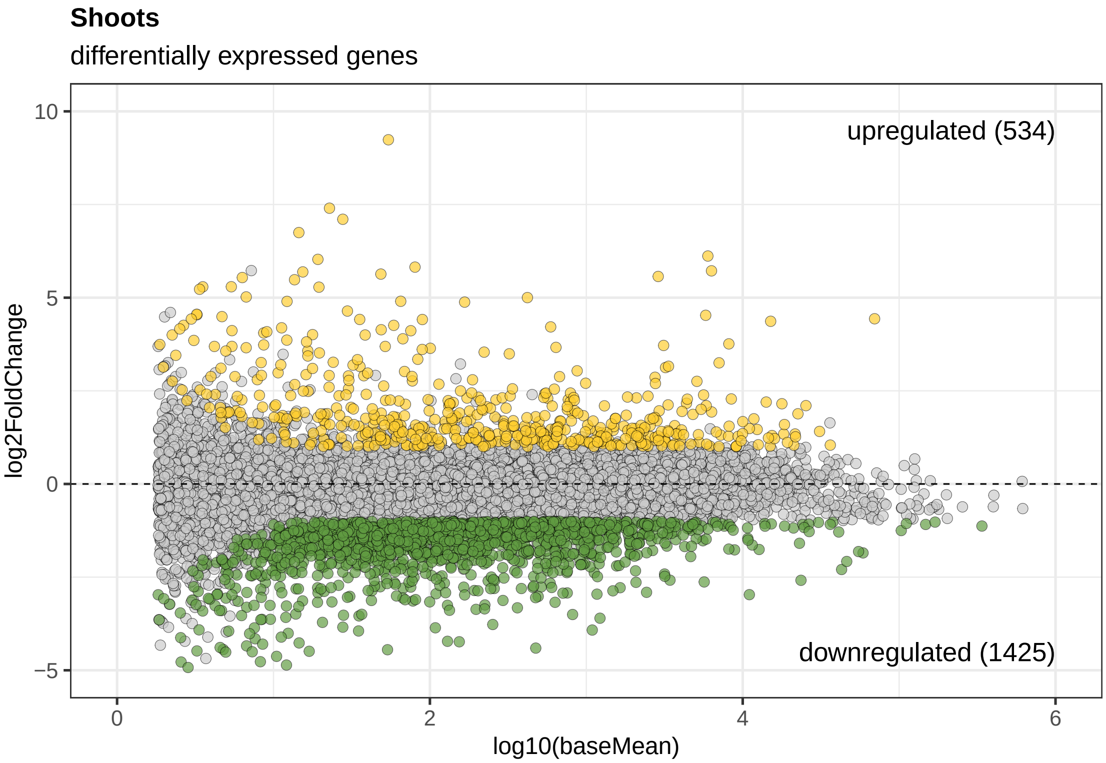

There are more genes downregulated in shoots grown on BXplus soil (1425), than upregulated (534).

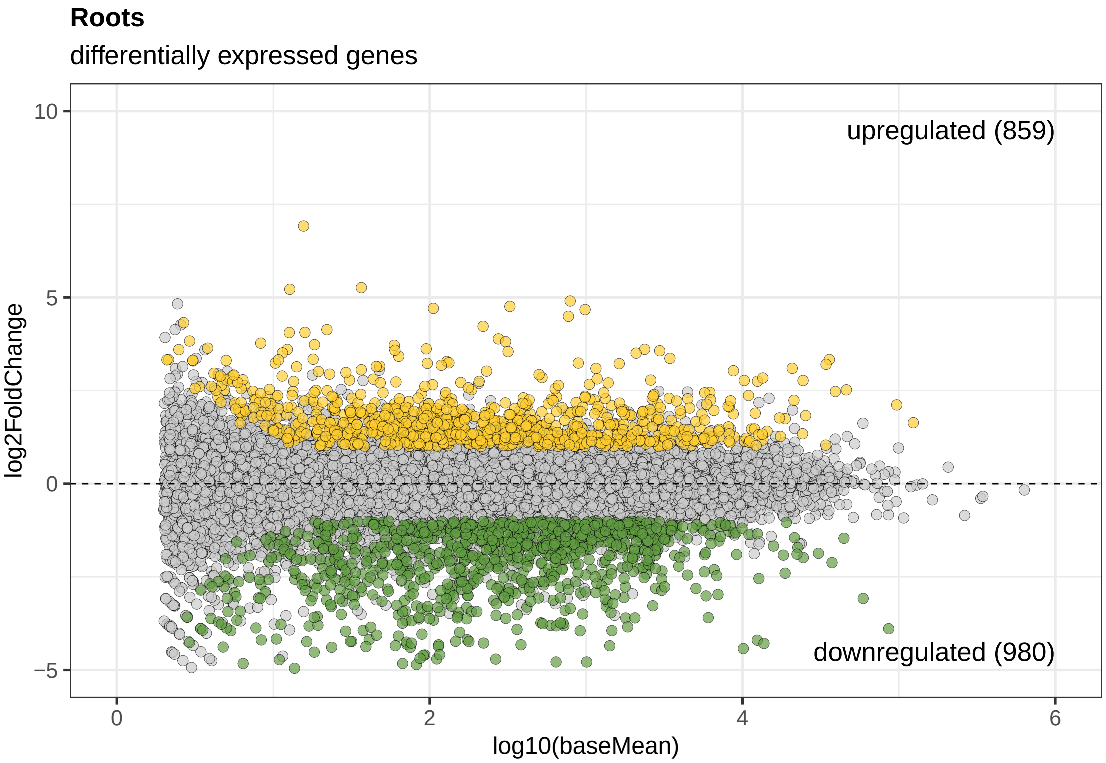

In roots, around the same number of genes are downregulated on BXplus (980) as are upregulated (859).

Overlap Roots and Shoots

We also analyse the overlap of differentially expressed genes between roots and shoots. We consider genes to overlap if they are differentially expressed in both roots and shoots, but the direction (upregulated, downregulated) can also be opposite.

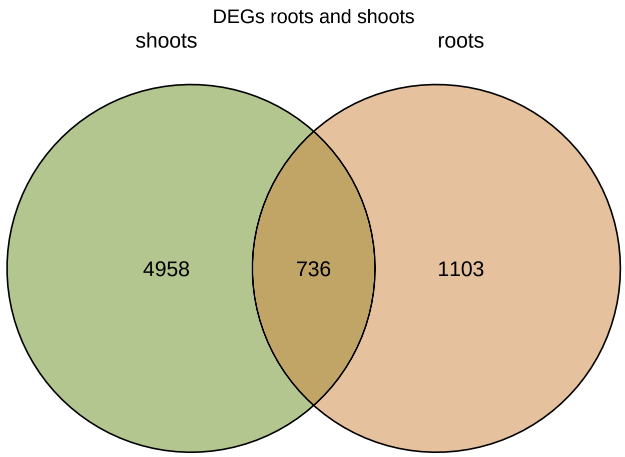

Of the 5694 genes in shoots and 1839 genes in roots that are differentially expressed, 736 genes are differentially expressed in both shoots and roots.

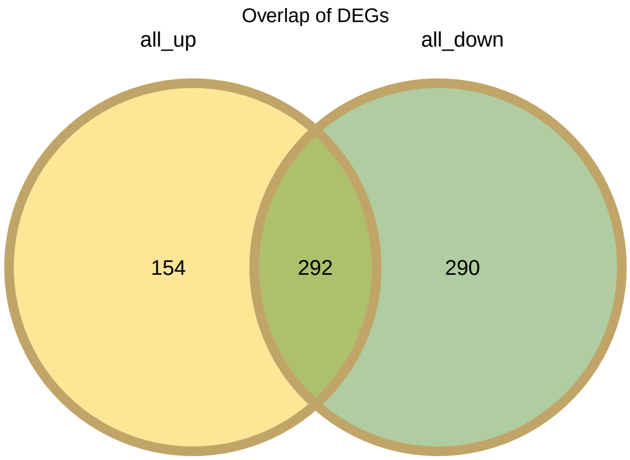

Of this overlap (736 genes), 154 genes are upregulated in both roots and shoots, 290 genes are downregulated in both roots and shoots, and the remaining 292 genes are regulated in opposite directions in the two tissues.

Coseq Analysis

We determine which genes follow the same expression pattern, i.e. are co-expressed, by using the coseq R package.

Determine number of clusters

First, we determine the optimal number of clusters (each containing a set of co-expressed genes), by running the *coseq()* function twenty times. We then select the number of clusters that had been determined optimal by the majority of the twenty coseq runs.

Important: don't rerun this code unless clusters need to be redefined. This analysis takes a lot of resources and time. Also, rerunning the analysis might produce slightly different results, as clusters are re-defined, even if the same seed is used.

Shoots

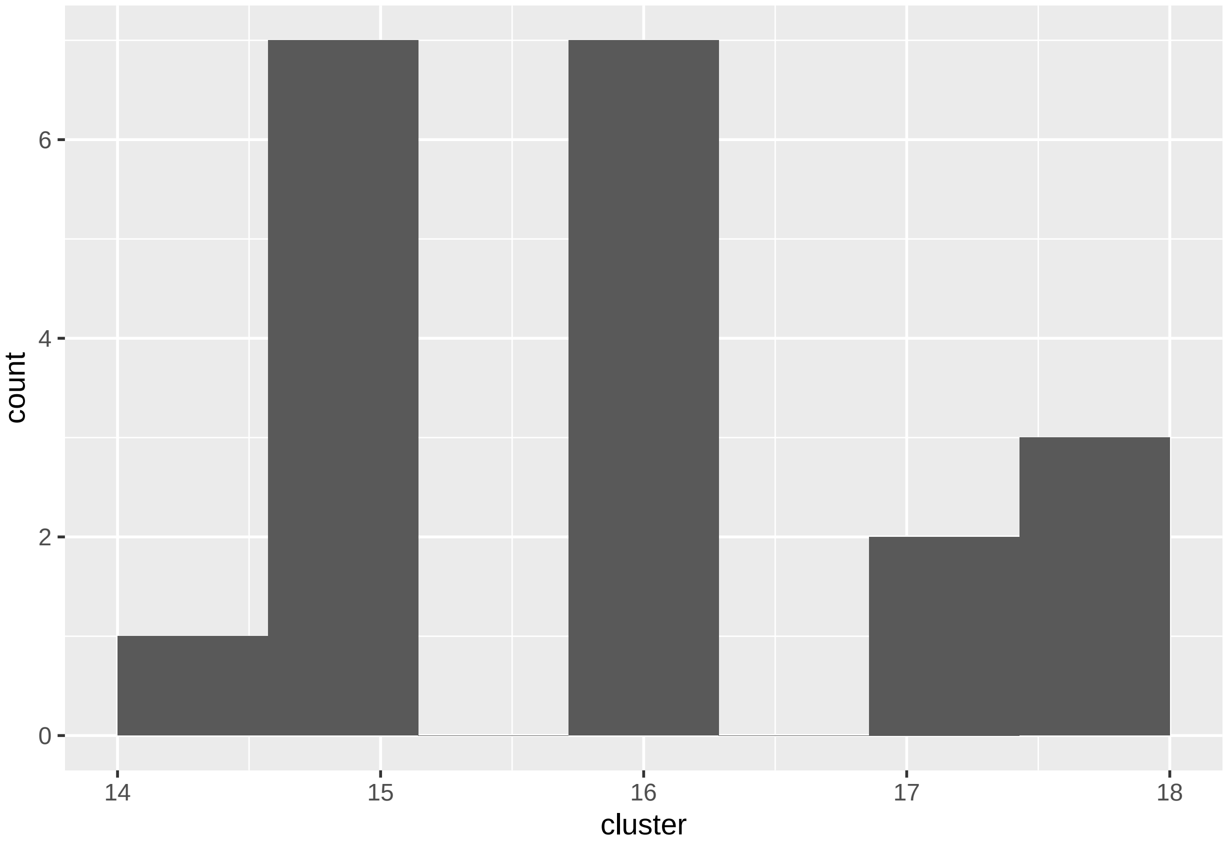


```
##
## 14 15 16 17 18
##  1  7  7  2  3
```

Both K=15 and K=16 were selected to be optimal by seven of the 20 coseq runs each. Thus, we select K=15 to continue.

We run coseq again with K=15, and save the analysis in a .RDS file to use for further analysis. Important: re-running the coseq chunk can lead to slightly different results, as clusters are re-defined each time.

We sort the clusters by expression (up -> down) and add the median log2FoldChange information with the custom function *clustersort()*.

Roots

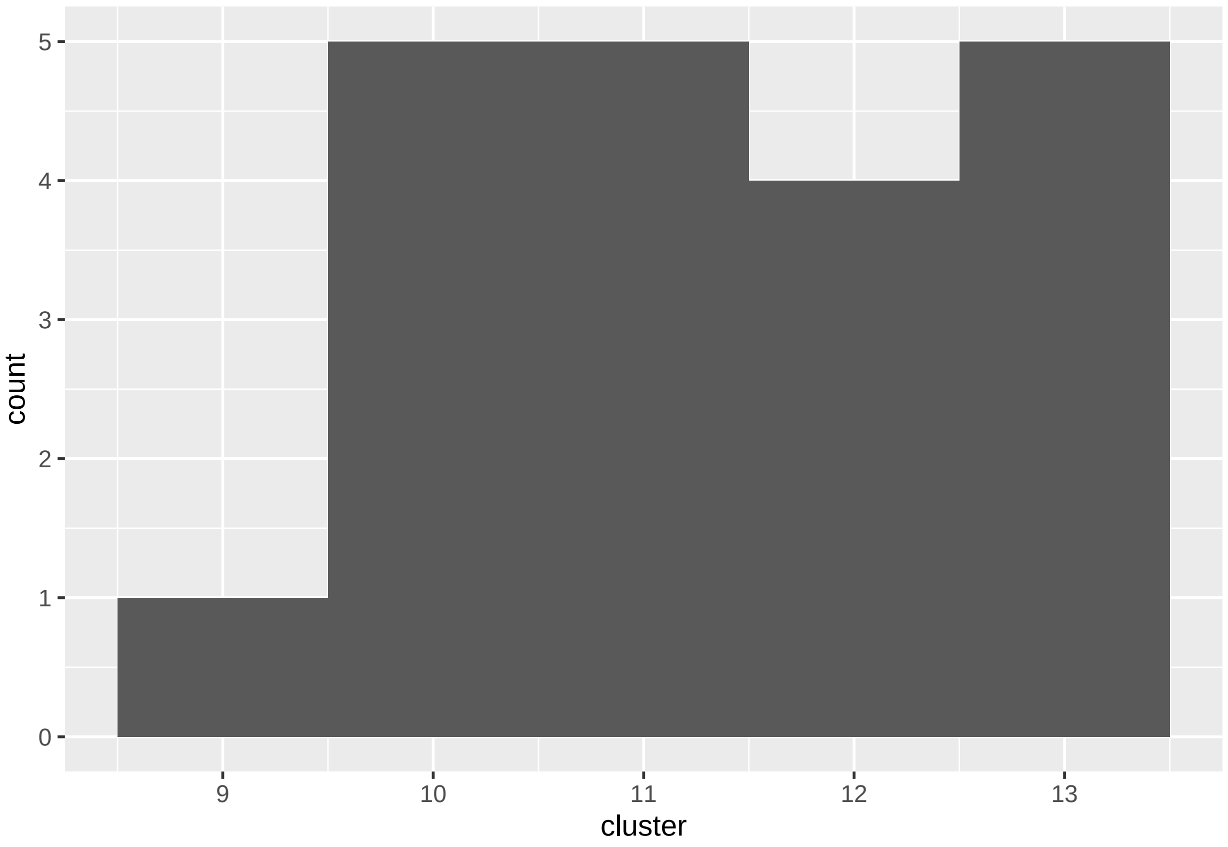


```
##
##  9 10 11 12 13
##  1  5  5  4  5
```

Both K=10, K=11 and K=13 and K=16 were selected to be optimal by five of the 20 coseq runs each. We chose K=11 to continue.

We run coseq again with K=11, sort the clusters as described for the shoots and save the analysis in a .RDS file to use for further analysis.

GO term analysis

Next, we performed a cluster-wise gene ontology (GO) enrichment analysis as implemented in the R-package clusterProfiler. We load the cluster data from the previously saved .RDS file and use the custom *GOanalysis()* function to do the GO enrichment analysis. For this, we use the functions *compareCluster()* and *simplify()* from clusterProfiler for the enrichment analysis.
As the GO enrichment analysis can also slightly change if re-run (as the underlying databases are regularly updated), we have saved the GO term analyses in .RDS files which we are using here for further analyses.
We then use the custom function *clusterheatmap()* to plot heatmaps showing the expression profile of each gene and the respective GO term assignments, using several functions adapted from clusterProfiler.
These heatmpats are reported in Supplementary Figure S3.

Shoots

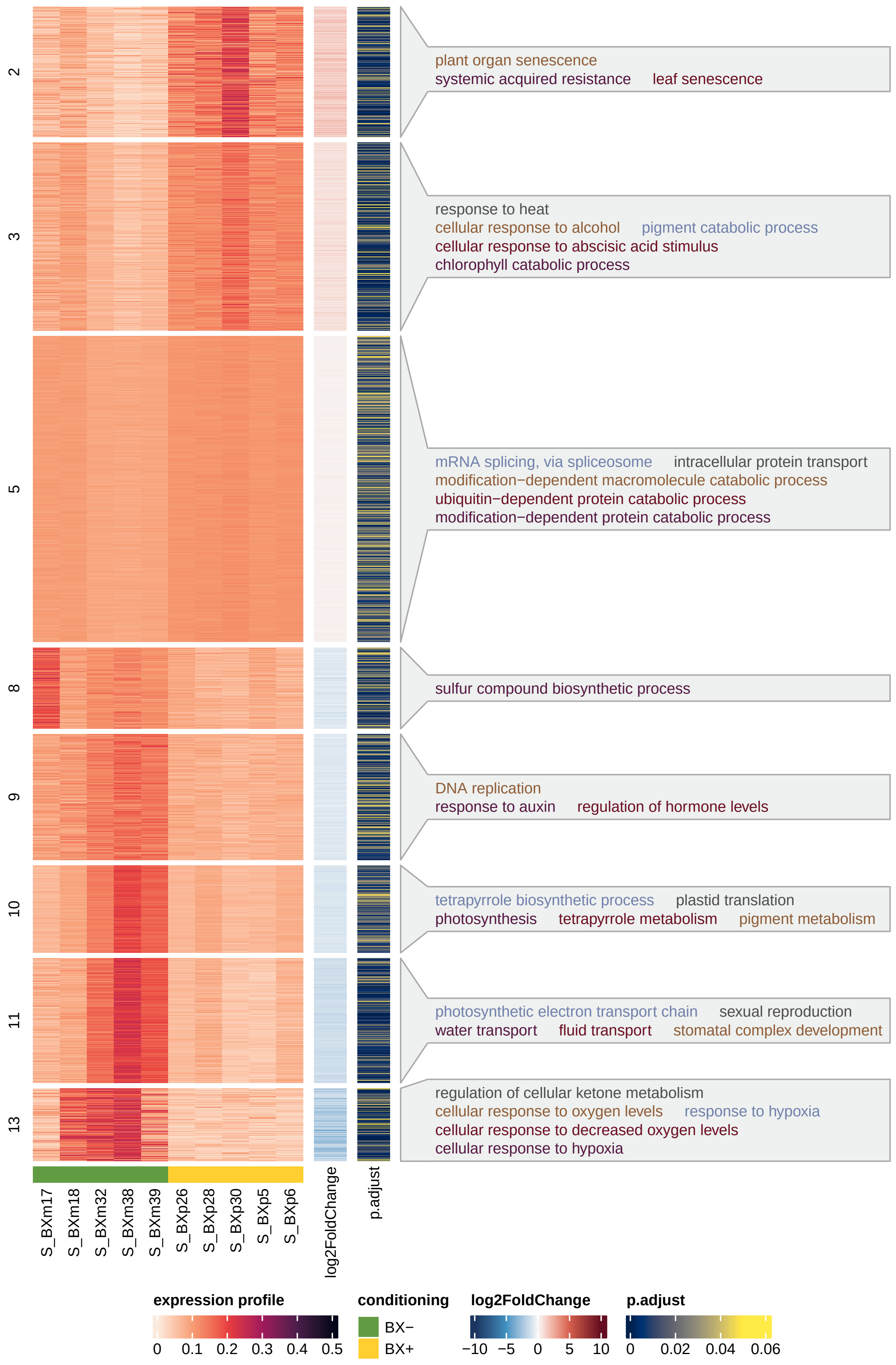

2
3
5
8
9
10
11
13
plant organ senescence
systemic acquired resistance
leaf senescence
response to heat
cellular response to alcohol
pigment catabolic process
cellular response to abscisic acid stimulus
chlorophyll catabolic process
mRNA splicing, via spliceosome
intracellular protein transport
modification−dependent macromolecule catabolic process
ubiquitin−dependent protein catabolic process
modification−dependent protein catabolic process
sulfur compound biosynthetic process
DNA replication
response to auxin
regulation of hormone levels
tetrapyrrole biosynthetic process
plastid translation
photosynthesis
tetrapyrrole metabolism
pigment metabolism
photosynthetic electron transport chain
sexual reproduction
water transport
fluid transport
stomatal complex development
regulation of cellular ketone metabolism
cellular response to oxygen levels
response to hypoxia
cellular response to decreased oxygen levels
cellular response to hypoxia
S_BXm17
S_BXm18
S_BXm32
S_BXm38
S_BXm39
S_BXp26
S_BXp28
S_BXp30
S_BXp5
S_BXp6
log2FoldChange
p.adjust
expression profile
0 0.1 0.2 0.3 0.4 0.5
conditioning
BX−
BX+
log2FoldChange
−10 −5 0 5 10
p.adjust
0 0.02 0.04 0.06

Roots

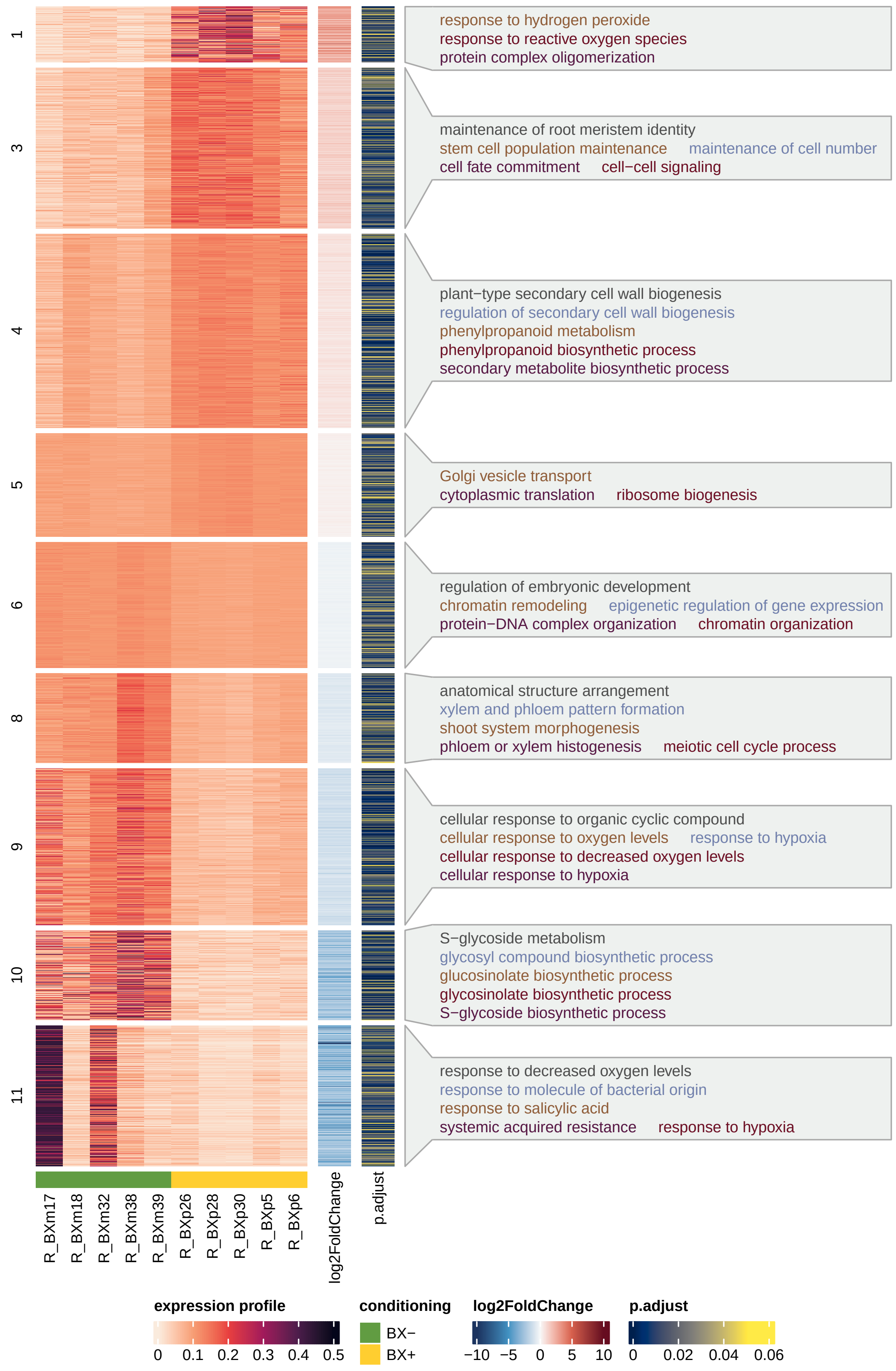
1
3
4
5
6
8
9
10
11
response to hydrogen peroxide
response to reactive oxygen species
protein complex oligomerization
maintenance of root meristem identity
stem cell population maintenance
maintenance of cell number
cell fate commitment
cell−cell signaling
plant−type secondary cell wall biogenesis
regulation of secondary cell wall biogenesis
phenylpropanoid metabolism
phenylpropanoid biosynthetic process
secondary metabolite biosynthetic process
Golgi vesicle transport
cytoplasmic translation
ribosome biogenesis
regulation of embryonic development
chromatin remodeling
epigenetic regulation of gene expression
protein−DNA complex organization
chromatin organization
anatomical structure arrangement
xylem and phloem pattern formation
shoot system morphogenesis
phloem or xylem histogenesis
meiotic cell cycle process
cellular response to organic cyclic compound
cellular response to oxygen levels
response to hypoxia
cellular response to decreased oxygen levels
cellular response to hypoxia
S−glycoside metabolism
glycosyl compound biosynthetic process
glucosinolate biosynthetic process
glycosinolate biosynthetic process
S−glycoside biosynthetic process
response to decreased oxygen levels
response to molecule of bacterial origin
response to salicylic acid
systemic acquired resistance
response to hypoxia
R_BXm17
R_BXm18
R_BXm32
R_BXm38
R_BXm39
R_BXp26
R_BXp28
R_BXp30
R_BXp5
R_BXp6
log2FoldChange
p.adjust
expression profile
0 0.1 0.2 0.3 0.4 0.5
conditioning
BX−
BX+
log2FoldChange
−10 −5 0 5 10
p.adjust
0 0.02 0.04 0.06

Dotplots of GO terms with high gene ratio

To focus only on the most important GO terms, we selected the two top GO terms from each cluster based on the highest gene ratio (= % of differentially regulated genes from all the genes assigned to a GO term). We have additionally filtered the data set to have meaningful GO terms - for example, if the two highest GO terms included the same upregulated genes and described very similar processes, we removed the similar GO term with lower amounts of differentially regulated genes. We visualize these GO terms with a dotplot.
These graphs are reported in the main part of the manuscript in Figure 4bd.

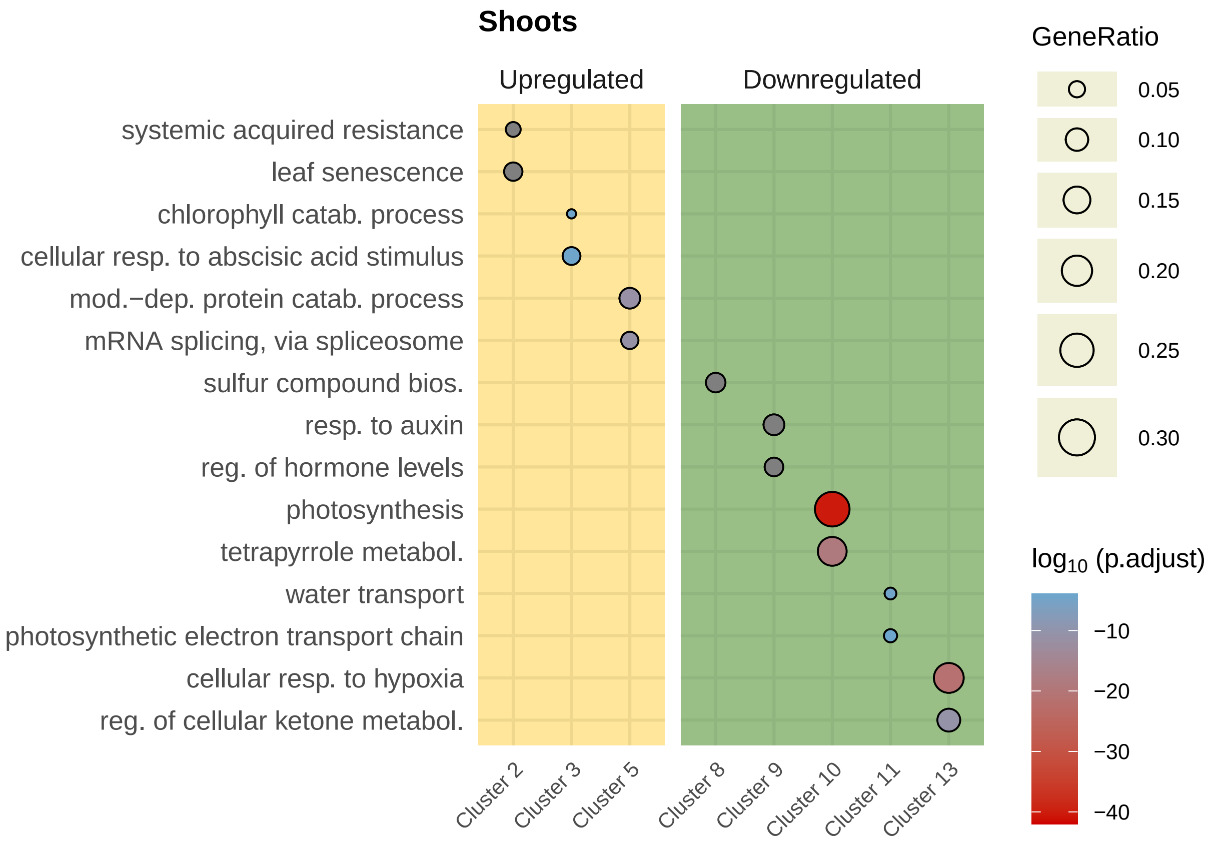

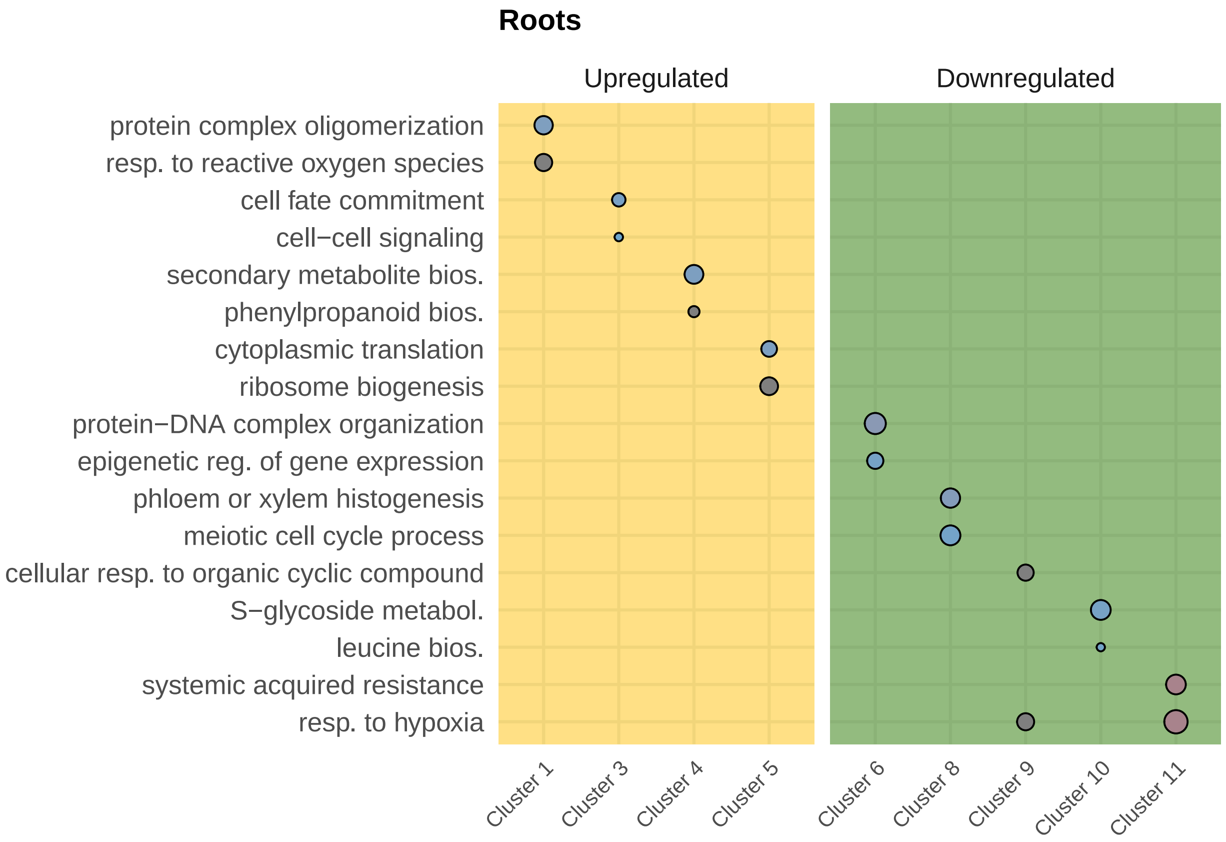
Roots
Upregulated
Downregulated
protein complex oligomerization
resp. to reactive oxygen species
cell fate commitment
cell−cell signaling
secondary metabolite bios.
phenylpropanoid bios.
cytoplasmic translation
ribosome biogenesis
protein−DNA complex organization
epigenetic reg. of gene expression
phloem or xylem histogenesis
meiotic cell cycle process
cellular resp. to organic cyclic compound
S−glycoside metabol.
leucine bios.
systemic acquired resistance
resp. to hypoxia
Cluster 1
Cluster 3
Cluster 4
Cluster 5
Cluster 6
Cluster 8
Cluster 9
Cluster 10
Cluster 11
