# Supplementary material for: Benzoxazinoid‐mediated microbiome feedbacks enhance Arabidopsis growth and defence
Source: New Phytol. 2026 Mar 17;250(5):3334–48. doi: 10.1111/nph.71098 (PMC13150306; doi:10.1111/nph.71098)
Supplement: Supplementary file 3 — Fig. S1 Benzoxazinoid concentrations in soils and Arabidopsis thaliana growth and development measurements on different soil batches. Fig. S2 Arabidopsis thaliana root growth on MBOA supplemented agar plates and rosette area on soil supplemented with MBOA. Fig. S3 Differentially abundant bacterial amplicon sequence variants between native BXplus and native BXminus soil. Fig. S4 Co‐expression analysis of genes expressed in Arabidopsis thaliana shoots and roots grown on BXplus compared to BXminus soil. Fig. S5 MBOA does not enhance resistance of Arabidopsis thaliana to Botrytis. Methods S1 Feedback experiments on soil. Methods S2 In vitro experiments. Methods S3 Microbiota profiling. Table S1 Setup of the different conditioning experiments. Table S2 Nutrient concentration of soil batches. Table S3 Setup of the different Arabidopsis thaliana experiments on soil. Table S4 List of differently abundant bacterial amplicon sequence variants in soil. Table S5 List of differently abundant bacterial amplicon sequence variants on roots of Arabidopsis thaliana. [file NPH-250-3334-s002.pdf]

New Phytologist

New Phytologist Supporting Information

Article title: Benzoxazinoid-mediated microbiome feedbacks enhance Arabidopsis growth and defence
Authors: Katja Stengele, Lea Stauber, Lisa Thoenen, Henry Janse van Rensburg, Viola D'Adda, Klaus Schlaeppi
Article acceptance date: 21 February 2026

The following Supporting Information is available for this article:

Dataset S1 Microbiota analysis

Dataset S2 Transcriptome analysis

Fig. S1 Benzoxazinoid concentrations in soils and *Arabidopsis thaliana* growth and development measurements on different soil batches.

Fig. S2 *Arabidopsis thaliana* root growth on MBOA supplemented agar plates and rosette area on soil supplemented with MBOA.

Fig. S3 Differentially abundant bacterial amplicon sequence variants between native BX_{plus} and native BX_{minus} soils.

Fig. S4 Co-expression analysis of genes expressed in *Arabidopsis thaliana* shoots and roots grown on BX_{plus} compared to BX_{minus} soil.

Fig. S5 MBOA does not enhance resistance of *Arabidopsis thaliana* to *Botrytis cinerea*.

Table S1 Setup of the different conditioning experiments.

Table S2 Nutrient concentration of soil batches.

Table S3 Setup of the different *Arabidopsis thaliana* experiments on soil.

Table S4 List of differently abundant bacterial amplicon sequence variants in soil.

Table S5 List of differently abundant bacterial amplicon sequence variants on roots of Arabidopsis thaliana.

Table S6 Shoot transcriptome

Table S7 Root transcriptome

Table S8 Shoot co-expression analysis.

Table S9 Root co-expression analysis.

Table S10 List of exemplary genes from the transcriptome analysis.

Methods S1 Feedback experiments on soil

Methods S2 In vitro experiments

Methods S3 Microbiota profiling

Dataset S1 Microbiota analysis. The Dataset S1 presents the R markdown output of the microbiota analysis. The raw sequencing data is available from ENA, the input data, R code and R markdown output are available from GitHub under https://github.com/PMI-Basel/Stengele_et_al_At_BX-feedbacks.

Dataset S2 Transcriptome analysis. The Dataset S2 presents the R markdown output of the transcriptome analysis. The raw sequencing data is available from ENA, input data, R code and R markdown output are available from GitHub under https://github.com/PMI-Basel/Stengele_et_al_At_BX-feedbacks.

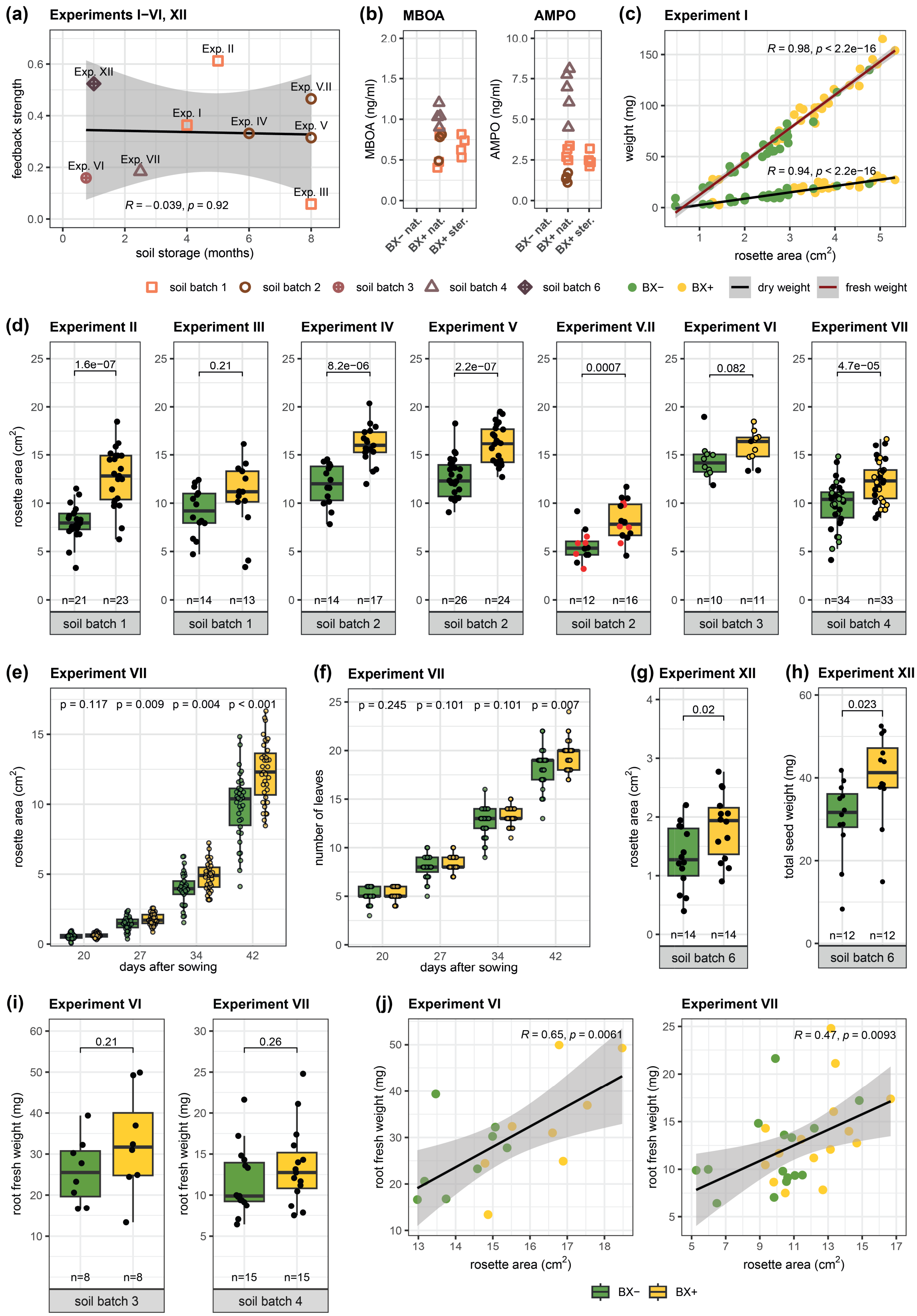
(a) Experiments I–VI, XII
feedback strength
soil storage (months)
Exp. II
Exp. XII
Exp. V.II
Exp. I
Exp. IV
Exp. V
Exp. VI
Exp. VII
Exp. III
R = −0.039, p = 0.92
(b) MBOA
AMPO
MBOA (ng/ml)
AMPO (ng/ml)
BX− nat.
BX+ nat.
BX+ ster.
(c) Experiment I
R = 0.98, p < 2.2e−16
R = 0.94, p < 2.2e−16
weight (mg)
rosette area (cm2)
soil batch 1
soil batch 2
soil batch 3
soil batch 4
soil batch 6
BX−
BX+
dry weight
fresh weight
(d) Experiment II
Experiment III
Experiment IV
Experiment V
Experiment V.II
Experiment VI
Experiment VII
rosette area (cm2)
1.6e−07
0.21
8.2e−06
2.2e−07
0.0007
0.082
4.7e−05
n=21 n=23
n=14 n=13
n=14 n=17
n=26 n=24
n=12 n=16
n=10 n=11
n=34 n=33
soil batch 1
soil batch 1
soil batch 2
soil batch 2
soil batch 2
soil batch 3
soil batch 4
(e) Experiment VII
p = 0.117 p = 0.009 p = 0.004 p < 0.001
rosette area (cm2)
days after sowing
(f) Experiment VII
p = 0.245 p = 0.101 p = 0.101 p = 0.007
number of leaves
days after sowing
(g) Experiment XII
0.02
rosette area (cm2)
n=14 n=14
soil batch 6
(h) Experiment XII
0.023
total seed weight (mg)
n=12 n=12
soil batch 6
(i) Experiment VI
Experiment VII
0.21
0.26
root fresh weight (mg)
n=8 n=8
n=15 n=15
soil batch 3
soil batch 4
(j) Experiment VI
Experiment VII
R = 0.65, p = 0.0061
R = 0.47, p = 0.0093
root fresh weight (mg)
rosette area (cm2)
BX−
BX+

Fig. S1 Benzoxazinoid concentrations in soils and *Arabidopsis thaliana* growth and development measurements on different soil batches. (a) Correlation between storage duration of soil batches and feedback strength for different experiments. Feedback strength was defined as the median shoot area of *Arabidopsis thaliana* (Arabidopsis) plants grown on BX_{plus} soil, divided by the median shoot area of Arabidopsis grown on BX_{minus} soil, subtracted by 1, so that positive values represent a positive feedback, i.e. larger rosettes developed on BX_{plus} soil. **(b)** Concentrations of MBOA and AMPO measured in native as well as sterilised conditioned soils. MBOA and AMPO concentrations had been measured in native ('nat.') BX_{plus} and BX_{minus} soils for soil batches 1, 2 and 4, and in sterilised ('ster.') BX_{plus} soil for soil batch 1; n = 4 for each treatment and soil batch. Samples that were below the detection limit for the given compound are not shown. **(c)** Correlation of Arabidopsis rosette area to shoot fresh weight (red line) and shoot dry weight (black line). **(d)** Arabidopsis rosette area from seven independent experiments. Note that the shoot area data for Experiment III is also shown in Fig. 2a. For Experiment V.II, the red dots indicate the plant replicates where the shoot and root transcriptome had been sequenced for the RNASeq analysis. For Experiments VI and VII, colored dots (yellow and green) represent plants where the root biomass was quantified, whereas black dots indicate no quantification of roots. The soil batch number, indicated at the bottom of each individual plot, denotes the independent conditioning events of the soil by growing maize. **(e)** Rosette area of plants over time. Note that the 42 day timepoint is also shown in the last panel in (c). Colored dots were chosen for better visibility only. **(f)** Visible number of leaves quantified from rosette photographs over time. For (e) and (f), the fdr adjusted *p*-values of two-sided student's t-tests are reported on top of each timepoint. **(g)** Arabidopsis rosette area of Experiment XII from 23 days after sowing. Note that plants from Experiment XII were grown under long-day conditions. **(h)** Total weight of all seeds collected per plant in Experiment XII. **(i)** Root fresh weights of Arabidopsis from two experiments and their **(j)** correlations with the rosette area data. In (a), (c) and (j), the pearson correlation coefficients and respective *p*-values are displayed within the plots. All boxplots in this figure report the median (horizontal line), and the 25th and 75th quartiles (bottom and top edges of the box, respectively). Lower and upper whiskers extend to 1.5 x the interquartile range from the bottom and top of the box, respectively. Individual data points are also plotted, representing biological replicates. The boxplot graphs of (d), (g), (h) and (i) further indicate the *p*-values of two-sided student's t-tests on top, and the replicate numbers (n) and the soil

batch number of the soil conditioning event at the bottom. The purpose and set-up of all Arabidopsis experiments on soil are also detailed in **Table S3**.

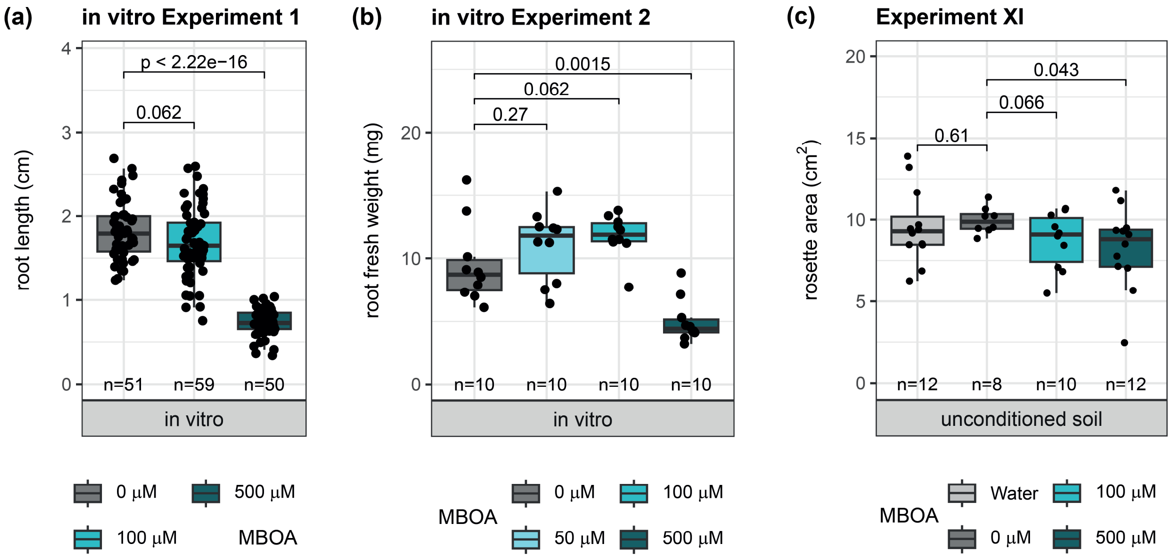

Fig. S2 ***Arabidopsis thaliana*** **root growth on MBOA supplemented agar plates and rosette area on soil supplemented with MBOA.** (**a**) Root length of *Arabidopsis thaliana* (Arabidopsis) germinated and grown for ten days on ½ MS agar plates supplemented with DMSO ('0 µm') or increasing amounts of MBOA dissolved in DMSO. (**b**) Root fresh weight of Arabidopsis grown for 21 days in total. Plants were pre-germinated for eight days on ½ MS agar with sucrose, and then grown for an additional 13 days on ½ MS agar plates supplemented with DMSO or increasing amounts of MBOA dissolved in DMSO. For the root fresh weight data, one data point represents the total root fresh weight from five plants grown on one plate. (**c**) Rosette area of Arabidopsis grown on un-conditioned soil amended with different MBOA concentrations. Before sowing of seeds, pots were either treated with water, DMSO ('0 µM') or MBOA dissolved in DMSO. All boxplots in this figure report the median (horizontal line), and the 25th and 75th quartiles (bottom and top edges of the box, respectively). Lower and upper whiskers extend to 1.5 x the interquartile range from the bottom and top of the box, respectively. Individual data points are also plotted, representing biological replicates. Replicate numbers (n) are reported below the boxplots, and the *p*-values of two-sided student's t-tests are reported on top of the boxplots for the respective comparisons. The purpose and set-up of all Arabidopsis experiments on soil are also detailed in **Table S3**.

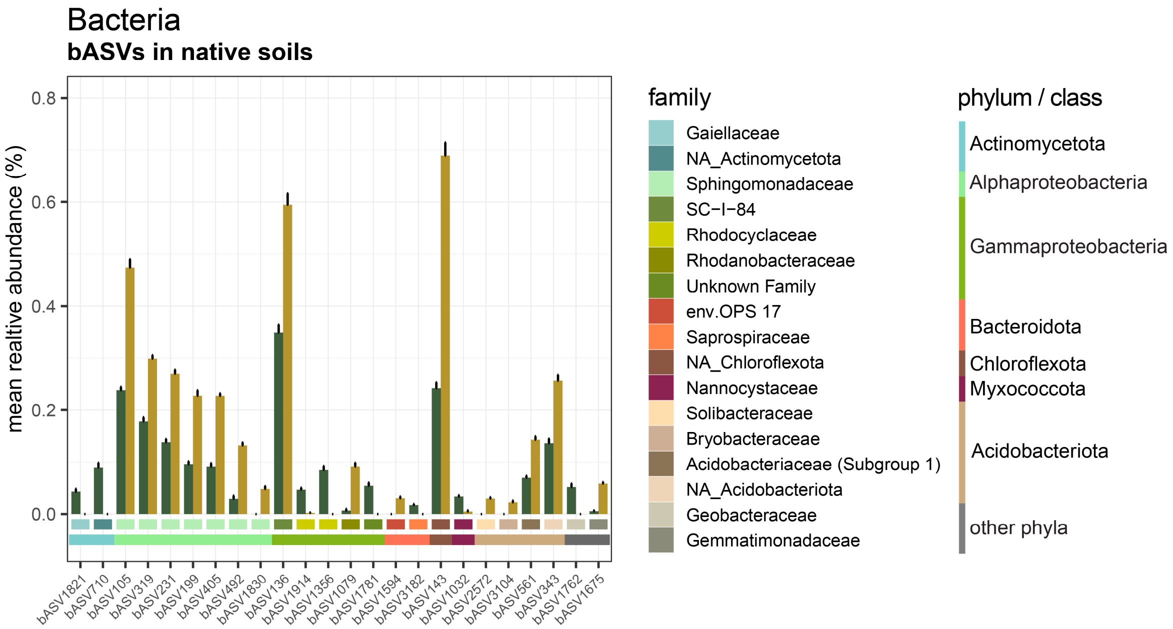

Fig. S3 Differentially abundant bacterial amplicon sequence variants in native soils. Abundance and taxonomy of differentially abundant bacterial amplicon sequence variants (bASVs) in native BX_{minus} (darkgreen bars) and BX_{plus} (lightbrown bars) soil. Error bars represent the standard error of the mean. The colored horizontal bars below the bargraphs indicate the bacterial family and phylum/class information (for the Pseudomonadota phylum, the class information is shown, ie. Alphaproteobacteria or Gammaproteobacteria).

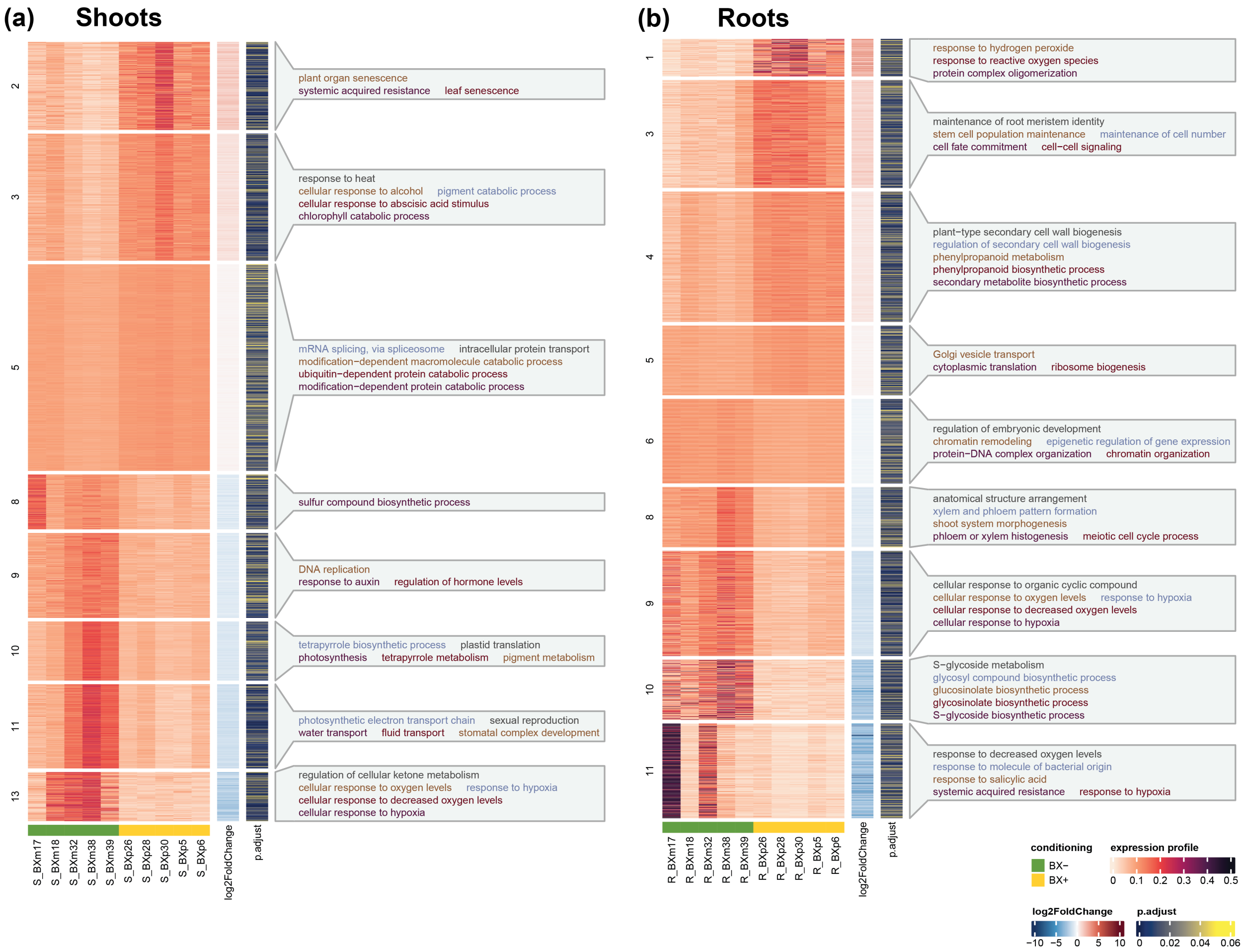

Fig. S4 Co-expression analysis of genes expressed in *Arabidopsis thaliana* shoots and roots grown on BX_{plus} compared to BX_{minus} soil. Heatmaps of co-expressed gene clusters in **(a)** shoots and **(b)** roots, as identified with coseq. Colors in heatmaps correspond to expression profiles, i.e. the proportion of normalized counts per gene. Adjacent color bars show the log_2FoldChange in expression, as well as the corresponding ajusted *p*-values as inferred with DESeq2. Cluster labels show the top five enriched Gene Ontology (GO) terms (biological processes, ajusted *p*-value < 0.01) in the corresponding clusters. The different GO term text colors are for better readability only. Full tables of all enriched GO terms in each cluster in shoots and roots are provided in **Tables S8** and **S9**.

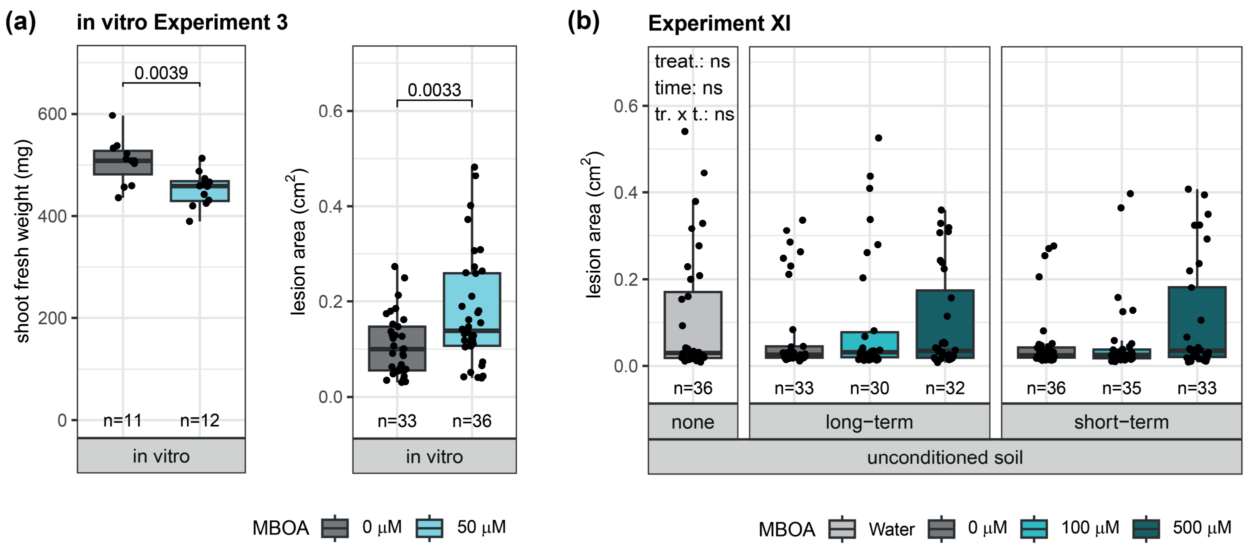

Fig. S5 MBOA does not enhance resistance of *Arabidopsis thaliana* against *Botrytis cinerea*. (a) Shoot fresh weight and *Botrytis cinerea* (Botrytis) infection of *Arabidopsis thaliana* (Arabidopsis) grown in a semi-hydroponic glass jar system. Plants had been pre-grown for a total of 21 days on sterile ½ MS plates before they were transplanted to glass jars. In the jars, plants were grown in ½ MS nutrient solution treated with DMSO ('0 µm') or 50 µm of MBOA dissovled in DMSO. After 36 days of total growth, three leaves per plant were infected with Botrytis, and the lesion area was quantified three days after the infection (graph on the right). The shoot biomass of the remaining shoot (= minus the three leaves that were infected) was also enumerated (graph on the left). The *p*-values of two-sided student's t-tests are reported in each graph. **(b)** Botrytis infection of Arabidopsis grown on un-conditioned soil amended with different MBOA concentrations. Plants were either untreated for the whole growth period ('Water'), treated with DMSO ('0 µm') or increasing amounts of MBOA dissolved in DMSO before sowing for a 'long-term' exposure, or treated with the same concentrations but only three days before the infection for a 'short-term' exposure. The significance levels of the ANOVA model testing the effect of the chemical treatment ('treat') and the timepoint of treatment application ('time') on lesion area are depicted in the top left corner of the graph. All boxplots in this figure report the median (horizontal line), and the 25th and 75th quartiles (bottom and top edges of the box, respectively). Lower and upper whiskers extend to 1.5 x the interquartile range from the bottom and top of the box, respectively. Individual data points are also plotted, representing individual plants for the

shoot fresh weight and individual leaves for the leasion area. In all graphs, replicate numbers (n) are reported below the boxplots. The purpose and set-up of all Arabidopsis experiments on soil are also detailed in **Table S3**.

Table S1 Setup of the different conditioning experiments.

One ‘Soil batch’ denotes one independent conditioning experiment, where parts of the collected soils indicated in the ‘Soil collection’ column were planted with wild-type B73 or *bx1*(B73) *Zea mays* (maize) for twelve weeks to condition the soil. For simplicity, we numbered the soil batches in their order of appearance in the text, with the original soil batch name indicated in brackets in the table. Soils were collected from three adjacent Agroscope research fields in Changins, Nyon, Switzerland (46°24'00.0"N 6°14'22.8"E), and the ‘Soil collection’ column denotes when the soil was collected and from which field (parcel). The three fields had the following cropping history: 2017 alfalfa, 2018 maize, 2019 winter wheat, 2020 strips of fodder peas, rapeseed, winter wheat, barley and maize, 2021 rapeseed and fodder peas, 2022 barley [parcel 29] and 2017 spring wheat, 2018 maize, 2019 sunflower, 2020 winter wheat, 2021 temporary grassland, 2022 winter wheat [parcels 30 and 31]. Maize plants were grown in round pots of different volumes (see ‘Pots’ column) and in different growth environments. For the fertilisation, the pots received a specified amount of nutrient solution once per week, starting with a low iron solution for the first four weeks and increasing to a high iron solution for most of the soil batches. Soil batch 4 consisted of two sub-batches, where plants were either fertilized with low iron followed by high iron (sub-batch ‘A’), or only with low iron fertilization for the total growth phase (sub-batch ‘B’). p. = parcel, w. = weeks, rel. hum. = relative humidity, int. = intensity.

Soil batch	Soil collection	Pots	Growth environment	Fertilisation	Growth conditions
soil batch 1 (BE01)	May 2019 (p. 29)	2 L	walk-in chamber[1]	4 w. low iron[3], 8 w. high iron[4]	16 h day at 26°C, 8 h night at 23°C, 50 % rel. hum., ~550 µmol m^{-2} s^{-1} light int.
soil batch 2 (BE02)	May 2019 (p. 29)	2 L	walk-in chamber[1]	4 w. low iron[3], 8 w. high iron[4]	16 h day at 26°C, 8 h night at 23°C, 50 % rel. hum., ~550 µmol m^{-2} s^{-1} light int.
soil batch 3 (BS07)	Sept. 2022 (p. 29/30)	3 L	greenhouse	4 w. low iron[3], 8 w. high iron[4]	16 h day at min. 23°C, 8 h night at min. 19°C, 300 - 500 µmol m^{-2} s^{-1} light int.
soil batch 4 (BS09)	Sept. 2022 (p. 29/30)	3 L	Phytotron[2] (climate chamber)	A: 4 w. low iron[3], 8 w. high iron[4] B: 12 w. low iron[3]	14 h day at 22°C, 10 h night at 18°C, 60 % rel. hum., ~550 µmol m^{-2} s^{-1} light int.
soil batch 5 (BS02)	Aug. 2020 (p. 30/31)	3 L	greenhouse	4 w. low iron[3], 8 w. high iron[4]	16 h day at min. 23°C, 8 h night at min. 19°C, 300 - 500 µmol m^{-2} s^{-1} light int.
soil batch 6 (BS05)	Dec. 2021 (p. 30)	3 L	greenhouse	4 w. low iron[3], 8 w. high iron[4]	16 h day at min. 23°C, 8 h night at min. 19°C, 300 - 500 µmol m^{-2} s^{-1} light int.

[1] Kaelte 3000 AG, Landquart, Switzerland [2] Phytotron Facility, University of Basel, Switzerland

[3] 100 mL of 0.2 % Plantaaktiv Typ K (Hauert HBG Duenger AG, Grossaffoltern, Switzerland) and 0.001 % Sequestrene Rapid (Maag, Westland Schweiz GmbH, Dielsdorf, Switzerland)

[4] 200 mL of 0.2 % Plantaaktiv Typ K, 0.02 % Sequestrene Rapid

Table S2 Nutrient concentration of soil batches.

Nutrient concentrations of four soil batches using water-based extractions, performed at the Labor für Boden- und Umweltanalytik (Steffisburg, Switzerland). The measurements were conducted at different times, where soil batches 1 and 2 had been analysed at the same time with 3 replicates per soil and conditioning, and soil batches 3 and 4 had been analysed together at a second time point with one replicate per soil and conditioning. The nutrient concentrations are given in mg per kg, and the standard deviation is indicated if applicable.

Soil batch	Conditioning	Calcium	Iron	Magnesium	Phosphorous	Potassium	pH
soil batch 1	BX +	141.8 ±7.5	10.1 ±0.5	11.3 ±0.3	2.1 ±0.1	15.0 ±1.0	7.2 ±0.04
	BX –	155.9 ±5.7	9.2 ±0.7	12.8 ±0.2	2.2 ±0	16.7 ±1.1	7.1 ±0.2
soil batch 2	BX +	169.2 ±3.4	8.4 ±0.3	10 ±0.4	2.1 ±0.1	15.6 ±0.8	7.4 ±0.1
	BX –	184.5 ±4.8	6 ±1.0	7.4 ±0.3	2.4 ±0.2	21.2 ±2.3	7.7 ±0.02
soil batch 3	BX +	430.1	1.6	19.2	6.0	70.1	7.7
	BX –	447.2	1.5	19.4	5.4	63.0	7.7
soil batch 4	BX +	316.4	2.3	12.7	4.0	43.0	7.9
	BX –	343.9	2.0	13.6	3.8	45.0	7.7

Table S3 Setup of the different *Arabidopsis thaliana* experiments on soil.

For each experiment, the soil batch, pot size and growth length are indicated. For details on the conditioning of each soil batch, see **Table S1**. Soil batches were stored at 4°C and the soil storage column indicates how long the soils were stored before set up of the experiments (in months), while the growth column indicates how long the *Arabidopsis thaliana* plants were grown. The fertilisation column indicates how many times the plants were fertilised during the growth phase for each experiment. As measurements, we quantified shoot growth at the end of the experiment, and for certain experiments we additionally recorded flowering, the root biomass, collected roots for a microbiota analysis, collected root and shoot material for a transcriptome analysis, assessed priming of *PR1* or infected the leaves with the fungus *Botrytis cinerea*. For Exp. X, both fertilizer sub-batches of soil batch 4 were used (see **Table S1**), and Botrytis infection was assessed by analysing the plants from both sub-batches together for each conditioning. uncond. = unconditioned.

Experiment	Soil batch	Pots	Soil storage	Fertili-sation[1]	Growth (weeks)	Measurements
Exp. I	soil batch 1	round[2]	4	-	7	Shoot growth
Exp. II	soil batch 1	square[3]	5	3x	7	Shoot growth
Exp. III	soil batch 1	square[4]	8	1x	6	Shoot growth, Root microbiota
Exp. IV	soil batch 2	square[3]	6	1x	6.5	Shoot growth
Exp. V	soil batch 2	square[4]	8	1x	8	Shoot growth
Exp. V.II	soil batch 2	square[4]	8	1x	7	Shoot growth, Transcriptome
Exp. VI	soil batch 3	square[4]	0.75	-	7	Shoot growth, Root biomass
Exp. VII	soil batch 4	square[4]	2.5	2x	7	Shoot growth, Root biomass, *PR1* priming
Exp. VIII	soil batch 3	square[4]	1	2x	6	Botrytis infection
Exp. IX	soil batch 5	square[3]	2.5	2x	5	Botrytis infection
Exp. X	soil batch 4	square[3]	0.5	2x	6	Botrytis infection
Exp. XI	uncond. soil	square[4]	NA	1x	6	Botrytis infection
Exp. XII	soil batch 6	square[4]	1	-	5	Shoot growth, Flowering

[1] Fertilisation by watering with 1/3 half strength Hoagland solution diluted in tap water.
[2] 5.5 x 5 cm pot size [3] 8 x 8 x 8.5 cm pot size [4] 6 x 6 x 6.5 cm pot size

Table S4 List of differently abundant bacterial amplicon sequence variants in soil.

Relative abundance and taxonomic information of differently abundant bacterial amplicon sequence variants (bASVs) from BX_{plus} and BX_{minus} soil. Due to spatial restrictions, kingdom and order are not shown. The phylum/class column reports the phylum information, but in the case of the Pseudomonadota phylum, the class information is given (Gammaproteobacteria, Alphaproteobacteria). The mean relative abundances of each bASV in native BX_{plus} and BX_{minus} soil are indicated, ordered by decreasing abundance in the BX_{plus} condition. bASVs with higher abundance (abund.) in native BX_{plus} soil are shown in bold. C. = candidatus. sg1 = subgroup 1.

ASV	phylum / class	family	genus	abund. BX+	abund. BX–
**bASV143**	Chloroflexota	unassigned	unassigned	0.69%	0.24%
**bASV136**	Gammaproteobacteria	SC-I-84	unassigned	0.59%	0.35%
**bASV105**	Alphaproteobacteria	Sphingomonadaceae	Sphingomonas	0.47%	0.24%
**bASV319**	Alphaproteobacteria	Sphingomonadaceae	Sphingomonas	0.30%	0.18%
**bASV231**	Alphaproteobacteria	Sphingomonadaceae	Sphingomonas	0.27%	0.14%
**bASV343**	Acidobacteriota	unassigned	unassigned	0.26%	0.14%
**bASV199**	Alphaproteobacteria	Sphingomonadaceae	Sphingomonas	0.23%	0.10%
**bASV405**	Alphaproteobacteria	Sphingomonadaceae	Sphingomonas	0.23%	0.09%
**bASV561**	Acidobacteriota	Acidobacteriaceae (sg1)	Occallatibacter	0.14%	0.07%
**bASV492**	Alphaproteobacteria	Sphingomonadaceae	Sphingomonas	0.13%	0.03%
**bASV1079**	Gammaproteobacteria	Rhodanobacteraceae	Rhodanobacter	0.09%	0.01%
**bASV1675**	Gemmatimonadota	Gemmatimonadaceae	unassigned	0.06%	0.01%
**bASV1830**	Alphaproteobacteria	Sphingomonadaceae	Croceibacterium	0.05%	0%
**bASV1594**	Bacteroidota	env.OPS 17	unassigned	0.03%	0%
**bASV2572**	Acidobacteriota	Solibacteraceae	C. Solibacter	0.03%	0%
**bASV3104**	Acidobacteriota	Bryobacteraceae	Bryobacter	0.02%	0%
bASV1032	Myxococcota	Nannocystaceae	Nannocystis	0%	0.03%
bASV1914	Gammaproteobacteria	Rhodocyclaceae	C. Accumulibacter	0%	0.05%
bASV710	Actinomycetota	unassigned	unassigned	0%	0.09%
bASV1356	Gammaproteobacteria	Rhodocyclaceae	Azovibrio	0%	0.08%
bASV1762	Thermodesulfobacteriota	Geobacteraceae	Geomonas	0%	0.05%
bASV1781	Gammaproteobacteria	Unknown Family	Acidibacter	0%	0.05%
bASV1821	Actinomycetota	Gaiellaceae	Gaiella	0%	0.04%
bASV3182	Bacteroidota	Saprospiraceae	unassigned	0%	0.02%

Table S5 List of differently abundant bacterial amplicon sequence variants on roots of *Arabidopsis thaliana*.

Relative abundance and taxonomic information of differently abundant bacterial amplicon sequence variants (bASVs) of *Arabidopsis thaliana* root communities from native BX_{plus} and BX_{minus} soil. Due to spatial restrictions, kingdom and order are not shown. The phylum/class column reports the phylum information, but in the case of the Pseudomonadota phylum, the class information is given (Gammaproteobacteria). The mean relative abundances of each bASV on roots grown in native BX_{plus} and BX_{minus} soil are indicated, ordered by decreasing abundance in the BX_{plus} condition. bASVs with higher abundance (abund.) on roots from native BX_{plus} soil are shown in bold.

ASV	phylum / class	family	genus	abund. BX+	abund. BX–
**bASV39**	Gammaproteobacteria	Oxalobacteraceae	Massilia	2.09%	0.59%
**bASV511**	Bacteroidota	env.OPS 17	unassigned	0.20%	0.02%
**bASV453**	Actinomycetota	67-14	unassigned	0.11%	0.01%
bASV698	Myxococcota	Sandaracinaceae	unassigned	0.03%	0.11%
bASV138	Gammaproteobacteria	Cellvibrionaceae	Cellvibrio	0.01%	0.28%
bASV117	Gammaproteobacteria	Oxalobacteraceae	Massilia	0.01%	0.79%
bASV830	Gammaproteobacteria	Comamonadaceae	unassigned	0%	0.10%
bASV1246	Myxococcota	Sandaracinaceae	Sandaracinus	0%	0.06%
bASV164	Gammaproteobacteria	Oxalobacteraceae	Massilia	0%	0.58%
bASV791	Spirochaetota	Spirochaetaceae	Spirochaeta 2	0%	0.10%
bASV1445	Gammaproteobacteria	Rhodanobacteraceae	Ahniella	0%	0.05%

The **Supplementary Tables S6 to S10** are in a common excel file with the following work sheets:

Table S6 Shoot transcriptome. List of significantly differentially expressed genes ($p_{adj} < 0.05$, $|\log_2$ fold change$| > 1$) in shoots as inferred with DESeq2. The work sheet lists the Gene-ID, its base mean of expression, the $\log_2$ fold change and its standard error, the DESeq statistic, its *p*-value and adjusted *p*-value.

Table S7 Root transcriptome. List of significantly differentially expressed genes ($p_{adj} < 0.05$, $|\log_2$ fold change$| > 1$) in roots as inferred with DESeq2. The work sheet lists the Gene-ID, its base mean of expression, the $\log_2$ fold change and its standard error, the DESeq statistic, its *p*-value and adjusted *p*-value.

Table S8 Shoot co-expression analysis. The table lists the enriched gene ontology (GO) terms for each cluster of co-expressed genes in shoots, detailing the GO term ID, the description (biological processes), the gene ratio, background gene ratio, *p*-value, adjusted *p*-value and Q-value together with the gene IDs of the identified genes and their count per cluster.

Table S9 Root co-expression analysis. The table lists the enriched gene ontology (GO) terms for each cluster of co-expressed genes in roots, detailing the GO term ID, the description (biological processes), the gene ratio, background gene ratio, *p*-value, adjusted *p*-value and Q-value together with the gene IDs of the identified genes and their count per cluster.

Table S10 List of exemplary genes from the transcriptome analysis. The table lists exemplary genes for root and shoot tissue that were either upregulated or downregulated in plant tissue grown on BX_{plus} soil compared to plant tissue grown on BX_{minus} soil. The Gene ID, gene name, tissue, cluster affiliation, $\log_2$ fold change, adjusted *p*-value as well as the gene description gathered from TAIR are given for each listed gene.

Methods S1 Feedback experiments on soil

We have performed several experiments (Exp. I to Exp. XII) with slightly different setups, which are detailed in **Table S3**. While the general procedure, which applies to all experiment, is described in the main text, here we explain additional details of particular experiments:

Experiment III: Fractions of the soil/sand mixtures for BX_{plus} and BX_{minus} soil were additionally sterilised by X-radiation (36 – 40 kGy, Synergy Health AG, Däniken, Switzerland) before filling to pots. Also, seeds for Experiment III were sterilised (Protocol, see Experiment 2 in Methods S2) before sowing and pots were watered with autoclaved tap water for the whole course of the experiment.

Experiment VII: The number of leaves was counted from images of the rosettes taken at different time points.

Experiment XI: This experiment, testing the application of MBOA to soil, was conducted with unconditioned soil collected from the field in Changins and was performed testing 'long-term' and 'short-term' exposure of MBOA. One group of control pots remained untreated and only received water for the duration of the experiment. Half of the treated (DMSO and different concentrations of MBOA dissolved in DMSO) pots were used for 'long-term' exposure to MBOA and they were treated before sowing. The other half of the treated pots were used to test 'short-term' exposure and they were treated after six weeks of plant growth (and three days before infection with Botrytis; see *Infections with Botrytis cinerea* in the main Materials and Methods). The treatments consisted of applications of 12 mL tap water containing 0, 100 or 500 µM MBOA in 0.06% DMSO. The rosette area data reported in this manuscript for Experiment XI were of 'long-term' exposed plants, while the Botrytis infection was performed on both 'long-term' and 'short-term' exposed plants.

Experiment XII: In this experiment, plants had been grown in a climate chamber (Sanyo, Moriguchi, Japan) equipped with fluorescent bulbs under long-day conditions (16 h day at 21°C and light intensities between 100 and 200 µmol m^{-2} s^{-1}, and 8 h night at 18°C). To score effects of the soil conditioning on flowering, we recorded at three time points the following three developmental stages: plants that (i) did not bolt yet, (ii) showed onset of bolting and (iii) had bolted. For the plants that had bolted, we further measured the heights of the developing flower

stalks using a ruler. Plants that had not bolted at the third timepoint were discarded, and the rest of the plants were grown to seed, and we collected the seeds for each plant separately (n=12 on each soil). Seed yields were scored by measuring the total seed weight on an analytical balance and by spreading the seeds on a grid paper followed by manually counting them using a mechanical tally counter. Finally, we calculated the average weight of 100 seeds based on the total seed number and total seed weights.

Methods S2 In vitro experiments

Experiment 1: The seeds were sterilised for four hours with chlorine gas (Lindsey III *et al.*, 2017), and then directly sown to ½ Murashige and Skoog (MS; Duchefa, Haarlem, the Netherlands) plates containing 1.5 % plant agar (Duchefa, Haarlem, the Netherlands) supplemented with MBOA (Sigma-Aldrich, St. Louis, USA) at different concentrations (0, 100 or 500 µM) or no MBOA as control. MBOA was dissolved in DMSO (Sigma-Aldrich), which was kept constant at 500 µL per L ½ MS agar in each treatment including the control (resulting in 0.05 % DMSO). 12 to 16 seeds were sown per plate and stratified for three days at 4 °C. Seed germination was recorded for the first four days after germination, and primary root length was quantified ten days after sowing.

Experiment 2: Seeds were surface sterilised with 70% Ethanol + 0.1 % Triton-X100 for 1 minute, hypochlorite solution (<5 %, Potz, Migros, Zurich, Switzerland) + 0.1 % Triton-X100 for 12 minutes, and washed three times with sterile milliQ water. Seeds were sown onto ½ MS agar plates containing 1 % sucrose, stratified for three days at 4 °C and germinated for eight days. The seedlings were then transferred to ½ MS plates containing 825.75 µL DMSO per L ½ MS agar (0.08 % DMSO) as control or plates containing the same amount of DMSO with different MBOA concentrations (0, 50, 100 or 500 µM). Five seedlings were transferred onto each plate and grown for additional 13 days, before the total root and shoot material per plate was harvested to determine the fresh weight (meaningful shoot area data could not be quantified as plants grow vertically on the agar medium and shoots do not grow flat like on pots filled with soil).

Experiment 3: This experiment was conducted in a semi-hydroponic growth system (McLaughlin *et al.*, 2023). Seeds had been sterilised as described for in vitro experiment 2, and grown on ½ MS for seven days. Seedlings were then transplanted to fresh ½ MS plates and grown for another

14 days. Two seedlings were then transplanted to the jar system described in McLaughlin *et al*. (2023), but with a modified volume of 32.5 mL ½ MS per jar. The ½ MS added to the jars was supplemented with 100 µL DMSO per L ½ MS agar (0.01 % DMSO) either containing 0 or 50 µM MBOA. Every nine days of growth in the jars, the growth medium was replaced with fresh 32.5 mL of ½ MS including freshly prepared MBOA or DMSO control treatments. After a total of 28 days in the jars, one plant was harvested from the jar for sample material collection (consistent with the in vitro Experiment 2, we quantified shoot fresh weights). One day later, the remaining plant was used for *Botrytis* infection (see *Infections with Botrytis cinerea* in the main Materials and Methods). After infection, the lid was taped to ensure high humidity for infection. Jars were then placed into growth chambers equipped with LED light under low light conditions (~ 50 µmol m^{-2} s^{-1}) and the lesion area was quantified three days after infection.

Methods S3 Microbiota profiling

Sampling: The soil samples were taken from the middle of unplanted pots that had been included in Experiment III, and a subsample of approximately 250 mg was used for DNA extraction. For roots, we harvested them from the pot and sampled a 5 cm long root fragment starting from 1 cm below the rosette base, which included both primary and lateral roots. These root segments were washed in 10 mM $MgCl_2$, blot dried with tissue paper, snap frozen in liquid nitrogen, and stored at -80 °C. Root samples were then lyophilized (FreeZone Plus; Labconco Corporation, Kansas City, USA) for three days and ground for 5 minutes with a ball mill (Mixer Mill MM 400; Retsch GmbH, Haan, Germany).

DNA extraction: From all samples, we extracted the DNA with the NucleoSpin Soil DNA extraction kit (Macherey-Nagel, Düren, Germany), and quantified the DNA concentrations with the AccuClear® Ultra High Sensitivity dsDNA Quantitation Kit (Biotium, Fremont, USA). We normalized the concentration to 0.2 ng/µL for soil samples and 2 ng/µL for root samples, or used undiluted samples if DNA concentrations were lower.

PCR and library preparation: We performed two-step PCR amplification and barcoding of the bacterial 16S rRNA gene region and the fungal ITS spacer region as was previously described in Gfeller *et al.*, (2023). We used the same cycling profiles, but with 3 minutes of initial denaturation.

The reaction of the first 16S rRNA gene PCR was composed of 1x 5Prime HotMasterMix (Quantabio, Beverly, USA), 0.3 % BSA, 300 nM of each primer and 10 ng root DNA or 1 ng soil DNA. The second PCR contained 6 µL of purified PCR product (using SPRIselect beads; Beckman Coulter Life Sciences, Indianapolis, USA) and individual barcoded primer pairs for each sample. The ITS PCR reactions contained 200 nM of each primer but were otherwise the same as the 16S rRNA gene reactions. The products from the second PCR were then purified with SPRIselect beads, and pooled to 25 ng DNA per sample for the 16S samples, and to 2.5 ng DNA or as much as was available per sample for the ITS samples. Both pools were bead purified again and pooled for the final library.

Sequencing and bioinformatic analysis: The library was sequenced with the MiSeq reagent kit v3 at the Next Generation Sequencing Platform (University of Bern) using the 2x 300 bp pair-end sequencing protocol (Illumina Inc., San Diego, USA). The MiSeq 16S rRNA gene and ITS reads were quality checked with FastQC v0.11.8 (Babraham Institute, Cambridge, United Kingdom) and demultiplexed with cutadapt v2.10 (Martin, 2011). Quality filtering, read merging and amplicon sequence variants (ASV) clustering was implemented in R v4.0.0 (R Core Team, 2017) using the package dada2 v1.16.0 (Callahan *et al.*, 2016). Bacterial taxonomies were assigned using a DADA2 formatted training set (silva_nr99_v138.2_toSpecies_trainset.fa.gz) from the SILVA database (Quast *et al.*, 2013) with a naïve Bayesian classifier, and fungal taxonomies were assigned with a training set (sh_general_release_dynamic_02.02.2019.fasta) from the UNITE database (Abarenkov *et al.*, 2024).

Supplementary References

Abarenkov K, Nilsson RH, Larsson K-H, Taylor AFS, May TW, Frøslev TG, Pawlowska J, Lindahl B, Põldmaa K, Truong C, *et al.* 2024. The UNITE database for molecular identification and taxonomic communication of fungi and other eukaryotes: sequences, taxa and classifications reconsidered. *Nucleic Acids Research* **52**: D791–D797.

Callahan BJ, McMurdie PJ, Rosen MJ, Han AW, Johnson AJA, Holmes SP. **2016**. DADA2: High-resolution sample inference from Illumina amplicon data. *Nature Methods* **13**: 581–583.

Gfeller V, Waelchli J, Pfister S, Deslandes-Hérold G, Mascher F, Glauser G, Aeby Y, Mestrot A, Robert CA, Schlaeppi K, *et al.* 2023. Plant secondary metabolite-dependent plant-soil feedbacks can improve crop yield in the field. *eLife* **12**: e84988.

Lindsey III BE, Rivero L, Calhoun CS, Grotewold E, Brkljacic J. **2017**. Standardized method for high-throughput sterilization of Arabidopsis seeds. *Journal of Visualized Experiments*: 56587.

Martin M. **2011**. Cutadapt removes adapter sequences from high-throughput sequencing reads. *EMBnet.journal* **17**: 10–12.

McLaughlin S, Joller C, Siffert A, Stirnemann EM, Sasse J. **2023**. A Versatile Glass Jar System for Semihydroponic Root Exudate Profiling. *Journal of Visualized Experiments*: 66070.

Quast C, Pruesse E, Yilmaz P, Gerken J, Schweer T, Yarza P, Peplies J, Glöckner FO. **2013**. The SILVA ribosomal RNA gene database project: improved data processing and web-based tools. *Nucleic Acids Research* **41**: D590–D596.

R Core Team. **2017**. *R: A language and environment for statistical computing*. Vienna, Austria: R Foundation for Statistical Computing.
